# Further confirmation of *netrin 1 receptor* (*DCC*) as a depression risk gene *via* integrations of multi-omics data

**DOI:** 10.1038/s41398-020-0777-y

**Published:** 2020-03-17

**Authors:** Hui-Juan Li, Na Qu, Li Hui, Xin Cai, Chu-Yi Zhang, Bao-Liang Zhong, Shu-Fang Zhang, Jing Chen, Bin Xia, Lu Wang, Qiu-Fang Jia, Wei Li, Hong Chang, Xiao Xiao, Ming Li, Yi Li

**Affiliations:** 1 Key Laboratory of Animal Models and Human Disease Mechanisms of the Chinese Academy of Sciences and Yunnan Province, Kunming Institute of Zoology, Chinese Academy of Sciences, Kunming, Yunnan China; 2 Kunming College of Life Science, University of Chinese Academy of Sciences, Kunming, Yunnan China; 3 Affiliated Wuhan Mental Health Center, Tongji Medical College, Huazhong University of Science and Technology, Wuhan, Hubei China; 4 Research Center for Psychological and Health Sciences, China University of Geosciences, Wuhan, Hubei China; 5 Suzhou Guangji Hospital, The Affiliated Guangji Hospital of Soochow University, Suzhou, Jiangsu China; 6 Department of Blood Transfusion, The Second Affiliated Hospital of Kunming Medical University, Kunming, Yunnan China

**Keywords:** Depression, Comparative genomics

## Abstract

Genome-wide association studies (GWAS) of major depression and its relevant biological phenotypes have been extensively conducted in large samples, and transcriptome-wide analyses in the tissues of brain regions relevant to pathogenesis of depression, e.g., dorsolateral prefrontal cortex (DLPFC), have also been widely performed recently. Integrating these multi-omics data will enable unveiling of depression risk genes and even underlying pathological mechanisms. Here, we employ summary data-based Mendelian randomization (SMR) and integrative risk gene selector (iRIGS) approaches to integrate multi-omics data from GWAS, DLPFC expression quantitative trait loci (eQTL) analyses and enhancer-promoter physical link studies to prioritize high-confidence risk genes for depression, followed by independent replications across distinct populations. These integrative analyses identify multiple high-confidence depression risk genes, and numerous lines of evidence supporting pivotal roles of the *netrin 1 receptor* (*DCC*) gene in this illness across different populations. Our subsequent explorative analyses further suggest that *DCC* significantly predicts neuroticism, well-being spectrum, cognitive function and putamen structure in general populations. Gene expression correlation and pathway analyses in DLPFC further show that *DCC* potentially participates in the biological processes and pathways underlying synaptic plasticity, axon guidance, circadian entrainment, as well as learning and long-term potentiation. These results are in agreement with the recent findings of this gene in neurodevelopment and psychiatric disorders, and we thus further confirm that *DCC* is an important susceptibility gene for depression, and might be a potential target for new antidepressants.

## Introduction

A primary current challenge in the psychiatry field is to dissect the underlying neurobiological basis of common mental illnesses such as major depression, which is said to be one of the ten most disabling conditions in the world^1^. Given the substantial heritability of major depression (~37%)^2^, the application of human genetic approaches is believed to both promote the understanding of its biological mechanisms and benefit discovery and development of effective clinical treatment strategies. Indeed, recent genome-wide association studies (GWAS) of depressive subjects and healthy controls have identified multiple statistically robust loci^3,4^, providing numerous candidates for in-depth exploration of its pathological mechanisms. Notably, there is growing consensus on such “in-depth functional exploration” of psychiatric disease-related loci, and several critical steps have been raised: (1) probe the risk genes from risk locus; (2) depict (novel or refined) disease mechanisms based on the risk genes; (3) assess the druggability of the risk gene product itself or a proximate pathway^5,6^. This research regimen has been widely applied in recent years, however, the fact that majority of the disease risk loci identified by GWAS reside in the noncoding genomic regions has significantly hampered the accomplishments in elucidating their biological and pathological impacts. Fortunately, accumulating studies have found that noncoding variations of complex diseases tend to be associated with mRNA expression^7^, and analyzing the expression quantitative trait loci (eQTL) effects of the risk alleles in relevant tissues is therefore a plausible strategy to probe the risk genes from risk locus^8–10^. In line with this, several integrative analyses using GWAS and brain eQTL data have revealed susceptibility genes and potential biological mechanisms for psychiatric disorders^11–13^.

For depression, recent genome-wide linkage disequilibrium (LD) score regression analyses have shown strong genetic correlations between this illness and multiple quantifiable behavioral phenotypes (e.g., emotional traits and cognitive functions)^4^. In addition, accumulating clinical and basic data derived from depression patients and inbred model mice have demonstrated that depression is associated with multiple levels of abnormalities of brain areas engaged in emotional and cognitive processes (e.g., dorsolateral prefrontal cortex (DLPFC) and hippocampus), including aberrant structure and function as well as neuronal atrophy and synaptic loss^14–17^. The observation that some antidepressants likely exert effects via blocking or reversing these aberrations further confirmed that such phenotypes might play pivotal roles in depression^18,19^. Therefore, translating genetic findings of depression may provide valuable insights into its pathological mechanisms and even facilitate therapeutic development, however, such translational approach requires thorough integrations of the data obtained from multiple perspectives including genomics, neuroscience, pharmacology, and biochemical^20^.

In the present study, with an aim to identify risk genes and relevant mechanisms of depression, we have employed summary data-based Mendelian randomization (SMR)^21,22^ and Bayesian integrative risk gene selector (iRIGS)^23^ to integrate omics data from GWAS, DLPFC eQTL, and genome-scale chromosome conformation capture (Hi-C). Multiple depression risk genes are identified after combined investigation of integrative results, among which the *netrin 1 receptor* (*DCC*) gene is prioritized as a high-confidence candidate. Risk alleles correlated with brain *DCC* mRNA levels show robust link with the onset of depression. We also find that *DCC* associating variants significantly predict depression relevant biological phenotypes, suggesting participation of this gene in the biological processes of depression pathogenesis. These results further confirm the previous functional analyses of *DCC* supporting the hypothesis that it is an authentic and important risk gene for depression.

## Methods and materials

All the protocols and methods used in this study were approved by the institutional review board of the Kunming Institute of Zoology, Chinese Academy of Sciences.

### Depression GWAS data

#### European GWAS data

Depression GWAS data was retrieved from that Howard et al. meta-analyses of UK Biobank, PGC2, and 23andMe GWAS datasets (a total of 246,363 cases and 561,190 controls)^4^. There were originally 102 independent loci identified as showing genome-wide significant associations with depression in Europeans^4^. Due to the restrictions on data share policy of 23andMe sample, the authors publicly deposited the genome-wide statistics combining UK Biobank and PGC2 GWAS datasets (170,756 cases and 329,443 controls) (https://datashare.is.ed.ac.uk/handle/10283/3203), which was utilized for the current analyses. Detailed information of sample characteristics, genotyping method, and statistical analyses of each GWAS dataset can be found in the original studies^4^.

#### Chinese GWAS data

Data of the GWAS of major depression in Han Chinese conducted by the CONVERGE Consortium was collected for the current study^24^. A total of 5303 patients and 5337 non-psychiatric controls after quality control were included. Data was accessed via the public sharing portal at 10.6084/m9.figshare.3840696. Details of the samples, genotyping method and statistical analyses can be found in the initial report^24^.

### SMR integrative analyses

Mendelian randomization (MR) analyses utilize a genetic variation as the instrumental variable to examine causative effects of defined exposure variables (e.g., gene expression) on an outcome (e.g., illness)^25^. It is thus plausible to use MR analysis to identify the risk or even causal genes of complex illnesses through integrating the eQTL data. However, one potential handicap of MR analyses is that this method requires a large cohort of individuals with simultaneously available data on their phenotype, genotype and gene expression profiles, which is usually difficult to recruit. As a possible solution, Zhu et al. have developed a novel alternative method called summary data-based Mendelian randomization (SMR), which requires summary level statistical data from independent GWAS and eQTL datasets for the integration and prioritization of genes whose expression levels are relevant to the illness due to pleiotropic effects^21^. Based on SMR, the authors further developed a multi-SNP-based SMR test (--smr-multi) that considers multiple SNPs at a cis-eQTL locus in the SMR test^22^, and this method is applied in this study. We respectively integrated the brain eQTL datasets from BrainSeq Phase 2^26^, CommonMind^27^, and PsychENCODE^28^ with the European depression GWAS^4^ to perform the SMR analyses. Details of the data and relevant procedure conducted are discussed below.

The BrainSeq Phase 2 is a RiboZero RNA-seq eQTL database of human brain tissues^26^. Data obtained from the DLPFC tissues of 397 individuals older than 13 were included, and gene-level expression eQTL was calculated based on the formula: log_2_(RPKM+1) ~SNP + diagnosis + sex + SNP PCs + expression PCs. The authors identified 1,577,964 eQTL associations at a false discovery rate (FDR) ≤ 1% between 945,693 genetic variants and 13,510 genes, and the eQTL summary data were downloaded from https://s3.us-east-2.amazonaws.com/libd-brainseq2/SupplementaryTable15_eQTL.tar.gz, and then transformed into SMR binary (BESD) file using SMR (version 1.02) for subsequent analyses^21^.

The CommonMind dataset contains polyA^+^ RNA-seq eQTL data of DLPFC tissues collected from 467 European donors (age > 17 years old)^27^. The mRNA levels of genes were normalized using log (CPM) (read counts per million per reads) and adjusted for diagnosis, sex, institution, AOD, PMI, RIN, RIN2, clustered LIB, and 20 SVs. The expression levels were then quantified using a linear eQTL analysis on the imputed genotype dosages based on the formula: adjusted gene expression ~SNP + 5 ancestry vectors + diagnosis. This dataset identified 3,725,946 significant cis-eQTL at a FDR ≤ 5% between 16,089 genes and 2,072,425 SNPs. We downloaded the eQTL summary data from Synapse (https://www.synapse.org/#!Synapse:syn5652278) and transformed them into BESD file using SMR (version 1.02) for the integrative analyses^21^.

A recent study provided the PsychENCODE eQTL summary data at http://resource.psychencode.org/^28^. Briefly, the authors calculated the eQTL association results after correcting for 100 hidden covariate (HCP) factors^28^, and the data was generated based on 1387 individuals recruited by the research projects of CommonMind, CommonMind-HBCC, BrainGVEX, LIBD, and BipSeq etc.^29^. Therefore, although duplicative individuals exist between PsychENCODE and CommonMind and BrainSeq datasets, the PsychENCODE is a larger eQTL dataset with a stronger detection power. The PsychENCODE eQTL summary data in BESD format was directly downloaded from http://resource.psychencode.org/^28^.

For SMR analysis, firstly, these eQTL summary statistics were transformed to the binary format files named BESD, as the key input files of SMR program. In addition, the other major input file was the genome-wide statistics of depression GWAS. SNPs and genes in the major histocompatibility complex (MHC) extended region were removed before SMR analyses due to the complexity of this genomic region (chr6:26M-34M, hg19). The threshold of eQTL *p*-value in the SMR analysis was set to be 1.00 × 10^−5^ (--peqtl-smr 0.00001) and the default values of other parameters were used. The genes with *p*-value less than 5.00 × 10^−4^ of multi-SNP-based SMR test and passed the HEIDI test (*P*_HEIDI_ > 0.005) were considered as susceptibility genes, whose mRNA expression alterations associated with risk SNPs of depression.

### Integrative risk gene selector (iRIGS) analyses

The growing knowledge of the importance of physical interactions between distal regulatory elements (DREs) and target promoters has promoted recent development of multiple technologies (e.g., Hi-C) capable of detecting such interactions^30,31^. Through integrating results of studies applying these approaches, Wang et al.^23^ developed a Bayesian framework, named iRIGS, to probabilistically rank high-confidence risk genes at each GWAS locus of schizophrenia. Following their method, we herein focused on the 102 independent lead risk SNPs from previous European depression GWAS^4^, and used the iRIGS analyses to estimate whether these index SNPs interacted with promoters of particular genes based on the omics data of short-range and long-range enhancer-promoter links. These omics data were derived from four sources in three independent published studies as previously described^23^. The first study is the Hi-C analyses of the cortical/subcortical plate and the germinal zone of human cerebral cortex conducted by Won et al.^31^. This study reported 221,069 and 228,323 DRE-promoter links in the cortical/subcortical plate and the germinal zone, respectively. The second study is a capture Hi-C analysis of the cell line GM12878, a human Epstein-Barr virus (EBV)-transformed lymphoblastoid cell line^30^ and identified 1,618,000 DRE-promoter links. The third study is conducted by the FANTOM5 project to infer the enhancer-promoter links across multiple human tissues, and eventually obtained 66,899 enhancer-promoter links^32^. The data of these studies were placed at https://www.vumc.org/cgg/irigs in a ready-to-use format for iRIGS analyses^23^. The R code of iRIGS were run using the default pipeline. The Bayesian posterior probability of observing the link between a SNP and a particular gene higher than 0.8 was considered to be strong, in which case the gene is a potential risk gene regulated by the SNP.

### Gene-wise MAGMA analyses

The gene-wise *p*-values were respectively calculated using MAGMA (v1.07b)^33^ based on the depression GWAS statistics from Europeans and Han Chinese^4,24^. MAGMA applies Brown’s method to combine SNP p-values which will consider LD, and the window size of each gene is defined as the region spanning 35-kb upstream and 10-kb downstream of this gene as previously described^3^. The *snp-wise* = *mean* gene analysis model was used in the present study, which tests the mean SNP association for each gene. For the LD reference, we utilized European-ancestry and Han Chinese-ancestry individuals from the 1000 Genomes Project (Phase 3)^34^.

### LD analyses

The Haploview program (version 4.1)^35^ was utilized to estimate LD between paired SNPs using the *r*^2^ algorithm, and to determine the haplotype blocks based on the SNP data from the 1000 Genomes Project^34^. The regional association results are plotted using LocusZoom (http://locuszoom.sph.umich.edu/locuszoom/)^36^.

### GWAS of depressive symptoms, neuroticism, life satisfaction, positive affect, and well-being spectrum

Phenotypes such as neuroticism, life satisfaction, and positive affect are generally believed to be associated with depression. Specifically, neuroticism refers to a personality trait characterized by significantly instable mood in response to stress-inducing events^37^, and is presumed to be a risk factor for depression^38^. Phenotypes of the well-being spectrum, including depressive symptoms, neuroticism, life satisfaction, and positive affect, have all been found genetically correlated with depression^38^. We therefore collected GWAS resources of these biological phenotypes from a recent study^39^, in which the authors measured the well-being spectrum using survey questions on depressive symptoms, neuroticism, life satisfaction, and positive affect, and applied multivariate genome-wide-association meta-analysis (GWAMA) on univariate GWAMAs of depressive symptoms (*N* = 1,295,946), neuroticism (*N* = 582,989), life satisfaction (*N* = 80,852), and positive affect (*N* = 410,603), as well as the combinatory well-being spectrum (*N* = 2,370,390).

### GWAS of cognitive performance and intelligence

The data of cognitive performance were retrieved from a recent GWAS of 257,828 individuals^40^. In their study, the authors conducted a meta-analysis of the general cognitive ability GWAS by the COGENT consortium^41^ and additional results of the recent cognitive performance analyses in UK Biobank^42^. Meanwhile, the authors also conducted GWAS analysis of educational attainment (*N* = 766,345)^40^, a proxy phenotype of cognitive abilities that is believed to also reflect some personality traits. In addition, we also retrieved the results of intelligence from a GWAS of 269,867 individuals into the current study^43^. The authors calculated and applied a common latent *g* factor underlying multiple dimensions of cognitive functioning during statistical analyses given the distinct approaches of intelligence measurement in each sample.

### GWAS of brain imaging phenotypes

Statistics of brain imaging analyses were extracted from a recent GWAS of 3144 functional and structural brain imaging phenotypes (e.g., hippocampal volume, putamen volume, task functional MRI median BOLD faces) in 8,428 subjects by the UK Biobank (accessed at http://big.stats.ox.ac.uk/)^44^. In this website, GWAS results of other related phenotypes were also deposited (i.e., GWAS of a total of 3999 UK Biobank brain imaging phenotypes and other traits). We also collected the independent data from the ENIGMA Consortium GWAS of different subcortical brain volumes (accumbens, amygdala, caudate, hippocampus, pallidum, putamen, and thalamus)^45^. According to the previous study^44^, there was a strong correlation between the UK Biobank and ENIGMA imaging samples, suggesting that methodologies applied in the measurement and statistical analyses of these phenotypes were relatively consistent. In addition, a more recent GWAS has been conducted to meta-analyze imaging data from CHARGE, ENIGMA and UK Biobank, resulting in a total of 38,851 subjects^46^.

### RNA-seq, mRNA correlation, and pathway analyses in human brains

We downloaded the aligned (hg19 as reference genome) RNA-seq data (bam files) of the DLPFC tissue from three independent sample pools (BrainGVEX, CommonMind, and LIBD)^27,29^, and only non-psychiatric controls were utilized to prevent effects of confounders relevant to medical treatment. Based on this criterion, 59 controls from BrainGVEX, 50 controls from CommonMind, and 70 controls from LIBD were included. We applied the same procedures and criteria for the quality control across the three RNA-seq datasets as previously described^47,48^. The counts of aligned reads at the gene level were calculated using featureCounts according to the annotation file of GRCh37p13^49^. We calculated the transcripts per million reads (TPM) of each gene using R program following a previous study^28^, and only kept the protein-coding genes with their average TPM ≥ 1.0 for the following analyses. The TPM of each gene was log2 transformed followed by Pearson analyses to assess their correlations with *DCC*. We used clusterProfiler^50^ to analyze whether the “*DCC*-correlated” genes were significantly enriched in specific molecular pathways and biological processes via Kyoto Encyclopedia of Genes and Genomes (KEGG) and Gene Ontology (GO) annotations based on the integrative database called org.Hs.eg.db, and FDR *q*-value less than 0.1 was set to be reliable. We then performed semantic similarity analyses with GOSemSim^51^ to narrow down those GO terms based on their similarity between each other (*r* > 0.5 was considered highly similar).

## Results

### Integrative analyses of multi-omics data identified high-confidence risk genes for depression in Europeans

**Fig. 1:**
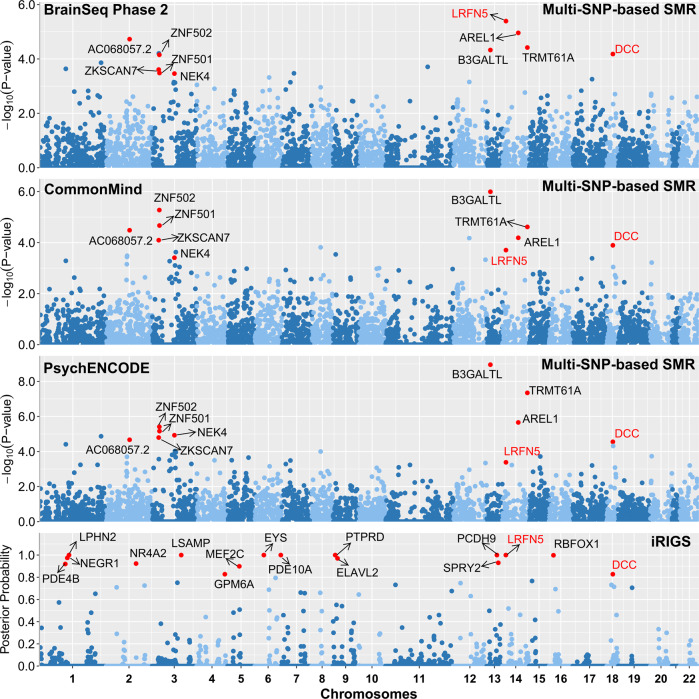
Multi-SNP-based SMR analyses through integrating different DLPFC eQTL datasets (BrainSeq Phase 2, CommonMind, and PsychENCODE), and iRIGS analyses of the risk SNPs form European depression GWAS. Ten genes of *P*_SMR-multi_ less than 5.00 × 10^−4^ in all three eQTL datasets and sixteen genes of posterior probability more than 0.8 in iRIGS were marked in red.

We applied multi-SNP-based SMR method to test the associations between risk SNPs of depression identified in the GWAS study^4^ and mRNA expression based on two independent DLPFC eQTL datasets from BrainSeq Phase 2 (*N* = 397)^26^ and CommonMind (*N* = 467)^27^, respectively. Subsequently, we performed additional analyses to confirm these findings using a larger eQTL dataset from PsychENCODE^29^, which consisted of more individuals (*N* = 1387) that were also partly overlapped with those in CommonMind and BrainSeq. The number of genes included in the SMR analysis were 5502 (BrainSeq Phase 2), 6036 (CommonMind) and 13,567 (PsychENCODE), respectively. The remaining genes were 5,455, 5,981 and 13,372 after ignoring the genes failing to pass the HEIDI test and 3239 genes were overlapped in all datasets. Finally, 16, 18, and 30 susceptibility genes (*P*_SMR-multi_ ≤ 5.00 × 10^−4^) were identified in the three eQTL datasets, respectively. As expected, SMR analyses using this larger eQTL samples replicated most of the risk genes identified in the earlier datasets, and a total of 10 risk genes exhibited statistical significance throughout all the SMR analyses (Fig. 1; Table 1).

**Table 1:** Detailed information about the significant genes in SMR analyses.

CHR	Gene				Multi-SNP-based SMR			iRIGS					MAGMA	
	Name	Start	End	Type	BrainSeq	CommonMind	PsychENCODE	Index SNP	CHR	Position	*P*_depression_	Post_Prob	European	Chinese
2	*AC068057.2*	105363096	105374177	lincRNA	1.87E–05	3.27E–05	2.13E–05	NA	NA	NA	NA	NA	6.33E–06	0.364
3	*ZKSCAN7*	44596685	44624975	protein_coding	2.45E–04	8.02E–05	1.59E–05	rs4346585	3	44736493	7.13E–10	0.061	6.80E–08	0.484
3	*ZNF502*	44754135	44765323	protein_coding	7.02E–05	5.29E–06	3.85E–06	rs4346585	3	44736493	7.13E–10	0.031	4.38E–05	0.394
3	*ZNF501*	44771088	44778575	protein_coding	3.29E–04	2.14E–05	6.84E–06	rs4346585	3	44736493	7.13E–10	0.024	1.78E–04	0.320
3	*NEK4*	52744800	52804965	protein_coding	3.45E–04	3.91E–04	1.17E–05	rs7624336	3	53244151	3.96E–08	0.005	3.44E–05	0.436
13	*B3GALTL*	31774073	31906413	protein_coding	4.68E–05	1.01E–06	1.11E–09	rs1409379	13	31907741	1.67E–09	0.026	7.21E–13	0.777
**14**	***LRFN5***	**42076773**	**42373752**	**protein_coding**	**4.05E**–**06**	**1.96E**–**04**	**4.11E**–**04**	**rs61990288**	**14**	**42074726**	**1.68E**–**13**	**1.000**	**1.01E**–**11**	**0.406**
14	*AREL1*	75120140	75179818	protein_coding	1.10E–05	6.47E–05	2.18E–06	rs1045430	14	75130235	7.31E–13	0.019	1.32E–07	0.572
14	*TRMT61A*	103995521	104003410	protein_coding	3.80E–05	2.42E–05	4.47E–08	rs10149470	14	104017953	3.72E–14	0.002	6.41E–10	0.624
**18**	***DCC***	**49866542**	**51057784**	**protein_coding**	**6.58E**–**05**	**1.27E**–**04**	**2.78E**–**05**	**rs7227069**	**18**	**50731802**	**1.50E**–**11**	**0.828**	**2.85E**–**12**	**3.21E**–**04**

*Post_Prob* posterior probability.

We then conducted iRIGS analyses to identify potential risk genes through evaluating the physical interactions between enhancers and promoters. Briefly, whether the 102 independent lead risk SNPs from previous European depression GWAS were involved in such interactions were examined^4^. SNPs and genes in the MHC extended region were removed before iRIGS analyses, and the enhancer-promoter links between 1229 genes and these depression risk SNPs were then captured. Our results revealed that the posterior probabilities of 16 genes were higher than 0.8, indicating that they are potentially high-confidence depression risk genes under the influences of the risk SNPs (Fig. 1 and Table S1).

Through the SMR and iRIGS analyses, two genes were identified in both approaches (marked in bold and underlined in Table 1). The first gene, *LRFN5* at 14q23.1, showed significant associations in SMR (*P*_SMR-multi_ = 4.05 × 10^−6^ in BrainSeq Phase 2, *P*_SMR-multi_ = 1.96 × 10^−4^ in CommonMind, *P*_SMR-multi_ = 4.11 × 10^−4^ in PsychENCODE) and iRIGS analyses (posterior probability = 1.000). This gene encodes a protein of leucine rich repeat and fibronectin type III domain containing 5, and previous studies implicated its crucial roles in synapse formation and differentiation^52–56^.

Another high-confidence risk gene is *DCC* at 18q21.3 which encodes a transmembrane receptor for the protein netrin-1. *DCC* exhibited significant association signals in both SMR (*P*_SMR-multi_ = 6.58 × 10^−5^ in BrainSeq Phase 2, *P*_SMR-multi_ = 1.27 × 10^−4^ in CommonMind, *P*_SMR-multi_ = 2.78 × 10^−5^ in PsychENCODE) and iRIGS analyses (posterior probability = 0.828). *DCC* has been extensively described in a recent review regarding its potential roles in the central nervous systems and psychiatric disorders^57^, in brief, this gene is highly expressed in dendritic spines of pyramidal neurons, and exerts pivotal regulatory effects on synaptic function and plasticity in adult brain^58^. The Dcc protein is known to be a cell adhesion molecule that mediates the effects of netrin-1 on axon outgrowth^59–61^, and deficits of *Dcc* in the adult forebrain neurons results in aberrant long-term potentiation (LTP), long-term depression (LTD) and dendritic spine morphology, as well as impaired spatial and recognition^57,58^. Intriguingly, recent studies have shown that *DCC* mRNA levels in the prefrontal cortex of antidepressant-free depression patients who committed suicide were significantly elevated comparing with control subjects (i.e.*, DCC* mRNA levels were 48% higher in cases compared to control subjects in the first cohort by Manitt et al. (*p* < 0.026)^62^, and were ~40% higher in cases in comparison with controls in the second independent cohort by Torres-Berrío et al. (*p* = 0.02)^63^). Moreover, in a blood transcriptome analysis, *DCC* was again significantly upregulated in depression patients compared to controls in two independent samples (GSK-HiTDIP cohort, fold change = 1.085, *p* = 0.0231; Janssen-BRC cohort, fold change = 1.137, *p* = 0.000655)^64^. In addition, in the mPFC of mice exhibiting chronic social defeat stress (CSDS) exposure-induced depressive-like symptoms, expression level of *Dcc* is also increased^63^; on the other hand, decreased *Dcc* expression in the mouse PFC pyramidal neurons produces resilience against stress-induced depression-like phenotypes^63^.

### Independent replications across populations further confirmed the association of DCC with depression

Our analyses so far suggest pivotal roles of the SNPs spanning *LRFN5* and *DCC* in the risk of depression in Europeans^4^. We then performed the gene-level analyses via MAGMA^33^, which primarily consider LD structures. Intriguingly, the significant associations between these two genes with the risk of depression were again observed in Europeans (*P*_MAGMA_ = 1.01 × 10^−11^ for *LRFN5*, *P*_MAGMA_ = 2.85 × 10^−12^ for *DCC*, Table 1). Given the emerging evidence supporting the notion that vital genetic markers for psychiatric disorders are normally associated with the disease across different ethnic populations^65^ (e.g., psychiatric risk loci in *ZNF804A*, *FADS1*, and *VRK2* show significant associations in both Europeans and East Asians^66–71^), we then examined whether *LRFN5* and *DCC* were also associated with depression in Han Chinese subjects through MAGMA gene-level analyses using a published Han Chinese GWAS dataset (5303 cases and 5337 controls)^24^. Notably, *DCC* was associated with depression as well in Han Chinese despite the lower level of statistical significance compared with that in Europeans (*P*_MAGMA_ = 3.21 × 10^−4^, Table 1), but *LRFN5* was not associated with depression in Han Chinese (*P*_MAGMA_ = 0.406, Table 1). A detailed examination found that none of the SNPs spanning *LRFN5* were significantly associated with depression Han Chinese (all *p* > 0.01, Fig. S1).

### Identification of SNPs in DCC showing significant associations with depression in both populations

**Fig. 2:**
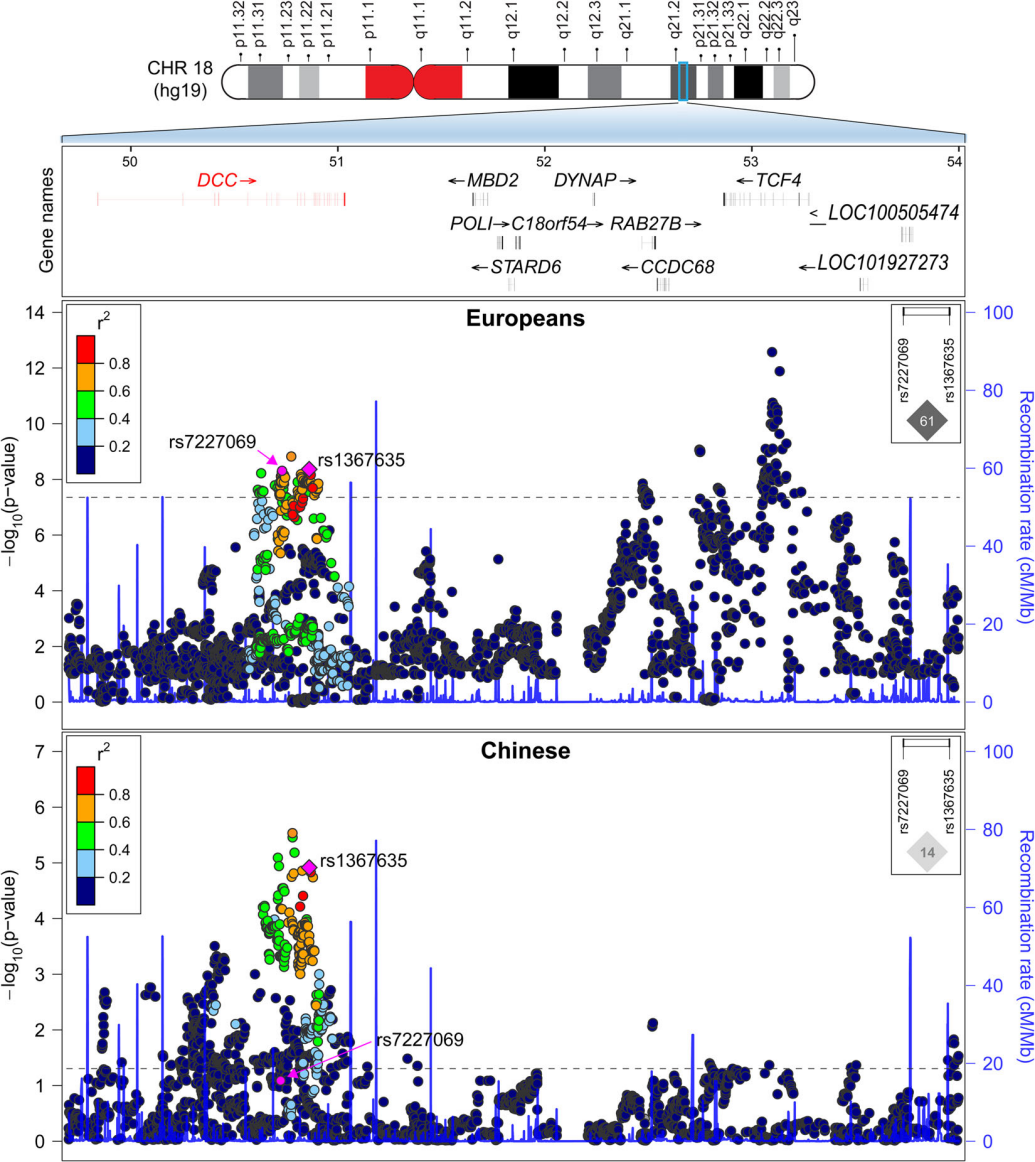
Genetic associations of SNPs spanning *DCC* region with depression in Europeans and Han Chinese populations. A physical map of the region is given and depicts known genes within the region, and the LD is defined based on the SNP rs1367635. The LD between rs7227069 and rs1367635 in both populations are also shown.

It is generally acknowledged that potential causal variants usually exhibit consistent associations with the illnesses across populations, whereas the associations for other linked variants may be significantly affected by the different LD structures between populations^72,73^. To further explore the genetic architecture of complex illnesses, cross-population replication analysis is usually necessary. In this study, our detailed examinations found that although multiple SNPs spanning *DCC* showed genome-wide significant associations with depression in Europeans, their associations with the illness in Han Chinese were distinct possibly due to variations in LD (Fig. 2). For example, rs7227069 showed genome-wide significant association with depression in 170,756 European cases and 329,443 matching controls collected by UK Biobank and PGC2 (rs7227069, *P*_depression_ = 4.64 × 10^−9^, odds ratio (OR) = 1.026 for A-allele, Fig. 2), and when the 23andMe GWAS dataset was also added, the association between rs7227069 and depression was strengthened in the new sample pool of 246,363 cases and 561,190 controls (*P*_depression_ = 1.50 × 10^−11^, OR = 1.024 for A-allele)^4^. The risk association was further confirmed in an independent cohort of 414,055 cases and 892,299 controls (*P*_depression_ = 8.89 × 10^−19^, OR = 1.025 for A-allele)^4^. While in Han Chinese subjects, rs7227069 was only marginally associated with depression (*P*_depression_ = 0.071, OR = 1.127 for A-allele in 5303 cases and 5337 controls, Fig. 2)^24^. By comparing the allele distributions of rs7227069 in Europeans and Chinese, we found that the frequency of A-allele at rs7227069 was 0.425 in Europeans, but it is not a common SNP in Han Chinese (frequency of A-allele is 0.038), which likely contributed to the different levels of association significance of this SNP with depression between populations.

Notably, we found another SNP rs1367635 in *DCC*, which was genome-wide significantly associated with depression in Europeans (*P*_depression_ = 4.35 × 10^−9^, OR = 1.026 for C-allele in 170,756 cases and 329,443 controls, Fig. 2), was also highly associated with this illness in Han Chinese (*P*_depression_ = 1.21 × 10^−5^, OR = 1.173 for C-allele in 5303 cases and 5337 controls, Fig. 2). Rs1367635 and rs7227069 lie more than 129.0-kb apart in different introns of the *DCC* gene. They are in moderate LD in Europeans (*r*^2^ = 0.61), while low LD in Han Chinese (*r*^2^ = 0.14), highlighting the genetic heterogeneity in this locus between populations. Rs1367635 is a common SNP in both Europeans and Han Chinese despite different allelic frequencies between populations (C-allele, 0.501 in Europeans versus 0.183 in Han Chinese), and rs1367635 exhibited a stronger association signal with depression in Han Chinese than rs7227069 probably due to its higher minor allele frequency (MAF) in this population. Although the relatively smaller sample size of the current depression GWAS of Han Chinese (5303 cases and 5337 controls)^24^ has limited the level of statistical significance of the association signal of rs1367635 with depression, the effect size of rs1367635 on the risk of depression is comparable to other risk loci identified in genome-wide analyses^3^. We also performed a power analysis of rs1367635 according to its effect size on the risk of depression in Han Chinese and its allelic frequency in this population. This analysis estimated that at least 39,628 cases and controls in total were needed to reach the 80% power of detecting genome-wide significant association. Further analyses of the association between rs1367635 and depression in a large enough cohort will likely reveal a genome-wide level significant association.

### The depression risk alleles indicated higher DCC mRNA level in DLPFC

**Fig. 3:**
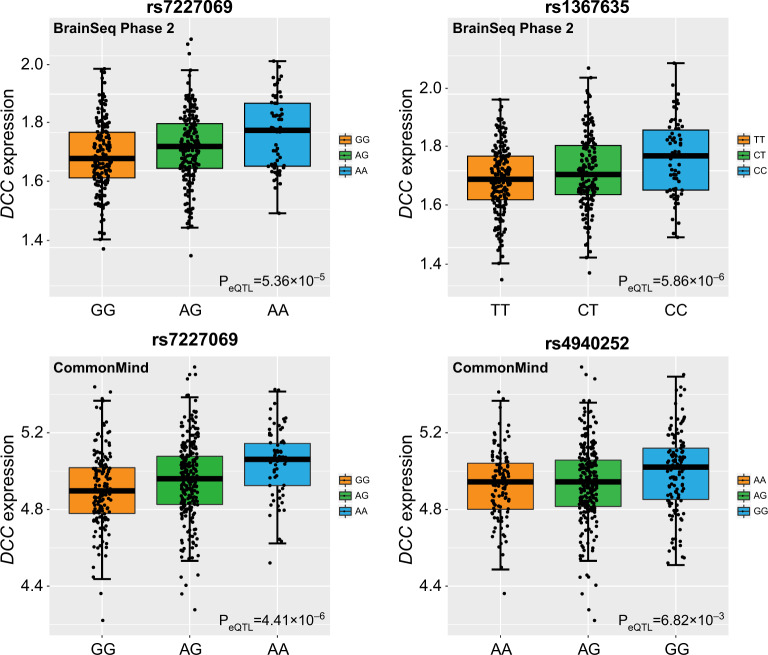
Expression quantitative trait loci (eQTL) analyses of rs7227069 and rs1367635/rs4940252 with *DCC* mRNA expression in BrainSeq Phase 2 and CommonMind datasets. In the CommonMind eQTL dataset, rs1367635 was not directly genotyped or imputed, and we therefore used its high LD SNP rs4940252 as a proxy readout (*r*^2^ = 0.880 between rs1367635-T/C and rs4940252-A/G in Europeans).

We examined whether the depression risk SNPs (e.g., rs7227069 and rs1367635) spanning *DCC* were also associated with *DCC* mRNA expression in DLPFC. Rs7227069 showed significant association with mRNA expression of *DCC* in BrainSeq Phase 2 eQTL dataset, which included the RiboZero RNA-seq results of DLPFC tissues from 397 individuals (*P*_eQTL_ = 5.36 × 10^−5^, Fig. 3); in the CommonMind polyA^+^ RNA-seq eQTL dataset of 467 individuals, rs7227069 again exhibited significant association with *DCC* mRNA levels (*P*_eQTL_ = 4.41 × 10^−6^, Fig. 3); while rs7227069 was not covered in the PsychENCODE dataset of 1,387 subjects, its complete LD SNP rs8086812 (*r*^2^ = 1.000 in Europeans) was significantly associated with *DCC* mRNA expression (*P*_eQTL_ = 7.05 × 10^−15^, eQTL plot in PsychENCODE dataset was not available since we do not have access to individual-level genotype and expression data).

Rs1367635 also showed significant association with mRNA expression of *DCC* in BrainSeq Phase 2 and PsychENCODE eQTL datasets (*P*_eQTL_ = 5.86 × 10^−6^ in BrainSeq Phase 2, *P*_eQTL_ = 1.76 × 10^−8^ in PsychENCODE; Fig. 3). In the CommonMind eQTL dataset, rs1367635 was not genotyped or imputed, and we therefore used its high LD SNP rs4940252 as a proxy readout (*r*^2^ = 0.880 in Europeans). Again, rs4940252 was significantly associated with *DCC* mRNA expression in the CommonMind dataset (*P*_eQTL_ = 6.82 × 10^−3^, Fig. 3). Therefore, the depression risk alleles at rs7227069 and rs1367635 consistently indicated higher *DCC* expression in the three selected eQTL datasets. This result is consistent with the published diagnostic analyses of *DCC* mRNA level in humans and rodent models^62,63^, suggesting that increased expression of *DCC* in the brain likely serves as a risk factor of depression.

We also explored the expression pattern of *DCC* in diverse human tissues using GTEx (Genotype-Tissue Expression project; https://www.gtexportal.org/) dataset^74^, and found that *DCC* was preferably expressed in frontal cortex, caudate, nucleus accumbens, putamen and hippocampus compared with most other peripheral tissues (only except testis) (Fig. S2). Further spatio-temporal expression pattern analyses using data of the developing prefrontal cortex from the BrainCloud dataset (http://braincloud.jhmi.edu/)^75^ showed that the mRNA expression levels of *DCC* were higher at early developmental stages (i.e., fetal stages) compared with the later childhood and adulthood stages (Fig. S3). We then performed the same analyses in data of multiple human brain tissues (e.g., dorsolateral prefrontal cortex, hippocampus, cerebellar cortex) from the BrainSpan dataset (http://www.brainspan.org/static/home)^76^. Again, we observed that the mRNA levels of *DCC* were higher in prenatal brain tissues than in postnatal brain tissues. Notably, the expression of *DCC* was the highest during early or mid prenatal stages (9–13 post-conceptional weeks (pcw)) (Fig. S4), at which the developing brain starts to undergo striking changes such as the formation of gyri and sulci^77^. This result supports the putative molecular origins of psychiatric disorders arise from the early developmental events^78,79^. Given the high expression levels of *DCC* in prenatal brains, using eQTL datasets including samples from only postnatal donors (e.g., CMC samples > 17 years old; BrainSeq2 samples > 13 years old) may not fully reveal eQTL association signals involved in psychiatric disorders. We therefore also explored the eQTL associations of risk SNPs with *DCC* mRNA expression in fetal brains using recently published data in European populations^80,81^. Briefly, data of 120 prenatal human brain samples (second trimester of gestation) included in the Human Developmental Biology Resource (http://www.hdbr.org)^80^ was retrieved and analyzed. The risk A-allele at rs7227069 again predicted higher *DCC* expression (*p* = 0.01) (Table S2). There were 427 additional SNPs significantly associated with the expression of *DCC* (*p* < 0.05), and many of these SNPs also showed genome-wide significant associations with depression (Table S2). However, rs1367635 was not associated with *DCC* expression in this dataset. In another 201 prenatal human brain samples (mid-gestational human brains) from the UCLA Gene and Cell Therapy core^81^, neither rs7227069 nor rs1367635 showed significant association with *DCC* expression, but there were other SNPs significantly associated with both *DCC* expression and risk of depression (Table S3). Overall, these results support the hypothesis that certain genetic variations influencing *DCC* expression in human brains also affect risk of depression.

### DCC was associated with well-being spectrum, cognitive function, and putamen structure

Given that several biological and psychological phenotypes such as mood instability, depressive symptoms and aberrant cognitive functions have been common observed in depression patients^38,82^, we hypothesized that these phenotypes were also associated with the depression risk alleles at *DCC*. We utilized the published GWAS resources and found that *DCC* SNPs showed significant associations with depressive symptoms (rs7227069, *p* = 6.18 × 10^−16^; rs1367635, *p* = 8.57 × 10^−15^), and were also highly associated with neuroticism (rs7227069, *p* = 4.73 × 10^−16^; rs1367635, *p* = 8.23 × 10^−15^), well-being spectrum (rs7227069, *p* = 2.12 × 10^−16^; rs1367635, *p* = 8.58 × 10^−15^), life satisfaction (rs7227069, *p* = 4.92 × 10^−11^; rs1367635, *p* = 1.91 × 10^−11^) and positive affect (rs7227069, *p* = 4.36 × 10^−15^; rs1367635, *p* = 5.25 × 10^−13^)^39^, among which the depression risk allele carriers tended to show increased vulnerable personality traits compared with the protective allele carriers. We also found that rs7227069 and rs1367635 showed strong associations with cognitive performance (rs7227069, *p* = 2.15 × 10^−6^; rs1367635, *p* = 1.48 × 10^−6^)^40^, educational attainment (rs7227069, *p* = 5.95 × 10^−13^; rs1367635, *p* = 1.10 × 10^−9^)^40^, and intelligence (rs7227069, *p* = 1.34 × 10^−6^; rs1367635, *p* = 2.58 × 10^−8^)^43^, while carriers of the depression risk alleles tended to exhibit worse cognitive abilities compared with the non-risk allele carriers.

We then took one step further to delve into potential neural mechanisms underlying this risk gene. In a phewas analysis including GWAS of 3999 UK Biobank brain imaging phenotypes and other traits (http://big.stats.ox.ac.uk/), rs7227069 and rs1367635 were again primarily associated with depression related traits (such as mood swings and frequency of depressed mood in last 2 weeks, Fig. S5)^44^, further highlighting the involvement of *DCC* in this illness. More intriguingly, in this explorative analysis, we found that *DCC* was suggestively genome-wide significantly associated with putamen volume in the UK Biobank dataset (*N* = 8428 individuals; rs7227069, *p* = 9.90 × 10^−7^; rs1367635, *p* = 8.90 × 10^−6^; Fig. S5)^44^. We then tried to replicate this observation in an independent GWAS of brain imaging phenotypes from ENIGMA consortium^45^, and again observed suggestive genome-wide association between *DCC* and putamen volume in the same direction of allelic effects (*N* = 13,145 subjects; rs7227069, *p* = 3.32 × 10^−7^; rs1367635, *p* = 6.64 × 10^−7^)^45^. The imaging samples from UK Biobank and ENIGMA were totally independent. More intriguingly, in a latest meta-analysis of imaging data from CHARGE, ENIGMA and UK Biobank (*N* = 38,851), both SNPs showed genome-wide significant associations with putamen volume (rs7227069, *p* = 4.64 × 10^−10^; rs1367635, *p* = 8.72 × 10^−10^)^46^.

### DCC participated in the pathways and biological processes relevant to depression pathogenesis

**Fig. 4:**
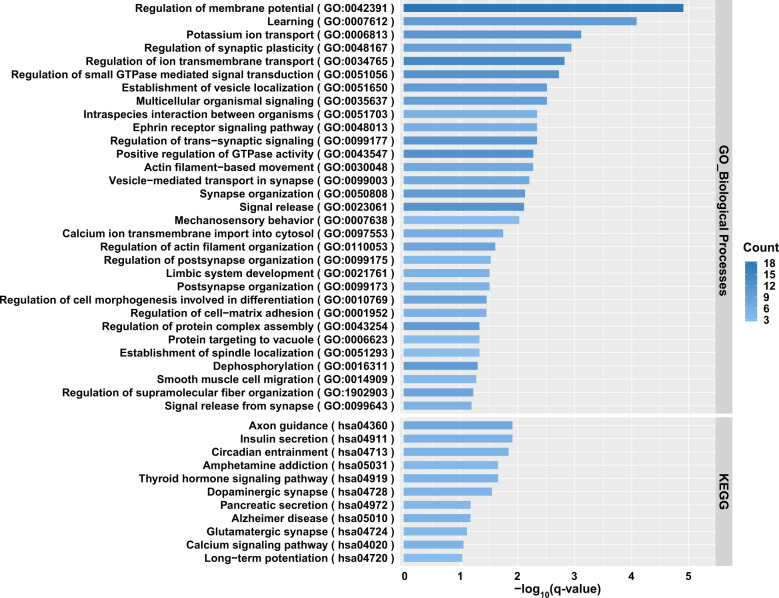
Kyoto Encyclopedia of Genes and Genomes (KEGG) pathway and Gene Ontology (GO) biological processes enrichment analyses of *DCC* correlated genes in the human DLPFC brain tissues.

Previous neurological studies using cellular and molecular technologies have suggested that *DCC* might be involved in the regulation of synaptic plasticity and relevant brain functions^58,59,83,84^. Given the importance of these biological processes in cognition and emotion, we sought to further explore the possibility that *DCC* participates in depression via affecting these processes using population-level expression data. We hypothesized that genes significantly correlated with the mRNA expression of *DCC* should belong to the molecular pathways and biological processes in which *DCC* was involved. To test this hypothesis, the global mRNA expression data in three independent human brain RNA-seq datasets (i.e., BrainGVEX, CommonMind, and LIBD)^27,29^ was retrieved and the Pearson analysis was conducted. We ranked the top 800 genes (~top 5% among all protein-coding genes) in each dataset according to the corresponding significance levels of correlations between their expression and *DCC* mRNA levels. These top 800 genes from all three datasets then underwent an overlapping analysis, which yielded 145 genes consistently showing high correlations with *DCC* expression. There genes were significantly enriched in the pathways and biological processes related to synaptic plasticity, axon guidance, circadian entrainment, learning, and long-term potentiation (Fig. 4). Therefore, our analyses using population-level expression data also supports the putative roles of *DCC* in synapses and brain functions, providing hints for the molecular mechanisms explaining the participation of *DCC* in depression pathogenesis.

## Discussion

Great efforts have been spent to elucidate genetic and biological basis of depression in the past few decades^85,86^, however, the high prevalence, great phenotypic heterogeneity, and limited penetrance of known genetic risk markers of this illness have together significantly hampered success in this field. Nevertheless, multiple breakthroughs have been made through genetic studies of depression since the application of recent large GWAS, and integration of the massive data to dissect mechanisms underlying depression pathogenesis has been widely called on. In the present study, we utilize data of depression GWAS, and conduct the genome-wide integrative analyses through combining multiple brain eQTL and Hi-C datasets, followed by independent replications across populations and explorative analyses of relevant biological phenotypes. Through this stepwise analysis, we find that the gene *DCC* confers risk of depression in both Europeans and Han Chinese. Besides, the risk alleles predict higher *DCC* mRNA expression in the DLPFC, which is also proven to affect depression-relevant personality traits, cognitive function and putamen volumes in independent samples. The current study reveals that the depression risk alleles indicate larger putamen volume. Putamen is a brain region known to influence motor behaviors and learning abilities, and has also been reported to involve in the “hate circuit”^87^ that was lately proven vital in depression^88^. Although previous studies did not identify significant putamen volume differences between depression patients and healthy controls^89^, its involvement in this illness is appealing for further analyses with larger samples of different ethnic backgrounds to gain insights into the sophisticated mechanisms of depression.

Notably, a recent meta-analysis of 232,964 cases and 494,162 controls across eight psychiatric illnesses (anorexia nervosa, attention-deficit/hyperactivity disorder, autism spectrum disorder, bipolar disorder, major depression, obsessive-compulsive disorder, schizophrenia, and Tourette syndrome) detected 109 loci associated with at least two of them. Intriguingly, the *DCC* genomic region featured the most pleiotropic association (*P*_meta_ = 4.26 × 10^−12^) in this meta-analysis, exhibited significant associations with all eight diseases^90^. Additionally, previous studies have also highlighted *DCC* in mood instability and the risk of depression^57,91–93^ as well as schizophrenia^94^, and multiple in vitro and murine analyses have proven its potential impact on synaptic function. For example, the protein netrin-1 and its receptor (encoded by *Dcc*) are widely expressed in cortical neurons during synapse formation, with significant enrichment at synapses^83^. Another study showing enrichment of *Dcc* in dendritic spines of pyramidal neurons further supports its involvement in synaptic physiology, and selective deletion of *Dcc* leads to loss of LTP, intact LTD, reduced spine length, and impaired spatial and recognition memory in mice^58,95^. The *Dcc* deficient/haploinsufficient mice study found that alterations in dcc expression resulted in selective alternations in dopaminergic function (e.g., exaggerated mPFC dopamine concentrations), differences in dopaminergic related behaviors during adulthood, and blunted behavioral responses to amphetamine^96–100^. From the neurodevelopmental perspective, *Dcc* likely controls the growth of dopamine axon targeting in adolescence, and thereby affects the development and function of prefrontal cortex^84,101,102^, resulting in aberrant cognitive processes found in depression. In agreement with this hypothesis, a previous study found that the *DCC* haploinsufficient adult Quebecers showed similar phenotypic features with adult *Dcc* haploinsufficient mice^103^.

Earlier studies using postmortem brain tissues have revealed neuronal loss, reduced synapse density and abnormal expression of synaptic markers in the DLPFC of depression patients^14,15^, and in vitro and in vivo studies also found that risk factors of depression (e.g., stress and genetic effects) usually result in disruption of synaptic morphology and function as well as brain circuits that are essential for mood regulation and cognition^104,105^, and thereby eventually lead to the onset of depression. Moreover, both established antidepressants used in clinical settings (*e.g*., serotonin-reuptake inhibitors) and molecules recently found to alleviate depressive symptoms (e.g., ketamine) confer protective effects on synaptic deficits related to depression^18,19^. Therefore, it is widely accepted that synaptic dysfunctions play determinant roles in the pathogenesis of depression^16,19^. Recent depression GWAS have also supported this contention, as genes involved in synaptic structure and neurotransmission related pathways have been repeatedly highlighted^4^. *DCC* is also an example of such genes, and further functional studies are urgently needed to gain mechanistic insights into whether and how it affects synaptic functions, brain circuits and behaviors in a disease-specific manner. Whether this gene or its proximate signaling pathway might serve as potential therapeutic targets should also be analyzed.

From the Fig. 2, we can see that sequence variations spanning *TCF4* gene were also genome-wide significantly associated with depression in European populations^4^, and the magnitude is even stronger than those spanning *DCC*. However, *TCF4* was not associated with depression in Han Chinese, and the current study therefore did not include this gene in the subsequent analyses. Nevertheless, the potential importance of this gene in depression or other relevant illnesses/phenotypes should not be denied. In fact, *TCF4* has gained considerable attention from researchers due to its significant associations with depression, schizophrenia, cognitive processing^106^, and Pitt-Hopkins Syndrome^107^. In a recent schizophrenia GWAS^108^, numerous SNPs spanning *TCF4* showed genome-wide significance, and the involvement of this gene in synaptic plasticity^109,110^, CNS development^111^ and neuronal activity regulatory network^112,113^ has been defined by functional analyses. We also sought to identify whether the association of *DCC* with depression in Europeans were a reflection of its potential LD with *TCF4*, and examined the distance and LD among SNPs spanning the two genes. We found that *DCC* and *TCF4* were ~2.0 Mb away from each other on the genome, the risk signals of these two genes were independent in Europeans according to the LD structure (Fig. 2). Therefore, SNPs in *DCC* are likely genetic risk markers of depression independent of those in *TCF4*.

Despite the above implications brought by this study, there are limitations to be interpreted. First, our study mainly focuses on variants affecting expression of particular genes, but a number of the genome-wide risk alleles for depression were not associated with expression of any genes in our analyses. While the principal purpose of our study is to prioritize high-confidence risk genes through integrative stepwise analyses, the possibility that the variants not highlighted in eQTL analyses also contribute to the progression of depression should not be denied. In fact, the characteristics of the eQTL datasets utilized in the current study might have “twisted our perceptions” of these variants. For example, the eQTL data we utilized was obtained from DLPFC homogenates rather than from specific types of cells, in the event that some variants and genes exert function in particular types of cells, the current datasets would be inappropriate for detecting such signals. Besides, the individuals in the eQTL datasets were postnatal in a wide range of different ages, making it an ideal source for exploring genes functioning throughout the lifespan but not those participate in depression in a spatio-temporal specific manner. In fact, previous studies have shown that the some psychiatric risk eQTLs are detectable only in a specific stage of life^81,114^. Further comprehensive functional annotations of other depression risk alleles are necessary to gain a better knowledge of the genetic risk architecture of depression. In addition, despite we found that multiple risk SNPs were significantly associated with mRNA expression of *DCC*, all of them were in the intron regions. Although we attempted to make functional predictions of the risk SNPs using HaploReg v4.1^115^ and GWAVA^116^ through assessing whether they reside in the binding regions of H3K4me1, H3K4me3, H3K9ac, H3K27ac, or transcription factors, we were unable to identify any SNPs showing potential regulatory functions. Therefore, a more complicated regulatory mechanism might underlie the association between the risk SNPs and *DCC* mRNA expression. One possibility might be that the risk SNPs reflect a causal structural variation beyond the detection scope of the current GWAS approach. While the importance of *DCC* in depression should be acknowledged, further studies revealing the causal variant in this genomic region are needed.

## Supplementary information

Supplementary Materials
