# Supplementary material for: Further confirmation of netrin 1 receptor (DCC) as a depression risk gene via integrations of multi-omics data
Source: Transl Psychiatry. 2020 Mar 17;10:98. doi: 10.1038/s41398-020-0777-y (PMC7078234; doi:10.1038/s41398-020-0777-y)
Supplement: Supplementary file 1 — Supplementary Materials [file 41398_2020_777_MOESM1_ESM.pdf]

Figure S1. Regional plot of LRFN5 with depression in European and Chinese populations

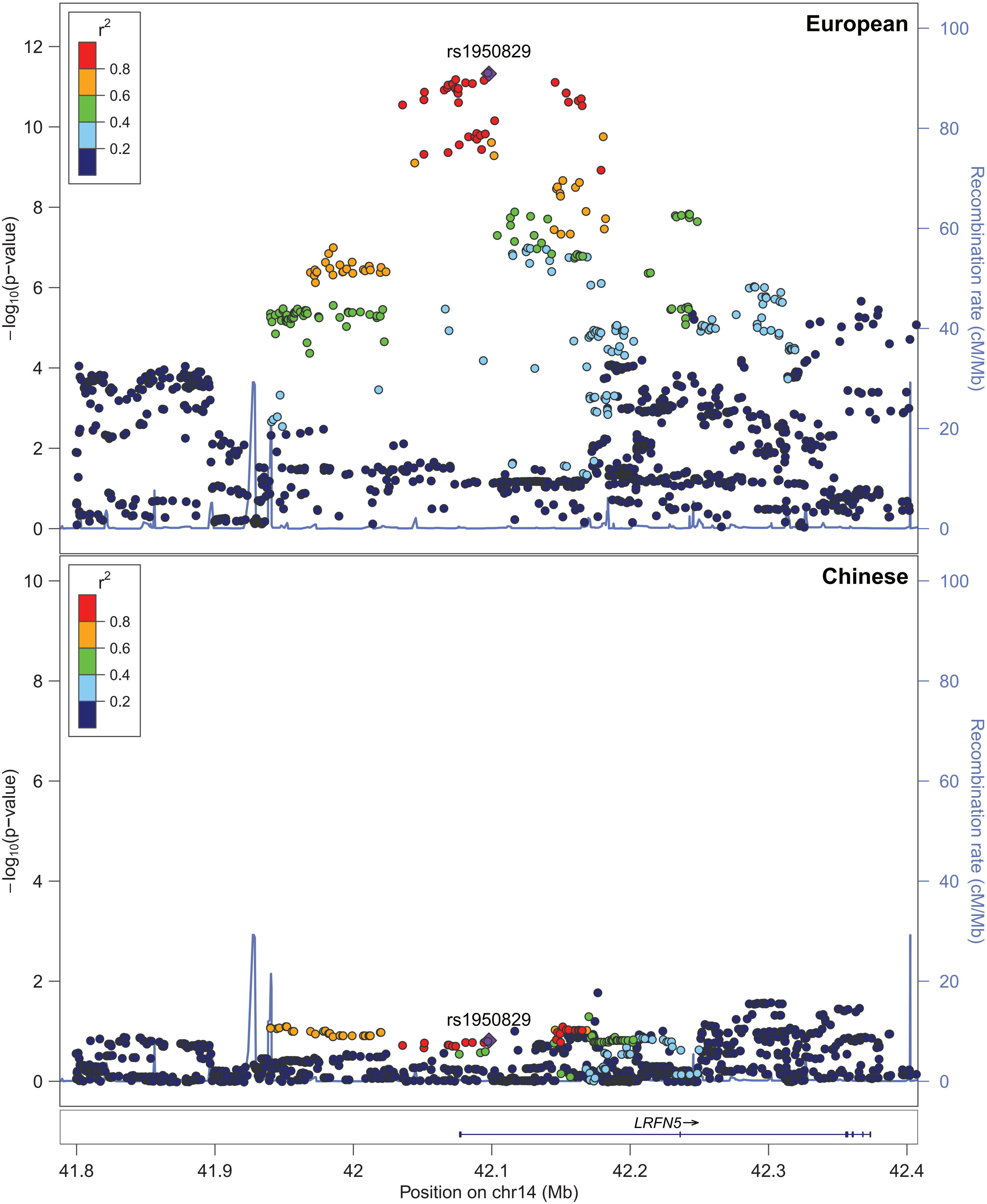

Figure S2. Expression of *DCC* in human tissues according to data from GTEx RNA-seq analysis.

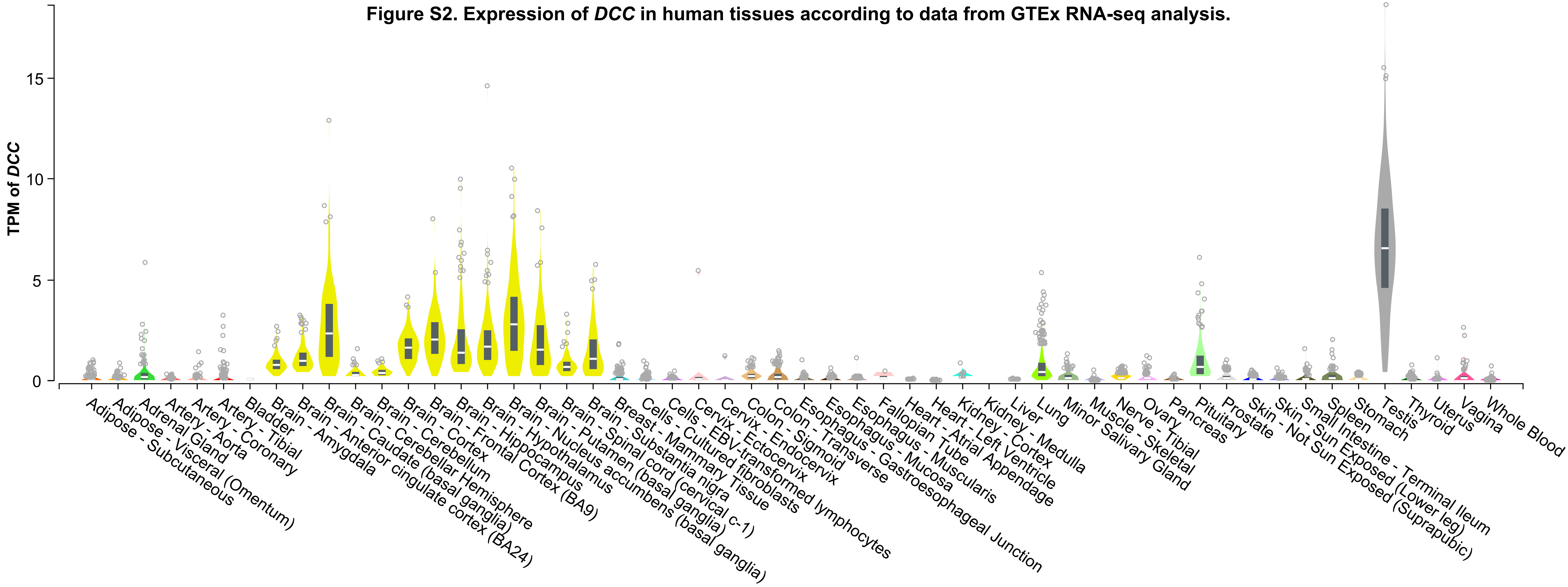

Figure S3. Temporal expression profile of *DCC* in human postmortem dorsolateral prefrontal cortex (DLPFC). The expression level of DCC in human brain is relatively high at early developmental stage. As development continues, *DCC* expression level is decreased.

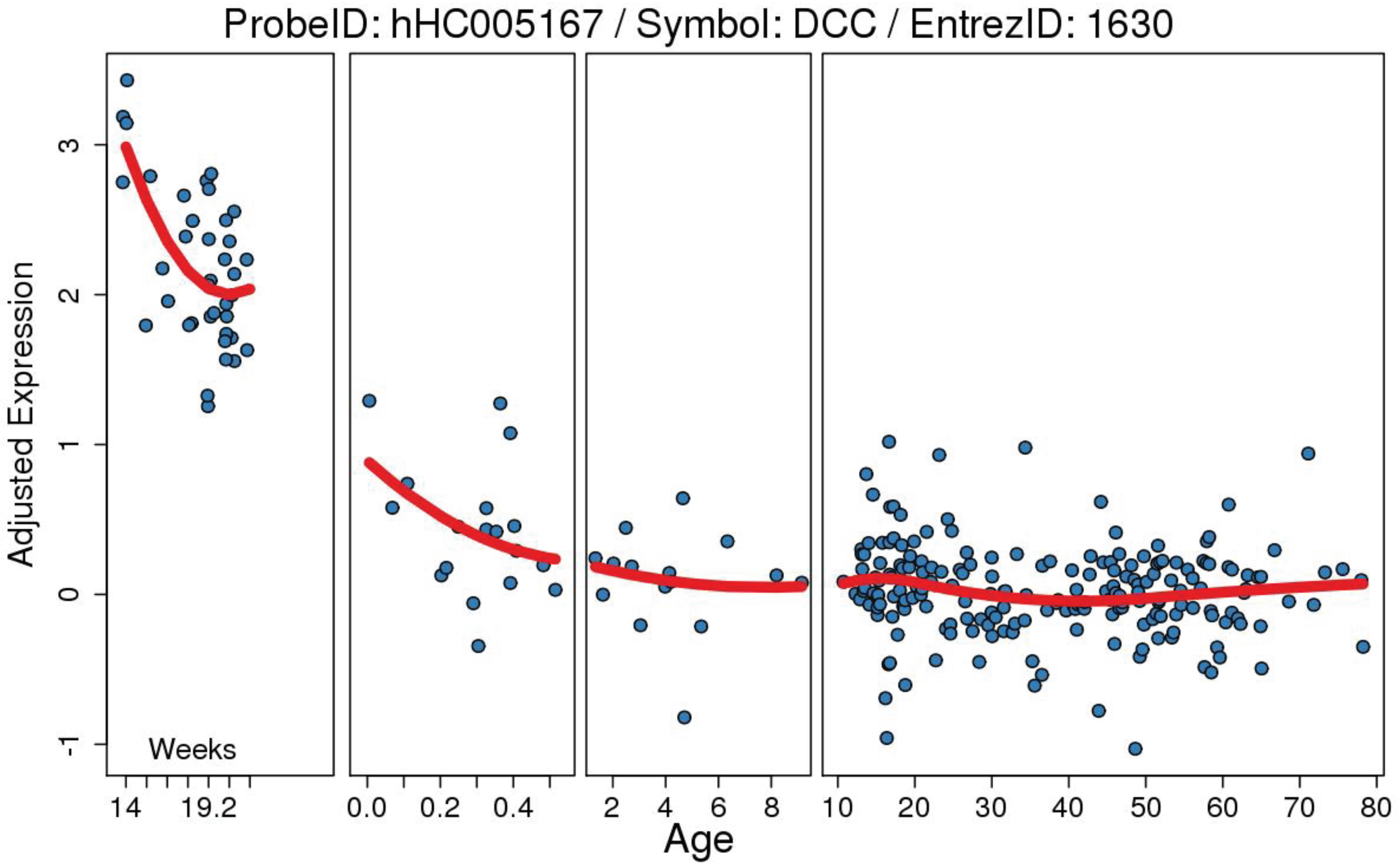

Figure S4. Temporal expression profile of *DCC* in human brain tissues from BrainSpan dataset. Abbreviation: AMY, amygdaloid complex; CB, cerebellum; CBC, cerebellar cortex; DFC, dorsolateral prefrontal cortex; HIP, hippocampus; MFC, anterior (rostral) cingulate (medial prefrontal) cortex; OFC, orbital frontal cortex; STR, striatum; VFC, ventrolateral prefrontal cortex.

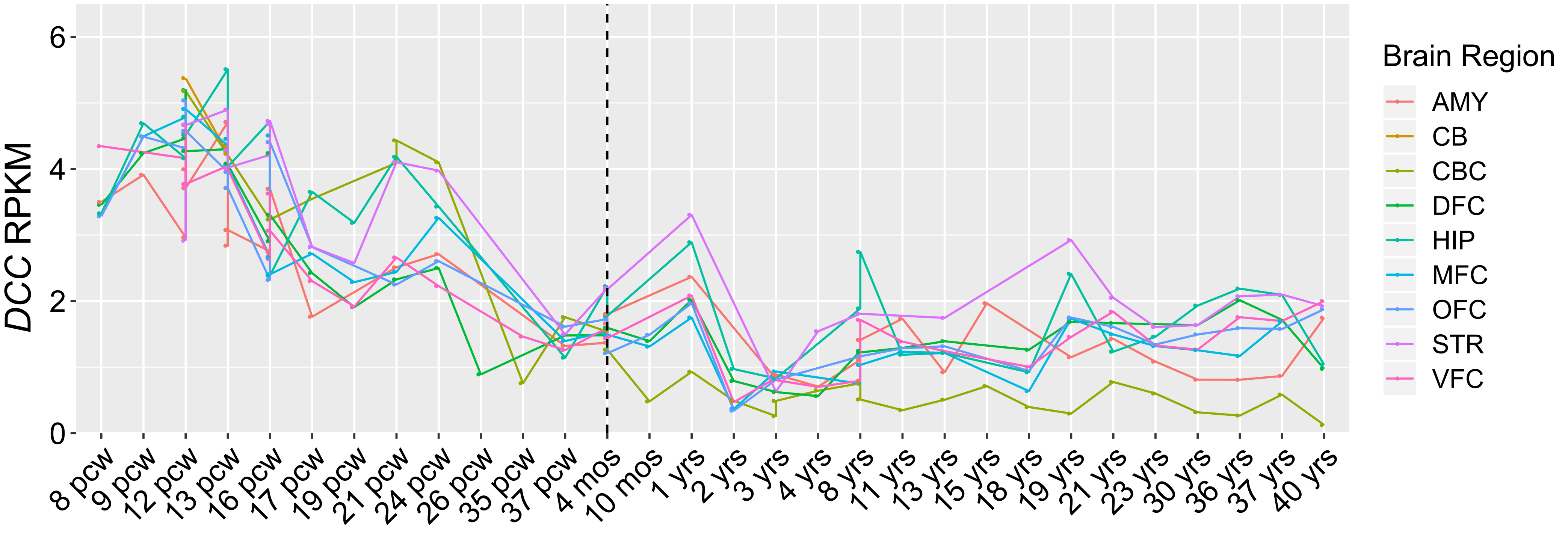

Figure S5. Association of rs7227069 and rs1367635 with 3,999 UK Biobank brain imaging phenotypes and other traits in the dataset (http://big.stats.ox.ac.uk/).

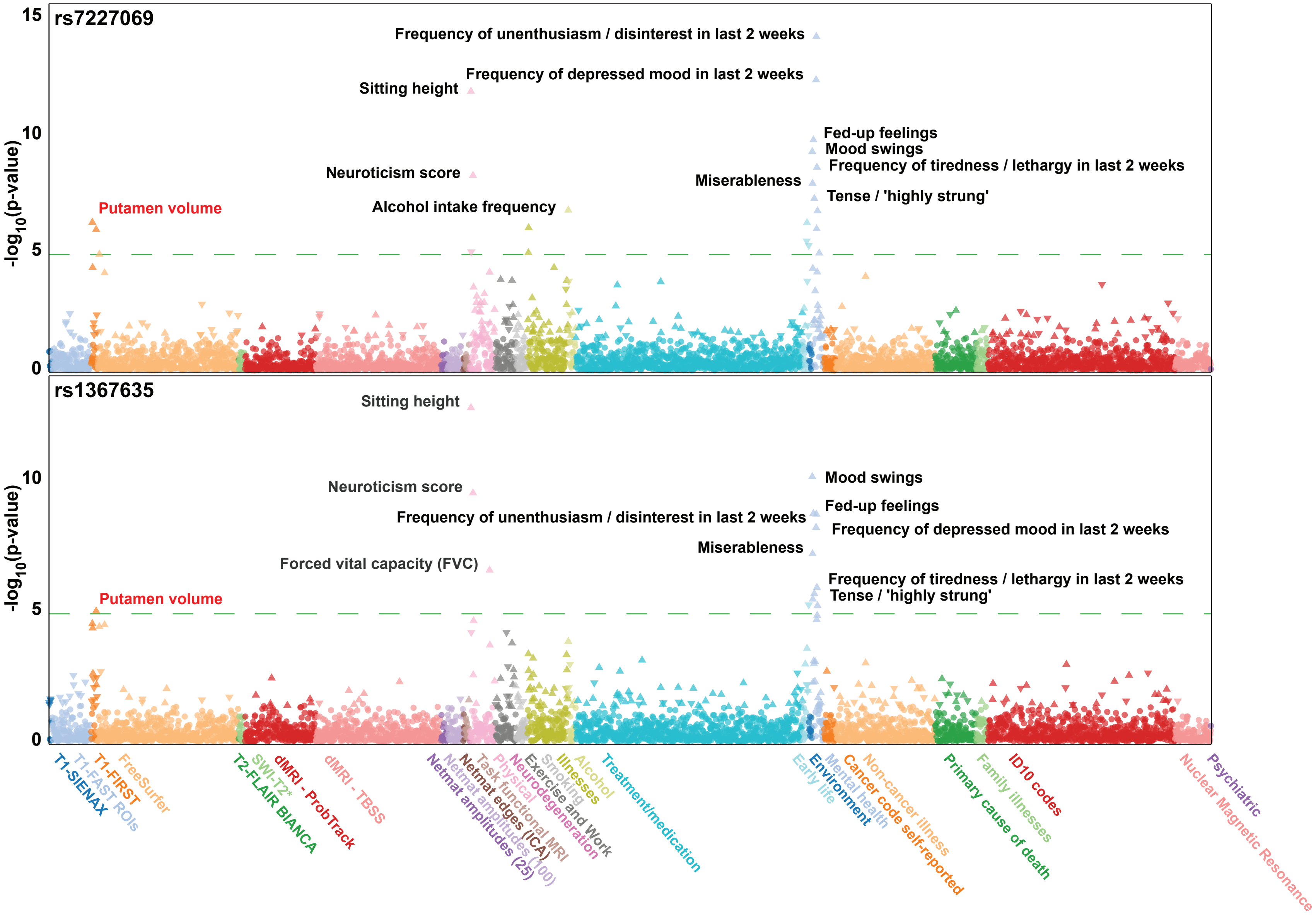

Table S1. iRIGS analysis of the 102 depression risk variants.									
**genename**	**geneID**	**gene_CHR**	**gene_bp**	**gene_biotype**	**snp**	**snp_CHR**	**snp_bp**	**snp_p**	**post_prob**
LPHN2	ENSG00000117114	1	82114976	protein_coding	rs113188507	1	80809636	1.87E-08	1
LSAMP	ENSG00000185565	3	116622368	protein_coding	rs6783233	3	117509984	2.90E-08	1
EYS	ENSG00000188107	6	65423497	protein_coding	rs9363467	6	66565703	6.44E-11	1
PDE10A	ENSG00000112541	6	165908182	protein_coding	rs2029865	6	165121844	1.20E-08	1
PTPRD	ENSG00000153707	9	9463484	protein_coding	rs1982277	9	11513019	1.45E-11	1
PCDH9	ENSG00000184226	13	67340717	protein_coding	rs9592461	13	66941792	9.10E-10	1
LRFN5	ENSG00000165379	14	42225262	protein_coding	rs61990288	14	42074726	1.68E-13	1
RBFOX1	ENSG00000078328	16	6916217	protein_coding	rs7198928	16	7666402	4.45E-11	0.9983
NEGR1	ENSG00000172260	1	72308521	protein_coding	rs10890020	1	73668836	4.03E-15	0.9739
ELAVL2	ENSG00000107105	9	23758218	protein_coding	rs3793577	9	23737627	8.41E-11	0.9704
SPRY2	ENSG00000136158	13	80912598	protein_coding	rs9545360	13	80826373	5.02E-09	0.9303
NR4A2	ENSG00000153234	2	157189902	protein_coding	rs1226412	2	157111313	3.46E-09	0.9233
PDE4B	ENSG00000184588	1	66549228	protein_coding	rs10789214	1	67146817	4.44E-08	0.9199
MEF2C	ENSG00000081189	5	88106948	protein_coding	rs3099439	5	87545318	5.05E-15	0.8990
GPM6A	ENSG00000150625	4	176738950	protein_coding	rs7659414	4	177350956	1.20E-08	0.8275
DCC	ENSG00000187323	18	50464713	protein_coding	rs7227069	18	50731802	1.50E-11	0.8275
HIVEP2	ENSG00000010818	6	143169471	protein_coding	rs2876520	6	142996618	2.29E-10	0.7944
MEIS2	ENSG00000134138	15	37287456	protein_coding	rs8037355	15	37643831	3.94E-11	0.7666
FHIT	ENSG00000189283	3	60486084	protein_coding	rs141954845	3	61192911	8.15E-10	0.7509
BCAT1	ENSG00000060982	12	25033344	protein_coding	rs78337797	12	23987925	3.37E-08	0.7474
PAX6	ENSG00000007372	11	31822924	protein_coding	rs1448938	11	30892824	1.30E-09	0.7317
CELF4	ENSG00000101489	18	34984505	protein_coding	rs12967855	18	35138245	1.18E-12	0.7314
CREB1	ENSG00000118260	2	208431308	protein_coding	rs62188629	2	208044470	7.13E-10	0.7252
TCF4	ENSG00000196628	18	53110790	protein_coding	rs62091461	18	52488672	1.95E-09	0.7143
VRK2	ENSG00000028116	2	58260920	protein_coding	rs1568452	2	58012833	8.12E-12	0.7096
ZNF536	ENSG00000198597	19	30956146	protein_coding	rs33431	19	30939989	4.81E-08	0.7056
MKL2	ENSG00000186260	16	14262904	protein_coding	rs56887639	16	13755530	1.51E-12	0.6934
ASCC3	ENSG00000112249	6	101142659	protein_coding	rs7758630	6	101387304	5.56E-10	0.6880
KIRREL3	ENSG00000149571	11	126583304	protein_coding	rs57344483	11	127022560	1.82E-08	0.6760
CACNA2D1	ENSG00000153956	7	81824437	protein_coding	rs16887442	7	82936909	8.62E-09	0.6620
BHLHE22	ENSG00000180828	8	65494497	protein_coding	rs7837935	8	65562019	3.34E-09	0.6601

WNT2	ENSG00000105989	7	116940014	protein_coding	rs7807677	7	117502574	1.82E-11	0.6566
PTPRC	ENSG00000081237	1	198667173	protein_coding	rs17641524	1	197704717	1.52E-13	0.6516
LARP4B	ENSG00000107929	10	916524	protein_coding	rs997934	10	1795194	4.81E-08	0.6446
TMTC2	ENSG00000179104	12	83304654	protein_coding	rs56314503	12	84465022	2.95E-10	0.6300
AGBL4	ENSG00000186094	1	49744056	protein_coding	rs11579246	1	50559162	5.71E-10	0.5732
BNC2	ENSG00000173068	9	16643622	protein_coding	rs263645	9	17016503	3.70E-10	0.5513
PAX5	ENSG00000196092	9	36933874	protein_coding	rs7030813	9	36999369	3.07E-12	0.5401
DAAM1	ENSG00000100592	14	59746743	protein_coding	rs1956373	14	60141822	2.06E-08	0.5186
NR2F1	ENSG00000175745	5	92924682	protein_coding	rs10061069	5	93071630	8.15E-11	0.5087
LIN28B	ENSG00000187772	6	105468065	protein_coding	rs1933802	6	105365891	2.57E-10	0.5017
HMGB1	ENSG00000189403	13	31112309	protein_coding	rs1409379	13	31907741	1.67E-09	0.4978
PNPLA8	ENSG00000135241	7	108160488	protein_coding	rs58104186	7	109099919	1.82E-11	0.4959
NCOA2	ENSG00000140396	8	71169018	protein_coding	rs67436663	8	71347626	9.37E-10	0.4948
CRYM	ENSG00000103316	16	21282299	protein_coding	rs12923444	16	21639710	1.30E-09	0.4933
KIF2A	ENSG00000068796	5	61717532	protein_coding	rs60157091	5	61509655	1.42E-08	0.4814
GLUL	ENSG00000135821	1	182356090	protein_coding	rs169235	1	181740924	2.98E-08	0.4808
FOS	ENSG00000170345	14	75747205	protein_coding	rs1045430	14	75130235	7.31E-13	0.4616
NFATC1	ENSG00000131196	18	77222548	protein_coding	rs7241572	18	77580712	2.70E-10	0.4599
NCAM1	ENSG00000149294	11	112990577	protein_coding	rs61902811	11	113370758	1.40E-12	0.4594
MAD1L1	ENSG00000002822	7	2064130	protein_coding	rs3823624	7	2110346	1.99E-09	0.4582
UCHL1	ENSG00000154277	4	41264451	protein_coding	rs34937911	4	42110353	4.13E-08	0.4417
SHANK2	ENSG00000162105	11	70638792	protein_coding	rs7117514	11	70544937	7.29E-09	0.4390
SASH1	ENSG00000111961	6	148733313	protein_coding	rs725616	6	147950422	1.87E-08	0.4370
CNTLN	ENSG00000044459	9	17319450	protein_coding	rs263645	9	17016503	3.70E-10	0.4278
ADGB	ENSG00000118492	6	147028349	protein_coding	rs725616	6	147950422	1.87E-08	0.4219
SEMA6D	ENSG00000137872	15	47771359	protein_coding	rs34488670	15	47684936	6.03E-09	0.4164
SMARCA2	ENSG00000080503	9	2104483	protein_coding	rs1354115	9	2983774	7.08E-09	0.4024
RFWD2	ENSG00000143207	1	176043938	protein_coding	rs10913112	1	175913828	3.40E-13	0.3937
LHX2	ENSG00000106689	9	126779764	protein_coding	rs2670139	9	126634255	1.21E-10	0.3893
ZBTB16	ENSG00000109906	11	114025856	protein_coding	rs61902811	11	113370758	1.40E-12	0.3856
LIMD1	ENSG00000144791	3	45662358	protein_coding	rs4346585	3	44736493	7.13E-10	0.3815
GPR183	ENSG00000169508	13	99953221	protein_coding	rs4772087	13	99115041	3.91E-10	0.3746
TMEM106B	ENSG00000106460	7	12263876	protein_coding	rs2043539	7	12253880	9.89E-15	0.3491
NRD1	ENSG00000078618	1	52299670	protein_coding	rs1890946	1	52342427	2.68E-11	0.3473

CTNND1	ENSG00000198561	11	57553866	protein_coding	rs2509805	11	57650796	9.17E-09	0.3467
RERE	ENSG00000142599	1	8645079	protein_coding	rs301799	1	8489302	1.36E-12	0.3440
HACE1	ENSG00000085382	6	105241881	protein_coding	rs1933802	6	105365891	2.57E-10	0.3432
MAFB	ENSG00000204103	20	39316184	protein_coding	rs143186028	20	39997404	2.29E-09	0.3323
NEK5	ENSG00000197168	13	52657153	protein_coding	rs1343605	13	53647048	6.23E-18	0.3314
TNR	ENSG00000116147	1	175502420	protein_coding	rs10913112	1	175913828	3.40E-13	0.3274
CYP7B1	ENSG00000172817	8	65605819	protein_coding	rs7837935	8	65562019	3.34E-09	0.3242
DNAJB9	ENSG00000128590	7	108212653	protein_coding	rs58104186	7	109099919	1.82E-11	0.3195
TLR4	ENSG00000136869	9	120472879	protein_coding	rs10817969	9	119731045	3.11E-11	0.3188
VLDLR	ENSG00000147852	9	2638157	protein_coding	rs1354115	9	2983774	7.08E-09	0.3000
ASTN2	ENSG00000148219	9	119682426	protein_coding	rs10817969	9	119731045	3.11E-11	0.2997
CD40	ENSG00000101017	20	44752706	protein_coding	rs12624433	20	44680853	7.44E-09	0.2997
BRINP2	ENSG00000198797	1	177195810	protein_coding	rs72710803	1	177428018	5.29E-11	0.2979
SCIN	ENSG00000006747	7	12651715	protein_coding	rs2043539	7	12253880	9.89E-15	0.2979
GSTO1	ENSG00000148834	10	106011165	protein_coding	rs1021363	10	106610839	4.41E-16	0.2927
FANCL	ENSG00000115392	2	58427442	protein_coding	rs1568452	2	58012833	8.12E-12	0.2904
SYNE2	ENSG00000054654	14	64506424	protein_coding	rs1152578	14	64697037	6.36E-10	0.2872
SLC6A15	ENSG00000072041	12	85280443	protein_coding	rs56314503	12	84465022	2.95E-10	0.2864
PRKCD	ENSG00000163932	3	53208379	protein_coding	rs7624336	3	53244151	3.96E-08	0.2840
SLK	ENSG00000065613	10	105757975	protein_coding	rs1021363	10	106610839	4.41E-16	0.2840
POU5F2	ENSG00000248483	5	93076776	pseudogene	rs10061069	5	93071630	8.15E-11	0.2822
OLFM4	ENSG00000102837	13	53614513	protein_coding	rs1343605	13	53647048	6.23E-18	0.2766
TYR	ENSG00000077498	11	88969764	protein_coding	rs7932640	11	88744425	1.62E-15	0.2719
HTT	ENSG00000197386	4	3161042	protein_coding	rs7685686	4	3207142	2.57E-08	0.2683
GRM5	ENSG00000168959	11	88518428	protein_coding	rs7932640	11	88744425	1.62E-15	0.2578
IPO11	ENSG00000086200	5	61812104	protein_coding	rs60157091	5	61509655	1.42E-08	0.2561
SOX5	ENSG00000134532	12	23893203	protein_coding	rs78337797	12	23987925	3.37E-08	0.2526
SLC24A5	ENSG00000188467	15	48424019	protein_coding	rs34488670	15	47684936	6.03E-09	0.2526
SIM1	ENSG00000112246	6	100872848	protein_coding	rs7758630	6	101387304	5.56E-10	0.2511
PTPRG	ENSG00000144724	3	61915265	protein_coding	rs141954845	3	61192911	8.15E-10	0.2491
STK24	ENSG00000102572	13	99166324	protein_coding	rs4772087	13	99115041	3.91E-10	0.2456
ZBTB1	ENSG00000126804	14	64985419	protein_coding	rs1152578	14	64697037	6.36E-10	0.2424
ZHX3	ENSG00000174306	20	39876700	protein_coding	rs143186028	20	39997404	2.29E-09	0.2422
LFNG	ENSG00000106003	7	2560487	protein_coding	rs3823624	7	2110346	1.99E-09	0.2396

PAPPA	ENSG00000182752	9	119040342	protein_coding	rs10817969	9	119731045	3.11E-11	0.2387
NOX4	ENSG00000086991	11	89190151	protein_coding	rs7932640	11	88744425	1.62E-15	0.2368
SHOX2	ENSG00000168779	3	157819017	protein_coding	rs1095626	3	157977962	7.13E-14	0.2334
RSRC1	ENSG00000174891	3	158043581	protein_coding	rs1095626	3	157977962	7.13E-14	0.2333
FGF2	ENSG00000138685	4	123783627	protein_coding	rs45510091	4	123186393	1.83E-08	0.2317
FHOD3	ENSG00000134775	18	34118860	protein_coding	rs12967855	18	35138245	1.18E-12	0.2303
SPRED1	ENSG00000166068	15	38596988	protein_coding	rs8037355	15	37643831	3.94E-11	0.2300
EP300	ENSG00000100393	22	41531935	protein_coding	rs5995992	22	41487218	1.30E-11	0.2300
RELA	ENSG00000173039	11	65425816	protein_coding	rs58621819	11	65314830	1.57E-08	0.2241
USPL1	ENSG00000132952	13	31212758	protein_coding	rs1409379	13	31907741	1.67E-09	0.2195
KIAA1109	ENSG00000138688	4	123178700	protein_coding	rs45510091	4	123186393	1.83E-08	0.2178
CHMP3	ENSG00000115561	2	86839399	protein_coding	rs7585722	2	86819128	2.68E-08	0.2160
KDM3A	ENSG00000115548	2	86693804	protein_coding	rs7585722	2	86819128	2.68E-08	0.2158
MLF1	ENSG00000178053	3	158306996	protein_coding	rs1095626	3	157977962	7.13E-14	0.2145
IL10RA	ENSG00000110324	11	117864629	protein_coding	rs2187490	11	118713180	3.82E-08	0.2101
USP4	ENSG00000114316	3	49346704	protein_coding	rs13084037	3	49214066	7.08E-09	0.2087
ATP2A2	ENSG00000174437	12	110753729	protein_coding	rs10774600	12	110741356	3.39E-08	0.2004
ZBTB25	ENSG00000089775	14	64943877	protein_coding	rs1152578	14	64697037	6.36E-10	0.2003
DOCK9	ENSG00000088387	13	99592310	protein_coding	rs4772087	13	99115041	3.91E-10	0.1951
KDM2B	ENSG00000089094	12	121942911	protein_coding	rs3213572	12	121205078	7.61E-10	0.1943
PPM1A	ENSG00000100614	14	60739137	protein_coding	rs1956373	14	60141822	2.06E-08	0.1905
TNRC6B	ENSG00000100354	22	40586316	protein_coding	rs5995992	22	41487218	1.30E-11	0.1882
TRPC3	ENSG00000138741	4	122836545	protein_coding	rs45510091	4	123186393	1.83E-08	0.1874
RPUSD4	ENSG00000165526	11	126076790	protein_coding	rs57344483	11	127022560	1.82E-08	0.1847
ADD1	ENSG00000087274	4	2888693	protein_coding	rs7685686	4	3207142	2.57E-08	0.1829
GTPBP4	ENSG00000107937	10	1050107	protein_coding	rs997934	10	1795194	4.81E-08	0.1812
SULF1	ENSG00000137573	8	70476004	protein_coding	rs67436663	8	71347626	9.37E-10	0.1794
RCOR1	ENSG00000089902	14	103127955	protein_coding	rs10149470	14	104017953	3.72E-14	0.1794
ANO1	ENSG00000131620	11	69980021	protein_coding	rs7117514	11	70544937	7.29E-09	0.1781
SEMA3E	ENSG00000170381	7	83135850	protein_coding	rs2247523	7	82454404	4.38E-09	0.1777
THAP5	ENSG00000177683	7	108202590	protein_coding	rs58104186	7	109099919	1.82E-11	0.1777
ZCCHC7	ENSG00000147905	9	37239341	protein_coding	rs7030813	9	36999369	3.07E-12	0.1755
APBB2	ENSG00000163697	4	41015387	protein_coding	rs34937911	4	42110353	4.13E-08	0.1725
CTTN	ENSG00000085733	11	70263600	protein_coding	rs7117514	11	70544937	7.29E-09	0.1725

ARPC3	ENSG00000111229	12	110880428	protein_coding	rs10774600	12	110741356	3.39E-08	0.1725
KIAA0020	ENSG00000080608	9	2782355	protein_coding	rs1354115	9	2983774	7.08E-09	0.1721
ADARB2	ENSG00000185736	10	1503871	protein_coding	rs997934	10	1795194	4.81E-08	0.1673
CAMTA1	ENSG00000171735	1	7336650	protein_coding	rs301799	1	8489302	1.36E-12	0.1672
RNASEL	ENSG00000135828	1	182550580	protein_coding	rs169235	1	181740924	2.98E-08	0.1655
PLCG1	ENSG00000124181	20	39784980	protein_coding	rs143186028	20	39997404	2.29E-09	0.1638
PHOX2B	ENSG00000109132	4	41748543	protein_coding	rs34937911	4	42110353	4.13E-08	0.1620
CTDP1	ENSG00000060069	18	77477155	protein_coding	rs7241572	18	77580712	2.70E-10	0.1620
MTF1	ENSG00000188786	1	38302571	protein_coding	rs1466887	1	37709328	4.12E-08	0.1608
THSD7A	ENSG00000005108	7	11640944	protein_coding	rs2043539	7	12253880	9.89E-15	0.1596
ARL4A	ENSG00000122644	7	12728520	protein_coding	rs2043539	7	12253880	9.89E-15	0.1533
P2RX4	ENSG00000135124	12	121659784	protein_coding	rs3213572	12	121205078	7.61E-10	0.1481
REEP1	ENSG00000068615	2	86503161	protein_coding	rs7585722	2	86819128	2.68E-08	0.1463
RGS12	ENSG00000159788	4	3368197	protein_coding	rs7685686	4	3207142	2.57E-08	0.1463
PBRM1	ENSG00000163939	3	52649650	protein_coding	rs7624336	3	53244151	3.96E-08	0.1461
TRIM32	ENSG00000119401	9	119456580	protein_coding	rs10817969	9	119731045	3.11E-11	0.1429
FTH1	ENSG00000167996	11	61731161	protein_coding	rs198457	11	61471678	2.99E-10	0.1429
STXBP5	ENSG00000164506	6	147617107	protein_coding	rs725616	6	147950422	1.87E-08	0.1411
CD8A	ENSG00000153563	2	87023624	protein_coding	rs7585722	2	86819128	2.68E-08	0.1394
NEK6	ENSG00000119408	9	127067735	protein_coding	rs2670139	9	126634255	1.21E-10	0.1394
RCN1	ENSG00000049449	11	31980620	protein_coding	rs1448938	11	30892824	1.30E-09	0.1394
PCDH8	ENSG00000136099	13	53420442	protein_coding	rs1343605	13	53647048	6.23E-18	0.1376
DCP1A	ENSG00000162290	3	53349542	processed_transcript	rs7624336	3	53244151	3.96E-08	0.1359
DIMT1	ENSG00000086189	5	61691423	protein_coding	rs60157091	5	61509655	1.42E-08	0.1353
ZNF335	ENSG00000198026	20	44589062	protein_coding	rs12624433	20	44680853	7.44E-09	0.1344
ASTN1	ENSG00000152092	1	176980273	protein_coding	rs10913112	1	175913828	3.40E-13	0.1341
SLC12A1	ENSG00000074803	15	48540068	protein_coding	rs34488670	15	47684936	6.03E-09	0.1324
XPR1	ENSG00000143324	1	180730263	protein_coding	rs169235	1	181740924	2.98E-08	0.1307
RNF10	ENSG00000022840	12	120993340	protein_coding	rs3213572	12	121205078	7.61E-10	0.1307
BATF	ENSG00000156127	14	76001063	protein_coding	rs1045430	14	75130235	7.31E-13	0.1289
STK40	ENSG00000196182	1	36828361	protein_coding	rs1466887	1	37709328	4.12E-08	0.1284
LIMCH1	ENSG00000064042	4	41531842	protein_coding	rs34937911	4	42110353	4.13E-08	0.1262
PHACTR2	ENSG00000112419	6	144005152	protein_coding	rs2876520	6	142996618	2.29E-10	0.1254
TRAM1	ENSG00000067167	8	71503149	protein_coding	rs67436663	8	71347626	9.37E-10	0.1220

RTN1	ENSG00000139970	14	60200189	protein_coding	rs1956373	14	60141822	2.06E-08	0.1220
TXNL4A	ENSG00000141759	18	77740700	protein_coding	rs7241572	18	77580712	2.70E-10	0.1202
THRAP3	ENSG00000054118	1	36730487	protein_coding	rs1466887	1	37709328	4.12E-08	0.1185
DDX6	ENSG00000110367	11	118640946	protein_coding	rs2187490	11	118713180	3.82E-08	0.1185
MYEF2	ENSG00000104177	15	48451169	protein_coding	rs34488670	15	47684936	6.03E-09	0.1185
ST13	ENSG00000100380	22	41236782	protein_coding	rs5995992	22	41487218	1.30E-11	0.1185
KLC1	ENSG00000126214	14	104098060	protein_coding	rs10149470	14	104017953	3.72E-14	0.1167
SLCO5A1	ENSG00000137571	8	70665933	protein_coding	rs67436663	8	71347626	9.37E-10	0.1162
FAF1	ENSG00000185104	1	51165542	protein_coding	rs1890946	1	52342427	2.68E-11	0.1162
POLN	ENSG00000130997	4	2158746	protein_coding	rs7685686	4	3207142	2.57E-08	0.1150
MCTP1	ENSG00000175471	5	94329862	protein_coding	rs10061069	5	93071630	8.15E-11	0.1150
DENND1A	ENSG00000119522	9	126417182	protein_coding	rs2670139	9	126634255	1.21E-10	0.1150
RAB3B	ENSG00000169213	1	52415032	protein_coding	rs1890946	1	52342427	2.68E-11	0.1132
ATP6V1G3	ENSG00000151418	1	198501213	protein_coding	rs17641524	1	197704717	1.52E-13	0.1132
ESR2	ENSG00000140009	14	64677890	protein_coding	rs1152578	14	64697037	6.36E-10	0.1132
EIF5	ENSG00000100664	14	103805621	protein_coding	rs10149470	14	104017953	3.72E-14	0.1132
PARK7	ENSG00000116288	1	8029958	protein_coding	rs301799	1	8489302	1.36E-12	0.1124
EPS15	ENSG00000085832	1	51902467	protein_coding	rs1890946	1	52342427	2.68E-11	0.1115
CFTR	ENSG00000001626	7	117207278	protein_coding	rs7807677	7	117502574	1.82E-11	0.1115
ERRFI1	ENSG00000116285	1	8075416	protein_coding	rs301799	1	8489302	1.36E-12	0.1098
ZCCHC11	ENSG00000134744	1	52946556	protein_coding	rs1890946	1	52342427	2.68E-11	0.1098
MAP3K11	ENSG00000173327	11	65374039	protein_coding	rs58621819	11	65314830	1.57E-08	0.1098
ANAPC7	ENSG00000196510	12	110826120	protein_coding	rs10774600	12	110741356	3.39E-08	0.1097
CLSPN	ENSG00000092853	1	36210693	protein_coding	rs1002656	1	37192741	3.74E-12	0.1080
SH3PXD2A	ENSG00000107957	10	105481793	protein_coding	rs1021363	10	106610839	4.41E-16	0.1080
HSPH1	ENSG00000120694	13	31723643	protein_coding	rs1409379	13	31907741	1.67E-09	0.1067
LHX9	ENSG00000143355	1	197893113	protein_coding	rs17641524	1	197704717	1.52E-13	0.1045
PCLO	ENSG00000186472	7	82587783	protein_coding	rs2247523	7	82454404	4.38E-09	0.1045
METTL9	ENSG00000197006	16	21638666	protein_coding	rs12923444	16	21639710	1.30E-09	0.1045
FOLH1B	ENSG00000134612	11	89401784	pseudogene	rs7932640	11	88744425	1.62E-15	0.1028
ERCC4	ENSG00000175595	16	14030108	protein_coding	rs56887639	16	13755530	1.51E-12	0.1028
PHF12	ENSG00000109118	17	27255528	protein_coding	rs75581564	17	27363750	3.17E-08	0.1028
AHNAK	ENSG00000124942	11	62262367	protein_coding	rs198457	11	61471678	2.99E-10	0.1010
KMT2A	ENSG00000118058	11	118352372	protein_coding	rs2187490	11	118713180	3.82E-08	0.1000

RAB27B	ENSG00000041353	18	52529227	protein_coding	rs62091461	18	52488672	1.95E-09	0.0981
FZD5	ENSG00000163251	2	208630798	protein_coding	rs62188629	2	208044470	7.13E-10	0.0976
TAOK1	ENSG00000160551	17	27794722	protein_coding	rs75581564	17	27363750	3.17E-08	0.0961
BAG5	ENSG00000166170	14	104026024	protein_coding	rs10149470	14	104017953	3.72E-14	0.0958
SPCS3	ENSG00000129128	4	177247255	protein_coding	rs7659414	4	177350956	1.20E-08	0.0941
CCNH	ENSG00000134480	5	86698073	protein_coding	rs3099439	5	87545318	5.05E-15	0.0941
PREP	ENSG00000085377	6	105788199	protein_coding	rs1933802	6	105365891	2.57E-10	0.0941
PARN	ENSG00000140694	16	14628071	protein_coding	rs56887639	16	13755530	1.51E-12	0.0941
NR6A1	ENSG00000148200	9	127406738	protein_coding	rs2670139	9	126634255	1.21E-10	0.0906
CBL	ENSG00000110395	11	119127201	protein_coding	rs2187490	11	118713180	3.82E-08	0.0906
CCNE1	ENSG00000105173	19	30309010	protein_coding	rs33431	19	30939989	4.81E-08	0.0906
VAMP3	ENSG00000049245	1	7836410	protein_coding	rs301799	1	8489302	1.36E-12	0.0889
ELAVL4	ENSG00000162374	1	50591572	protein_coding	rs11579246	1	50559162	5.71E-10	0.0889
MYO18A	ENSG00000196535	17	27453979	protein_coding	rs75581564	17	27363750	3.17E-08	0.0889
CTTNBP2	ENSG00000077063	7	117432449	protein_coding	rs7807677	7	117502574	1.82E-11	0.0871
KAT5	ENSG00000172977	11	65483271	protein_coding	rs58621819	11	65314830	1.57E-08	0.0871
CAMKK2	ENSG00000110931	12	121705804	protein_coding	rs3213572	12	121205078	7.61E-10	0.0871
RPL23A	ENSG00000198242	17	27048894	protein_coding	rs75581564	17	27363750	3.17E-08	0.0871
ATP9B	ENSG00000166377	18	76983836	protein_coding	rs7241572	18	77580712	2.70E-10	0.0868
RNF4	ENSG00000063978	4	2545497	protein_coding	rs7685686	4	3207142	2.57E-08	0.0854
TSPAN19	ENSG00000231738	12	85419074	protein_coding	rs56314503	12	84465022	2.95E-10	0.0836
PAPPA2	ENSG00000116183	1	176623521	protein_coding	rs10913112	1	175913828	3.40E-13	0.0820
CACNA1E	ENSG00000198216	1	181579728	protein_coding	rs169235	1	181740924	2.98E-08	0.0819
POLR1A	ENSG00000068654	2	86290308	protein_coding	rs7585722	2	86819128	2.68E-08	0.0819
SNX8	ENSG00000106266	7	2342679	protein_coding	rs3823624	7	2110346	1.99E-09	0.0819
GLIS3	ENSG00000107249	9	4086259	protein_coding	rs1354115	9	2983774	7.08E-09	0.0819
MELK	ENSG00000165304	9	36625268	protein_coding	rs7030813	9	36999369	3.07E-12	0.0819
GPR18	ENSG00000125245	13	99910483	protein_coding	rs4772087	13	99115041	3.91E-10	0.0819
PPP1R13B	ENSG00000088808	14	104257008	protein_coding	rs10149470	14	104017953	3.72E-14	0.0819
GFM1	ENSG00000168827	3	158386215	protein_coding	rs1095626	3	157977962	7.13E-14	0.0801
ANKRD32	ENSG00000133302	5	94014596	protein_coding	rs10061069	5	93071630	8.15E-11	0.0801
GPD2	ENSG00000115159	2	157381024	protein_coding	rs1226412	2	157111313	3.46E-09	0.0767
MARK3	ENSG00000075413	14	103910934	protein_coding	rs10149470	14	104017953	3.72E-14	0.0767
RNF103	ENSG00000239305	2	86840752	protein_coding	rs7585722	2	86819128	2.68E-08	0.0752

CSF3R	ENSG00000119535	1	36940261	protein_coding	rs1466887	1	37709328	4.12E-08	0.0749
ZC3H12A	ENSG00000163874	1	37945065	protein_coding	rs1466887	1	37709328	4.12E-08	0.0749
ZNF197	ENSG00000186448	3	44658171	protein_coding	rs4346585	3	44736493	7.13E-10	0.0749
C5orf64	ENSG00000178722	5	60990562	protein_coding	rs60157091	5	61509655	1.42E-08	0.0749
UBE2L6	ENSG00000156587	11	57327443	protein_coding	rs2509805	11	57650796	9.17E-09	0.0749
PSMB7	ENSG00000136930	9	127146734	protein_coding	rs2670139	9	126634255	1.21E-10	0.0741
PER3	ENSG00000049246	1	7874808	protein_coding	rs301799	1	8489302	1.36E-12	0.0732
UTP11L	ENSG00000183520	1	38482713	protein_coding	rs1466887	1	37709328	4.12E-08	0.0732
SLC12A5	ENSG00000124140	20	44669559	protein_coding	rs12624433	20	44680853	7.44E-09	0.0732
SEZ6	ENSG00000063015	17	27307688	protein_coding	rs75581564	17	27363750	3.17E-08	0.0729
RPS25	ENSG00000118181	11	118887911	protein_coding	rs2187490	11	118713180	3.82E-08	0.0714
ALOX5AP	ENSG00000132965	13	31324100	protein_coding	rs1409379	13	31907741	1.67E-09	0.0714
DNAH3	ENSG00000158486	16	21057283	protein_coding	rs12923444	16	21639710	1.30E-09	0.0714
MBD2	ENSG00000134046	18	51714564	protein_coding	rs62091461	18	52488672	1.95E-09	0.0709
NCOA5	ENSG00000124160	20	44704107	protein_coding	rs12624433	20	44680853	7.44E-09	0.0697
CLEC3B	ENSG00000163815	3	45060301	protein_coding	rs4346585	3	44736493	7.13E-10	0.0679
CDC25A	ENSG00000164045	3	48214264	protein_coding	rs13084037	3	49214066	7.08E-09	0.0679
SFR1	ENSG00000156384	10	105883979	protein_coding	rs1021363	10	106610839	4.41E-16	0.0679
CDC42EP2	ENSG00000149798	11	65086094	protein_coding	rs58621819	11	65314830	1.57E-08	0.0679
CABP1	ENSG00000157782	12	121091741	protein_coding	rs3213572	12	121205078	7.61E-10	0.0679
TRAF3	ENSG00000131323	14	103310825	protein_coding	rs10149470	14	104017953	3.72E-14	0.0679
DMRTA2	ENSG00000142700	1	50886197	protein_coding	rs11579246	1	50559162	5.71E-10	0.0662
VEPH1	ENSG00000197415	3	157114469	protein_coding	rs1095626	3	157977962	7.13E-14	0.0662
TRPV4	ENSG00000111199	12	110246051	protein_coding	rs10774600	12	110741356	3.39E-08	0.0662
DYNAP	ENSG00000178690	18	52262557	protein_coding	rs62091461	18	52488672	1.95E-09	0.0662
LPIN3	ENSG00000132793	20	39979391	protein_coding	rs143186028	20	39997404	2.29E-09	0.0662
KLF7	ENSG00000118263	2	207985426	protein_coding	rs62188629	2	208044470	7.13E-10	0.0657
SLC45A1	ENSG00000162426	1	8391056	protein_coding	rs301799	1	8489302	1.36E-12	0.0645
METTL21A	ENSG00000144401	2	208468003	protein_coding	rs62188629	2	208044470	7.13E-10	0.0645
COL17A1	ENSG00000065618	10	105818402	protein_coding	rs1021363	10	106610839	4.41E-16	0.0645
SDHAF2	ENSG00000167985	11	61206257	protein_coding	rs198457	11	61471678	2.99E-10	0.0645
VPS36	ENSG00000136100	13	53005801	protein_coding	rs1343605	13	53647048	6.23E-18	0.0645
PTPRT	ENSG00000196090	20	41260001	protein_coding	rs143186028	20	39997404	2.29E-09	0.0645
IL2	ENSG00000109471	4	123375252	protein_coding	rs45510091	4	123186393	1.83E-08	0.0627

NUDT6	ENSG00000170917	4	123828926	protein_coding	rs45510091	4	123186393	1.83E-08	0.0627
CPPED1	ENSG00000103381	16	12827396	protein_coding	rs56887639	16	13755530	1.51E-12	0.0627
TOP1	ENSG00000198900	20	39705292	protein_coding	rs143186028	20	39997404	2.29E-09	0.0614
FHL3	ENSG00000183386	1	38466860	protein_coding	rs1466887	1	37709328	4.12E-08	0.0610
ZNF445	ENSG00000185219	3	44500212	protein_coding	rs4346585	3	44736493	7.13E-10	0.0610
ZKSCAN7	ENSG00000196345	3	44616175	protein_coding	rs4346585	3	44736493	7.13E-10	0.0610
DAGLA	ENSG00000134780	11	61481189	protein_coding	rs198457	11	61471678	2.99E-10	0.0610
SUGT1	ENSG00000165416	13	53244638	protein_coding	rs1343605	13	53647048	6.23E-18	0.0610
URI1	ENSG00000105176	19	30460581	protein_coding	rs33431	19	30939989	4.81E-08	0.0610
POU3F1	ENSG00000185668	1	38510986	protein_coding	rs1466887	1	37709328	4.12E-08	0.0592
MFSD1	ENSG00000118855	3	158498747	protein_coding	rs1095626	3	157977962	7.13E-14	0.0592
CAPZA2	ENSG00000198898	7	116505219	protein_coding	rs7807677	7	117502574	1.82E-11	0.0592
GSTO2	ENSG00000065621	10	106044123	protein_coding	rs1021363	10	106610839	4.41E-16	0.0592
MYO1H	ENSG00000174527	12	109859926	protein_coding	rs10774600	12	110741356	3.39E-08	0.0592
IGSF6	ENSG00000140749	16	21658295	protein_coding	rs12923444	16	21639710	1.30E-09	0.0592
CD8B	ENSG00000172116	2	87065754	protein_coding	rs7585722	2	86819128	2.68E-08	0.0575
ARIH2	ENSG00000177479	3	48990034	protein_coding	rs13084037	3	49214066	7.08E-09	0.0575
OBFC1	ENSG00000107960	10	105660131	protein_coding	rs1021363	10	106610839	4.41E-16	0.0575
DRD2	ENSG00000149295	11	113313365	protein_coding	rs61902811	11	113370758	1.40E-12	0.0575
MYL2	ENSG00000111245	12	111353582	protein_coding	rs10774600	12	110741356	3.39E-08	0.0575
FLOT2	ENSG00000132589	17	27215532	protein_coding	rs75581564	17	27363750	3.17E-08	0.0575
C19orf12	ENSG00000131943	19	30198423	protein_coding	rs33431	19	30939989	4.81E-08	0.0575
CC2D1B	ENSG00000154222	1	52821630	protein_coding	rs1890946	1	52342427	2.68E-11	0.0557
MR1	ENSG00000153029	1	181017070	protein_coding	rs169235	1	181740924	2.98E-08	0.0557
GRIK2	ENSG00000164418	6	102182311	protein_coding	rs7758630	6	101387304	5.56E-10	0.0557
RBM26	ENSG00000139746	13	79933287	protein_coding	rs9545360	13	80826373	5.02E-09	0.0557
NEK9	ENSG00000119638	14	75571434	protein_coding	rs1045430	14	75130235	7.31E-13	0.0557
MKL1	ENSG00000196588	22	40919495	protein_coding	rs5995992	22	41487218	1.30E-11	0.0557
XRCC6	ENSG00000196419	22	42038583	protein_coding	rs5995992	22	41487218	1.30E-11	0.0557
SFMBT1	ENSG00000163935	3	53009229	protein_coding	rs7624336	3	53244151	3.96E-08	0.0540
ADNP2	ENSG00000101544	18	77886160	protein_coding	rs7241572	18	77580712	2.70E-10	0.0540
TDRD9	ENSG00000156414	14	104456901	protein_coding	rs10149470	14	104017953	3.72E-14	0.0523
CDKN2C	ENSG00000123080	1	51433361	protein_coding	rs1890946	1	52342427	2.68E-11	0.0523
MYRF	ENSG00000124920	11	61538052	protein_coding	rs198457	11	61471678	2.99E-10	0.0523

NEK3	ENSG00000136098	13	52720385	protein_coding	rs1343605	13	53647048	6.23E-18	0.0523
KIF26A	ENSG00000066735	14	104626145	protein_coding	rs10149470	14	104017953	3.72E-14	0.0523
FADD	ENSG00000168040	11	70051382	protein_coding	rs7117514	11	70544937	7.29E-09	0.0505
ZNF660	ENSG00000144792	3	44630450	protein_coding	rs4346585	3	44736493	7.13E-10	0.0488
ZNF35	ENSG00000169981	3	44696251	protein_coding	rs4346585	3	44736493	7.13E-10	0.0488
KIF15	ENSG00000163808	3	44859038	protein_coding	rs4346585	3	44736493	7.13E-10	0.0488
ATP8A1	ENSG00000124406	4	42534756	protein_coding	rs34937911	4	42110353	4.13E-08	0.0488
SERPING1	ENSG00000149131	11	57373593	protein_coding	rs2509805	11	57650796	9.17E-09	0.0488
FAU	ENSG00000149806	11	64889022	protein_coding	rs58621819	11	65314830	1.57E-08	0.0488
SIPA1	ENSG00000213445	11	65411984	protein_coding	rs58621819	11	65314830	1.57E-08	0.0488
PXN	ENSG00000089159	12	120675912	protein_coding	rs3213572	12	121205078	7.61E-10	0.0488
FARP1	ENSG00000152767	13	98948728	protein_coding	rs4772087	13	99115041	3.91E-10	0.0488
POLR3E	ENSG00000058600	16	22327577	protein_coding	rs12923444	16	21639710	1.30E-09	0.0488
RAB34	ENSG00000109113	17	27043373	protein_coding	rs75581564	17	27363750	3.17E-08	0.0488
OR10W1	ENSG00000172772	11	58034998	protein_coding	rs2509805	11	57650796	9.17E-09	0.0470
SIX1	ENSG00000126778	14	61117554	protein_coding	rs1956373	14	60141822	2.06E-08	0.0470
SNX29	ENSG00000048471	16	12369370	protein_coding	rs7200826	16	13066833	3.74E-12	0.0470
NUFIP2	ENSG00000108256	17	27602010	protein_coding	rs75581564	17	27363750	3.17E-08	0.0470
EMILIN3	ENSG00000183798	20	39992036	protein_coding	rs143186028	20	39997404	2.29E-09	0.0470
ELMO2	ENSG00000062598	20	45028196	protein_coding	rs12624433	20	44680853	7.44E-09	0.0470
GRIK3	ENSG00000163873	1	37380429	protein_coding	rs1466887	1	37709328	4.12E-08	0.0453
DAG1	ENSG00000173402	3	49539597	protein_coding	rs13084037	3	49214066	7.08E-09	0.0453
ZFYVE28	ENSG00000159733	4	2345849	protein_coding	rs7685686	4	3207142	2.57E-08	0.0453
CCNA2	ENSG00000145386	4	122741343	protein_coding	rs45510091	4	123186393	1.83E-08	0.0453
SEMA3A	ENSG00000075213	7	83854849	protein_coding	rs16887442	7	82936909	8.62E-09	0.0453
TRIM64B	ENSG00000189253	11	89605817	protein_coding	rs7932640	11	88744425	1.62E-15	0.0453
SRPR	ENSG00000182934	11	126135936	protein_coding	rs57344483	11	127022560	1.82E-08	0.0453
TCTN1	ENSG00000204852	12	111069533	protein_coding	rs10774600	12	110741356	3.39E-08	0.0453
TRAF4	ENSG00000076604	17	27074505	protein_coding	rs75581564	17	27363750	3.17E-08	0.0453
RASAL2	ENSG00000075391	1	178255754	protein_coding	rs72710803	1	177428018	5.29E-11	0.0436
GLYCTK	ENSG00000168237	3	52325188	protein_coding	rs7624336	3	53244151	3.96E-08	0.0436
LXN	ENSG00000079257	3	158377046	protein_coding	rs1095626	3	157977962	7.13E-14	0.0436
RARRES1	ENSG00000118849	3	158432583	protein_coding	rs1095626	3	157977962	7.13E-14	0.0436
DCDC5	ENSG00000170959	11	31000564	protein_coding	rs1448938	11	30892824	1.30E-09	0.0436

CD5	ENSG00000110448	11	60882595	protein_coding	rs198457	11	61471678	2.99E-10	0.0436
PPTC7	ENSG00000196850	12	110995122	protein_coding	rs10774600	12	110741356	3.39E-08	0.0436
HSPA2	ENSG00000126803	14	65006289	protein_coding	rs1152578	14	64697037	6.36E-10	0.0436
XRCC3	ENSG00000126215	14	104172893	protein_coding	rs10149470	14	104017953	3.72E-14	0.0436
DUT	ENSG00000128951	15	48629389	protein_coding	rs34488670	15	47684936	6.03E-09	0.0436
UBE2C	ENSG00000175063	20	44443405	protein_coding	rs12624433	20	44680853	7.44E-09	0.0433
RABGAP1L	ENSG00000152061	1	174546496	protein_coding	rs10913112	1	175913828	3.40E-13	0.0418
RBM5	ENSG00000003756	3	50141397	protein_coding	rs13084037	3	49214066	7.08E-09	0.0418
VEGFC	ENSG00000150630	4	177659285	protein_coding	rs7659414	4	177350956	1.20E-08	0.0418
ZDHHC5	ENSG00000156599	11	57451939	protein_coding	rs2509805	11	57650796	9.17E-09	0.0418
DHCR7	ENSG00000172893	11	71151576	protein_coding	rs7117514	11	70544937	7.29E-09	0.0418
CTSC	ENSG00000109861	11	88048864	protein_coding	rs7932640	11	88744425	1.62E-15	0.0418
ST3GAL4	ENSG00000110080	11	126267887	protein_coding	rs57344483	11	127022560	1.82E-08	0.0418
KCNG2	ENSG00000178342	18	77641742	protein_coding	rs7241572	18	77580712	2.70E-10	0.0418
PQLC1	ENSG00000122490	18	77687042	protein_coding	rs7241572	18	77580712	2.70E-10	0.0418
XPNPEP3	ENSG00000196236	22	41310833	protein_coding	rs5995992	22	41487218	1.30E-11	0.0418
TKT	ENSG00000163931	3	53274395	protein_coding	rs7624336	3	53244151	3.96E-08	0.0403
LSM10	ENSG00000181817	1	36860166	protein_coding	rs1466887	1	37709328	4.12E-08	0.0401
TGM4	ENSG00000163810	3	44936291	protein_coding	rs4346585	3	44736493	7.13E-10	0.0401
ZSWIM6	ENSG00000130449	5	60735048	protein_coding	rs60157091	5	61509655	1.42E-08	0.0401
VWDE	ENSG00000146530	7	12407039	protein_coding	rs2043539	7	12253880	9.89E-15	0.0401
NR5A1	ENSG00000136931	9	127256612	protein_coding	rs2670139	9	126634255	1.21E-10	0.0401
OR9Q1	ENSG00000186509	11	57870220	protein_coding	rs2509805	11	57650796	9.17E-09	0.0401
FADS1	ENSG00000149485	11	61607362	protein_coding	rs198457	11	61471678	2.99E-10	0.0401
CDR2	ENSG00000140743	16	22402871	protein_coding	rs12923444	16	21639710	1.30E-09	0.0401
TIAF1	ENSG00000221995	17	27409537	protein_coding	rs75581564	17	27363750	3.17E-08	0.0401
SSH2	ENSG00000141298	17	28105063	protein_coding	rs75581564	17	27363750	3.17E-08	0.0401
SLC25A17	ENSG00000100372	22	41190518	protein_coding	rs5995992	22	41487218	1.30E-11	0.0401
FGF4	ENSG00000075388	11	69588984	protein_coding	rs7117514	11	70544937	7.29E-09	0.0396
NAA38	ENSG00000183011	17	7774297	protein_coding	rs7807677	7	117502574	1.82E-11	0.0385
DENND1B	ENSG00000213047	1	197609352	protein_coding	rs17641524	1	197704717	1.52E-13	0.0383
COL7A1	ENSG00000114270	3	48617103	protein_coding	rs13084037	3	49214066	7.08E-09	0.0383
EIF3B	ENSG00000106263	7	2407050	protein_coding	rs3823624	7	2110346	1.99E-09	0.0383
RPL35	ENSG00000136942	9	127622209	protein_coding	rs2670139	9	126634255	1.21E-10	0.0383

LPXN	ENSG00000110031	11	58320018	protein_coding	rs2509805	11	57650796	9.17E-09	0.0383
P2RX7	ENSG00000089041	12	121597249	protein_coding	rs3213572	12	121205078	7.61E-10	0.0383
ORAI1	ENSG00000182500	12	122072519	protein_coding	rs3213572	12	121205078	7.61E-10	0.0383
BRCA2	ENSG00000139618	13	32931708	protein_coding	rs1409379	13	31907741	1.67E-09	0.0383
CKB	ENSG00000166165	14	103987722	protein_coding	rs10149470	14	104017953	3.72E-14	0.0383
TPGS2	ENSG00000134779	18	34392596	protein_coding	rs12967855	18	35138245	1.18E-12	0.0383
CTSA	ENSG00000064601	20	44523525	protein_coding	rs12624433	20	44680853	7.44E-09	0.0383
MAP7D1	ENSG00000116871	1	36633815	protein_coding	rs1002656	1	37192741	3.74E-12	0.0366
SF3A3	ENSG00000183431	1	38439620	protein_coding	rs1466887	1	37709328	4.12E-08	0.0366
MIER1	ENSG00000198160	1	67422440	protein_coding	rs10789214	1	67146817	4.44E-08	0.0366
QRICH1	ENSG00000198218	3	49099468	protein_coding	rs13084037	3	49214066	7.08E-09	0.0366
WDR82	ENSG00000164091	3	52305236	protein_coding	rs7624336	3	53244151	3.96E-08	0.0366
GPER1	ENSG00000164850	7	1127647	protein_coding	rs3823624	7	2110346	1.99E-09	0.0366
RFX3	ENSG00000080298	9	3372150	protein_coding	rs1354115	9	2983774	7.08E-09	0.0366
ARL14EP	ENSG00000152219	11	30352186	protein_coding	rs1448938	11	30892824	1.30E-09	0.0366
ASRGL1	ENSG00000162174	11	62132901	protein_coding	rs198457	11	61471678	2.99E-10	0.0366
CD3E	ENSG00000198851	11	118181075	protein_coding	rs2187490	11	118713180	3.82E-08	0.0366
C12orf76	ENSG00000174456	12	110488506	protein_coding	rs10774600	12	110741356	3.39E-08	0.0366
MSI1	ENSG00000135097	12	120793058	protein_coding	rs3213572	12	121205078	7.61E-10	0.0366
JDP2	ENSG00000140044	14	75917616	protein_coding	rs1045430	14	75130235	7.31E-13	0.0366
CTXN2	ENSG00000233932	15	48489844	protein_coding	rs34488670	15	47684936	6.03E-09	0.0366
TSHZ3	ENSG00000121297	19	31803152	protein_coding	rs33431	19	30939989	4.81E-08	0.0366
L3MBTL2	ENSG00000100395	22	41614242	protein_coding	rs5995992	22	41487218	1.30E-11	0.0366
NEK7	ENSG00000151414	1	198208821	protein_coding	rs17641524	1	197704717	1.52E-13	0.0348
TNIP2	ENSG00000168884	4	2750739	protein_coding	rs7685686	4	3207142	2.57E-08	0.0348
SPATA5	ENSG00000145375	4	124040019	protein_coding	rs45510091	4	123186393	1.83E-08	0.0348
ARPC5L	ENSG00000136950	9	127632206	protein_coding	rs2670139	9	126634255	1.21E-10	0.0348
ITPRIP	ENSG00000148841	10	106085028	protein_coding	rs1021363	10	106610839	4.41E-16	0.0348
CPSF7	ENSG00000149532	11	61183812	protein_coding	rs198457	11	61471678	2.99E-10	0.0348
CUX2	ENSG00000111249	12	111630093	protein_coding	rs10774600	12	110741356	3.39E-08	0.0348
JKAMP	ENSG00000050130	14	59961295	protein_coding	rs1956373	14	60141822	2.06E-08	0.0348
BFAR	ENSG00000103429	16	14744882	protein_coding	rs56887639	16	13755530	1.51E-12	0.0348
TMEM159	ENSG00000011638	16	21180817	protein_coding	rs12923444	16	21639710	1.30E-09	0.0348
NEK8	ENSG00000160602	17	27062803	protein_coding	rs75581564	17	27363750	3.17E-08	0.0348

RHOA	ENSG00000067560	3	49423504	protein_coding	rs13084037	3	49214066	7.08E-09	0.0331
EXOSC9	ENSG00000123737	4	122730324	protein_coding	rs45510091	4	123186393	1.83E-08	0.0331
BVES	ENSG00000112276	6	105564873	protein_coding	rs1933802	6	105365891	2.57E-10	0.0331
MAFK	ENSG00000198517	7	1576514	protein_coding	rs3823624	7	2110346	1.99E-09	0.0331
SCYL1	ENSG00000142186	11	65299361	protein_coding	rs58621819	11	65314830	1.57E-08	0.0331
OASL	ENSG00000135114	12	121467583	protein_coding	rs3213572	12	121205078	7.61E-10	0.0331
GPR135	ENSG00000181619	14	59913900	protein_coding	rs1956373	14	60141822	2.06E-08	0.0331
ZP2	ENSG00000103310	16	21217302	protein_coding	rs12923444	16	21639710	1.30E-09	0.0331
MMP9	ENSG00000100985	20	44641373	protein_coding	rs12624433	20	44680853	7.44E-09	0.0331
ORC1	ENSG00000085840	1	52854316	protein_coding	rs1890946	1	52342427	2.68E-11	0.0314
ZNF502	ENSG00000196653	3	44759729	protein_coding	rs4346585	3	44736493	7.13E-10	0.0314
NISCH	ENSG00000010322	3	52508110	protein_coding	rs7624336	3	53244151	3.96E-08	0.0314
STAB1	ENSG00000010327	3	52543932	protein_coding	rs7624336	3	53244151	3.96E-08	0.0314
ANXA5	ENSG00000164111	4	122603689	protein_coding	rs45510091	4	123186393	1.83E-08	0.0314
ASZ1	ENSG00000154438	7	117035726	protein_coding	rs7807677	7	117502574	1.82E-11	0.0314
GNE	ENSG00000159921	9	36245745	protein_coding	rs7030813	9	36999369	3.07E-12	0.0314
SORCS3	ENSG00000156395	10	106712926	protein_coding	rs1021363	10	106610839	4.41E-16	0.0314
CLP1	ENSG00000172409	11	57422902	protein_coding	rs2509805	11	57650796	9.17E-09	0.0314
INCENP	ENSG00000149503	11	61906040	protein_coding	rs198457	11	61471678	2.99E-10	0.0314
PPFIA1	ENSG00000131626	11	70173657	protein_coding	rs7117514	11	70544937	7.29E-09	0.0314
GIT2	ENSG00000139436	12	110400900	protein_coding	rs10774600	12	110741356	3.39E-08	0.0314
HNRNPA1L	ENSG00000139675	13	53204762	protein_coding	rs1343605	13	53647048	6.23E-18	0.0314
ZNF410	ENSG00000119725	14	74376267	protein_coding	rs1045430	14	75130235	7.31E-13	0.0314
TMED10	ENSG00000170348	14	75620753	protein_coding	rs1045430	14	75130235	7.31E-13	0.0314
SLC2A10	ENSG00000197496	20	45351545	protein_coding	rs12624433	20	44680853	7.44E-09	0.0314
RBX1	ENSG00000100387	22	41358332	protein_coding	rs5995992	22	41487218	1.30E-11	0.0314
ADAD1	ENSG00000164113	4	123325539	protein_coding	rs45510091	4	123186393	1.83E-08	0.0304
RGS16	ENSG00000143333	1	182570650	protein_coding	rs169235	1	181740924	2.98E-08	0.0296
QARS	ENSG00000172053	3	49137959	protein_coding	rs13084037	3	49214066	7.08E-09	0.0296
TMEM110	ENSG00000213533	3	52900923	protein_coding	rs7624336	3	53244151	3.96E-08	0.0296
IZUMO3	ENSG00000205442	9	24544451	protein_coding	rs3793577	9	23737627	8.41E-11	0.0296
GRHPR	ENSG00000137106	9	37429825	protein_coding	rs7030813	9	36999369	3.07E-12	0.0296
ZBTB5	ENSG00000168795	9	37451753	protein_coding	rs7030813	9	36999369	3.07E-12	0.0296
CD6	ENSG00000013725	11	60763482	protein_coding	rs198457	11	61471678	2.99E-10	0.0296

DYNLL1	ENSG00000088986	12	120921974	protein_coding	rs3213572	12	121205078	7.61E-10	0.0296
SH3BP2	ENSG00000087266	4	2818787	protein_coding	rs7685686	4	3207142	2.57E-08	0.0281
GPX1	ENSG00000233276	3	49395321	protein_coding	rs13084037	3	49214066	7.08E-09	0.0279
POPDC3	ENSG00000132429	6	105617012	protein_coding	rs1933802	6	105365891	2.57E-10	0.0279
LACTB2	ENSG00000147592	8	71564481	protein_coding	rs67436663	8	71347626	9.37E-10	0.0279
CRB2	ENSG00000148204	9	126130526	protein_coding	rs2670139	9	126634255	1.21E-10	0.0279
CCDC86	ENSG00000110104	11	60614049	protein_coding	rs198457	11	61471678	2.99E-10	0.0279
BEST1	ENSG00000167995	11	61725140	protein_coding	rs198457	11	61471678	2.99E-10	0.0279
VPS11	ENSG00000160695	11	118945545	processed_transcript	rs2187490	11	118713180	3.82E-08	0.0279
ABCD4	ENSG00000119688	14	74760942	protein_coding	rs1045430	14	75130235	7.31E-13	0.0279
TEF	ENSG00000167074	22	41779333	protein_coding	rs5995992	22	41487218	1.30E-11	0.0279
INPP5B	ENSG00000204084	1	38369549	protein_coding	rs1466887	1	37709328	4.12E-08	0.0261
SGIP1	ENSG00000118473	1	67106524	protein_coding	rs10789214	1	67146817	4.44E-08	0.0261
FPGT	ENSG00000254685	1	74681846	protein_coding	rs10890020	1	73668836	4.03E-15	0.0261
ZBTB41	ENSG00000177888	1	197148622	protein_coding	rs17641524	1	197704717	1.52E-13	0.0261
RNF123	ENSG00000164068	3	49742947	protein_coding	rs13084037	3	49214066	7.08E-09	0.0261
RBM6	ENSG00000004534	3	50057459	protein_coding	rs13084037	3	49214066	7.08E-09	0.0261
PTX3	ENSG00000163661	3	157157997	protein_coding	rs1095626	3	157977962	7.13E-14	0.0261
FSHB	ENSG00000131808	11	30254685	protein_coding	rs1448938	11	30892824	1.30E-09	0.0261
RAB38	ENSG00000123892	11	87877533	protein_coding	rs7932640	11	88744425	1.62E-15	0.0261
BCL9L	ENSG00000186174	11	118780450	protein_coding	rs2187490	11	118713180	3.82E-08	0.0261
B3GALTL	ENSG00000187676	13	31840243	protein_coding	rs1409379	13	31907741	1.67E-09	0.0261
DLST	ENSG00000119689	14	75359521	protein_coding	rs1045430	14	75130235	7.31E-13	0.0261
SLC46A1	ENSG00000076351	17	26727444	protein_coding	rs75581564	17	27363750	3.17E-08	0.0261
PLEKHF1	ENSG00000166289	19	30161351	protein_coding	rs33431	19	30939989	4.81E-08	0.0261
SLC43A3	ENSG00000134802	11	57184740	protein_coding	rs2509805	11	57650796	9.17E-09	0.0254
ZNF501	ENSG00000186446	3	44774831	protein_coding	rs4346585	3	44736493	7.13E-10	0.0244
IP6K2	ENSG00000068745	3	48751611	protein_coding	rs13084037	3	49214066	7.08E-09	0.0244
GPR126	ENSG00000112414	6	142695197	protein_coding	rs2876520	6	142996618	2.29E-10	0.0244
PRDM14	ENSG00000147596	8	70973907	protein_coding	rs67436663	8	71347626	9.37E-10	0.0244
FEN1	ENSG00000168496	11	61562412	protein_coding	rs198457	11	61471678	2.99E-10	0.0244
FADS2	ENSG00000134824	11	61597639	protein_coding	rs198457	11	61471678	2.99E-10	0.0244
VPS29	ENSG00000111237	12	110934412	protein_coding	rs10774600	12	110741356	3.39E-08	0.0244
ACADS	ENSG00000122971	12	121170674	protein_coding	rs3213572	12	121205078	7.61E-10	0.0244

SIX6	ENSG00000184302	14	60977618	protein_coding	rs1956373	14	60141822	2.06E-08	0.0244
POLI	ENSG00000101751	18	51808495	protein_coding	rs12966052	18	52751639	1.25E-11	0.0244
EYA1	ENSG00000104313	8	72192067	protein_coding	rs67436663	8	71347626	9.37E-10	0.0231
LARS2	ENSG00000011376	3	45510455	protein_coding	rs4346585	3	44736493	7.13E-10	0.0226
PRKAR2A	ENSG00000114302	3	48833654	protein_coding	rs13084037	3	49214066	7.08E-09	0.0226
TWF2	ENSG00000247596	3	52267901	protein_coding	rs7624336	3	53244151	3.96E-08	0.0226
GNL3	ENSG00000163938	3	52721840	protein_coding	rs7624336	3	53244151	3.96E-08	0.0226
DOK7	ENSG00000175920	4	3484116	protein_coding	rs7685686	4	3207142	2.57E-08	0.0226
INTS1	ENSG00000164880	7	1527701	protein_coding	rs3823624	7	2110346	1.99E-09	0.0226
TNKS1BP1	ENSG00000149115	11	57079769	protein_coding	rs2509805	11	57650796	9.17E-09	0.0226
OR5B21	ENSG00000198283	11	58275113	protein_coding	rs2509805	11	57650796	9.17E-09	0.0226
TMEM109	ENSG00000110108	11	60686130	protein_coding	rs198457	11	61471678	2.99E-10	0.0226
NRXN2	ENSG00000110076	11	64432153	protein_coding	rs58621819	11	65314830	1.57E-08	0.0226
EHD1	ENSG00000110047	11	64637441	protein_coding	rs58621819	11	65314830	1.57E-08	0.0226
NNMT	ENSG00000166741	11	114156258	protein_coding	rs61902811	11	113370758	1.40E-12	0.0226
UPK2	ENSG00000110375	11	118812571	protein_coding	rs2187490	11	118713180	3.82E-08	0.0226
FOXN4	ENSG00000139445	12	109731404	protein_coding	rs10774600	12	110741356	3.39E-08	0.0226
HNF1A	ENSG00000135100	12	121429321	protein_coding	rs3213572	12	121205078	7.61E-10	0.0226
LECT1	ENSG00000136110	13	53295673	protein_coding	rs1343605	13	53647048	6.23E-18	0.0226
AKAP5	ENSG00000179841	14	64934321	protein_coding	rs1152578	14	64697037	6.36E-10	0.0226
CDC42BPB	ENSG00000198752	14	103461257	protein_coding	rs10149470	14	104017953	3.72E-14	0.0226
CRYBA1	ENSG00000108255	17	27577694	protein_coding	rs75581564	17	27363750	3.17E-08	0.0226
CHD6	ENSG00000124177	20	40138937	protein_coding	rs143186028	20	39997404	2.29E-09	0.0226
PCIF1	ENSG00000100982	20	44569964	protein_coding	rs12624433	20	44680853	7.44E-09	0.0226
FBXO10	ENSG00000147912	9	37549880	protein_coding	rs7030813	9	36999369	3.07E-12	0.0214
TEKT2	ENSG00000092850	1	36551776	protein_coding	rs1002656	1	37192741	3.74E-12	0.0209
ZNF648	ENSG00000179930	1	182027276	protein_coding	rs169235	1	181740924	2.98E-08	0.0209
SACM1L	ENSG00000211456	3	45758724	protein_coding	rs4346585	3	44736493	7.13E-10	0.0209
ATRIP	ENSG00000164053	3	48497614	protein_coding	rs13084037	3	49214066	7.08E-09	0.0209
APEH	ENSG00000164062	3	49716415	protein_coding	rs13084037	3	49214066	7.08E-09	0.0209
MXD4	ENSG00000123933	4	2256590	protein_coding	rs7685686	4	3207142	2.57E-08	0.0209
QRFPR	ENSG00000186867	4	122276340	protein_coding	rs45510091	4	123186393	1.83E-08	0.0209
NUDT1	ENSG00000106268	7	2286319	protein_coding	rs3823624	7	2110346	1.99E-09	0.0209
SH3GL2	ENSG00000107295	9	17688040	protein_coding	rs263645	9	17016503	3.70E-10	0.0209

APLNR	ENSG00000134817	11	57002888	protein_coding	rs2509805	11	57650796	9.17E-09	0.0209
SSRP1	ENSG00000149136	11	57098405	protein_coding	rs2509805	11	57650796	9.17E-09	0.0209
SCGB1A1	ENSG00000149021	11	62181621	protein_coding	rs198457	11	61471678	2.99E-10	0.0209
CFL1	ENSG00000172757	11	65609995	protein_coding	rs58621819	11	65314830	1.57E-08	0.0209
FGF3	ENSG00000186895	11	69629392	protein_coding	rs7117514	11	70544937	7.29E-09	0.0209
NADSYN1	ENSG00000172890	11	71201691	protein_coding	rs7117514	11	70544937	7.29E-09	0.0209
CLDN25	ENSG00000228607	11	113650845	protein_coding	rs61902811	11	113370758	1.40E-12	0.0209
TMPRSS13	ENSG00000137747	11	117785766	protein_coding	rs2187490	11	118713180	3.82E-08	0.0209
DCPS	ENSG00000110063	11	126194645	protein_coding	rs57344483	11	127022560	1.82E-08	0.0209
SLC15A1	ENSG00000088386	13	99370903	protein_coding	rs4772087	13	99115041	3.91E-10	0.0209
FLVCR2	ENSG00000119686	14	76087258	protein_coding	rs1045430	14	75130235	7.31E-13	0.0209
UQCRC2	ENSG00000140740	16	21979481	protein_coding	rs12923444	16	21639710	1.30E-09	0.0209
RBFA	ENSG00000101546	18	77802498	protein_coding	rs7241572	18	77580712	2.70E-10	0.0209
CDH22	ENSG00000149654	20	44869754	protein_coding	rs12624433	20	44680853	7.44E-09	0.0209
RANGAP1	ENSG00000100401	22	41670289	protein_coding	rs5995992	22	41487218	1.30E-11	0.0209
PGA3	ENSG00000229859	11	60975674	protein_coding	rs198457	11	61471678	2.99E-10	0.0203
CACYBP	ENSG00000116161	1	174974575	protein_coding	rs10913112	1	175913828	3.40E-13	0.0192
TEDDM1	ENSG00000203730	1	182368501	protein_coding	rs169235	1	181740924	2.98E-08	0.0192
ST3GAL5	ENSG00000115525	2	86091202	protein_coding	rs7585722	2	86819128	2.68E-08	0.0192
ACTR8	ENSG00000113812	3	53908661	protein_coding	rs7624336	3	53244151	3.96E-08	0.0192
TMEM33	ENSG00000109133	4	41949863	protein_coding	rs34937911	4	42110353	4.13E-08	0.0192
SPATA4	ENSG00000150628	4	177111305	protein_coding	rs7659414	4	177350956	1.20E-08	0.0192
FRMPD1	ENSG00000070601	9	37698949	protein_coding	rs7030813	9	36999369	3.07E-12	0.0192
SLC25A51	ENSG00000122696	9	37891875	protein_coding	rs7030813	9	36999369	3.07E-12	0.0192
VPS37C	ENSG00000167987	11	60913408	protein_coding	rs198457	11	61471678	2.99E-10	0.0192
SYT7	ENSG00000011347	11	61315702	protein_coding	rs198457	11	61471678	2.99E-10	0.0192
DPP3	ENSG00000254986	11	66262307	protein_coding	rs58621819	11	65314830	1.57E-08	0.0192
FOXR1	ENSG00000176302	11	118847209	protein_coding	rs2187490	11	118713180	3.82E-08	0.0192
FOXRED1	ENSG00000110074	11	126143488	protein_coding	rs57344483	11	127022560	1.82E-08	0.0192
AREL1	ENSG00000119682	14	75149849	protein_coding	rs1045430	14	75130235	7.31E-13	0.0192
DHRS13	ENSG00000167536	17	27227444	protein_coding	rs75581564	17	27363750	3.17E-08	0.0192
EYA2	ENSG00000064655	20	45670377	protein_coding	rs12624433	20	44680853	7.44E-09	0.0192
NDUFA6	ENSG00000184983	22	42484244	protein_coding	rs5995992	22	41487218	1.30E-11	0.0192
SH3D21	ENSG00000214193	1	36781236	protein_coding	rs1002656	1	37192741	3.74E-12	0.0174

CDCP1	ENSG00000163814	3	45155842	protein_coding	rs4346585	3	44736493	7.13E-10	0.0174
LAMB2	ENSG00000172037	3	49164549	protein_coding	rs13084037	3	49214066	7.08E-09	0.0174
MST1	ENSG00000173531	3	49724157	protein_coding	rs13084037	3	49214066	7.08E-09	0.0174
CACNA1D	ENSG00000157388	3	53687586	protein_coding	rs7624336	3	53244151	3.96E-08	0.0174
PEX3	ENSG00000034693	6	143791835	protein_coding	rs2876520	6	142996618	2.29E-10	0.0174
P2RX3	ENSG00000109991	11	57121960	protein_coding	rs2509805	11	57650796	9.17E-09	0.0174
OR6Q1	ENSG00000172381	11	57798902	protein_coding	rs2509805	11	57650796	9.17E-09	0.0174
OR9Q2	ENSG00000186513	11	57958448	protein_coding	rs2509805	11	57650796	9.17E-09	0.0174
PRPF19	ENSG00000110107	11	60666131	protein_coding	rs198457	11	61471678	2.99E-10	0.0174
DDB1	ENSG00000167986	11	61088495	protein_coding	rs198457	11	61471678	2.99E-10	0.0174
FXYD6	ENSG00000137726	11	117727947	protein_coding	rs2187490	11	118713180	3.82E-08	0.0174
CXCR5	ENSG00000160683	11	118761491	protein_coding	rs2187490	11	118713180	3.82E-08	0.0174
H2AFX	ENSG00000188486	11	118965370	protein_coding	rs2187490	11	118713180	3.82E-08	0.0174
HINFP	ENSG00000172273	11	118999524	protein_coding	rs2187490	11	118713180	3.82E-08	0.0174
MTHFD1	ENSG00000100714	14	64890737	protein_coding	rs1152578	14	64697037	6.36E-10	0.0174
MAX	ENSG00000125952	14	65521152	protein_coding	rs1152578	14	64697037	6.36E-10	0.0174
YLPM1	ENSG00000119596	14	75267040	protein_coding	rs1045430	14	75130235	7.31E-13	0.0174
ACSM3	ENSG00000005187	16	20715234	protein_coding	rs12923444	16	21639710	1.30E-09	0.0174
LYRM1	ENSG00000102897	16	20923759	protein_coding	rs12923444	16	21639710	1.30E-09	0.0174
FOXN1	ENSG00000109101	17	26858436	protein_coding	rs75581564	17	27363750	3.17E-08	0.0174
STK4	ENSG00000101109	20	43651857	protein_coding	rs12624433	20	44680853	7.44E-09	0.0174
TOB2	ENSG00000183864	22	41836261	protein_coding	rs5995992	22	41487218	1.30E-11	0.0174
OTOA	ENSG00000155719	16	21730942	protein_coding	rs12923444	16	21639710	1.30E-09	0.0171
OR5B12	ENSG00000172362	11	58207119	protein_coding	rs2509805	11	57650796	9.17E-09	0.0164
BSN	ENSG00000164061	3	49650450	protein_coding	rs13084037	3	49214066	7.08E-09	0.0160
COL8A2	ENSG00000171812	1	36575829	protein_coding	rs1002656	1	37192741	3.74E-12	0.0157
SEC16B	ENSG00000254154	1	177952532	processed_transcript	rs72710803	1	177428018	5.29E-11	0.0157
SLC30A9	ENSG00000014824	4	42041020	protein_coding	rs34937911	4	42110353	4.13E-08	0.0157
AIG1	ENSG00000146416	6	143521537	protein_coding	rs2876520	6	142996618	2.29E-10	0.0157
MTFR1	ENSG00000066855	8	66619810	protein_coding	rs7837935	8	65562019	3.34E-09	0.0157
SHB	ENSG00000107338	9	37994170	protein_coding	rs7030813	9	36999369	3.07E-12	0.0157
STRBP	ENSG00000165209	9	125951317	protein_coding	rs2670139	9	126634255	1.21E-10	0.0157
SLC43A1	ENSG00000149150	11	57267633	protein_coding	rs2509805	11	57650796	9.17E-09	0.0157
OR9I1	ENSG00000172377	11	57886444	protein_coding	rs2509805	11	57650796	9.17E-09	0.0157

OR10Q1	ENSG00000180475	11	57995872	protein_coding	rs2509805	11	57650796	9.17E-09	0.0157
FADS3	ENSG00000221968	11	61650257	protein_coding	rs198457	11	61471678	2.99E-10	0.0157
BANF1	ENSG00000175334	11	65770585	protein_coding	rs58621819	11	65314830	1.57E-08	0.0157
AMICA1	ENSG00000160593	11	118080132	protein_coding	rs2187490	11	118713180	3.82E-08	0.0157
DPAGT1	ENSG00000172269	11	118973127	protein_coding	rs2187490	11	118713180	3.82E-08	0.0157
ABCG4	ENSG00000172350	11	119026541	protein_coding	rs2187490	11	118713180	3.82E-08	0.0157
USP2	ENSG00000036672	11	119239180	protein_coding	rs2187490	11	118713180	3.82E-08	0.0157
GLTP	ENSG00000139433	12	110303626	protein_coding	rs10774600	12	110741356	3.39E-08	0.0157
RXFP2	ENSG00000133105	13	32345341	protein_coding	rs1409379	13	31907741	1.67E-09	0.0157
GPX2	ENSG00000176153	14	65407700	protein_coding	rs1152578	14	64697037	6.36E-10	0.0157
EIF2B2	ENSG00000119718	14	75472953	protein_coding	rs1045430	14	75130235	7.31E-13	0.0157
MLH3	ENSG00000119684	14	75499351	protein_coding	rs1045430	14	75130235	7.31E-13	0.0157
DCUN1D3	ENSG00000188215	16	20890551	protein_coding	rs12923444	16	21639710	1.30E-09	0.0157
SGSM3	ENSG00000100359	22	40786358	protein_coding	rs5995992	22	41487218	1.30E-11	0.0157
SNIP1	ENSG00000163877	1	38011023	protein_coding	rs1466887	1	37709328	4.12E-08	0.0139
ZFYVE9	ENSG00000157077	1	52710202	protein_coding	rs1890946	1	52342427	2.68E-11	0.0139
GPX7	ENSG00000116157	1	53071383	protein_coding	rs1890946	1	52342427	2.68E-11	0.0139
USP39	ENSG00000168883	2	85853192	protein_coding	rs7585722	2	86819128	2.68E-08	0.0139
CPO	ENSG00000144410	2	207819238	protein_coding	rs62188629	2	208044470	7.13E-10	0.0139
PFKFB4	ENSG00000114268	3	48577282	protein_coding	rs13084037	3	49214066	7.08E-09	0.0139
IMPDH2	ENSG00000178035	3	49064256	protein_coding	rs13084037	3	49214066	7.08E-09	0.0139
USP19	ENSG00000172046	3	49151925	protein_coding	rs13084037	3	49214066	7.08E-09	0.0139
TRAIP	ENSG00000183763	3	49880020	protein_coding	rs13084037	3	49214066	7.08E-09	0.0139
MST1R	ENSG00000164078	3	49932867	protein_coding	rs13084037	3	49214066	7.08E-09	0.0139
NOP14	ENSG00000087269	4	2952386	protein_coding	rs7685686	4	3207142	2.57E-08	0.0139
SHISA3	ENSG00000178343	4	42402180	protein_coding	rs34937911	4	42110353	4.13E-08	0.0139
NEIL3	ENSG00000109674	4	178257543	protein_coding	rs7659414	4	177350956	1.20E-08	0.0139
FAM172A	ENSG00000113391	5	93200589	protein_coding	rs10061069	5	93071630	8.15E-11	0.0139
OR1S2	ENSG00000197887	11	57971163	protein_coding	rs2509805	11	57650796	9.17E-09	0.0139
DAK	ENSG00000149476	11	61110724	protein_coding	rs198457	11	61471678	2.99E-10	0.0139
CAPN1	ENSG00000014216	11	64963757	protein_coding	rs58621819	11	65314830	1.57E-08	0.0139
EHBP1L1	ENSG00000173442	11	65351815	protein_coding	rs58621819	11	65314830	1.57E-08	0.0139
ZW10	ENSG00000086827	11	113624221	protein_coding	rs61902811	11	113370758	1.40E-12	0.0139
CD3D	ENSG00000167286	11	118211564	protein_coding	rs2187490	11	118713180	3.82E-08	0.0139

PVRL1	ENSG00000110400	11	119546957	protein_coding	rs2187490	11	118713180	3.82E-08	0.0139
FRY	ENSG00000073910	13	32738115	protein_coding	rs1409379	13	31907741	1.67E-09	0.0139
THSD1	ENSG00000136114	13	52965967	protein_coding	rs1343605	13	53647048	6.23E-18	0.0139
NDFIP2	ENSG00000102471	13	80092748	protein_coding	rs9545360	13	80826373	5.02E-09	0.0139
SPTB	ENSG00000070182	14	65279801	protein_coding	rs1152578	14	64697037	6.36E-10	0.0139
LTBP2	ENSG00000119681	14	75022089	protein_coding	rs1045430	14	75130235	7.31E-13	0.0139
UNC119	ENSG00000109103	17	26876692	protein_coding	rs75581564	17	27363750	3.17E-08	0.0139
SLPI	ENSG00000124107	20	43882042	protein_coding	rs12624433	20	44680853	7.44E-09	0.0139
DNTTIP1	ENSG00000101457	20	44430321	protein_coding	rs12624433	20	44680853	7.44E-09	0.0139
TNNC2	ENSG00000101470	20	44457118	protein_coding	rs12624433	20	44680853	7.44E-09	0.0139
CDCA8	ENSG00000134690	1	38166740	protein_coding	rs1466887	1	37709328	4.12E-08	0.0122
YRDC	ENSG00000196449	1	38271236	protein_coding	rs1466887	1	37709328	4.12E-08	0.0122
CRB1	ENSG00000134376	1	197309088	protein_coding	rs17641524	1	197704717	1.52E-13	0.0122
IMMT	ENSG00000132305	2	86396974	protein_coding	rs7585722	2	86819128	2.68E-08	0.0122
EXOSC7	ENSG00000075914	3	45047145	protein_coding	rs4346585	3	44736493	7.13E-10	0.0122
P4HTM	ENSG00000178467	3	49035953	protein_coding	rs13084037	3	49214066	7.08E-09	0.0122
WDR6	ENSG00000178252	3	49048940	protein_coding	rs13084037	3	49214066	7.08E-09	0.0122
TMEM155	ENSG00000164112	4	122683335	protein_coding	rs45510091	4	123186393	1.83E-08	0.0122
BBS7	ENSG00000138686	4	122768623	protein_coding	rs45510091	4	123186393	1.83E-08	0.0122
LRRC70	ENSG00000186105	5	61875918	protein_coding	rs60157091	5	61509655	1.42E-08	0.0122
XKR9	ENSG00000221947	8	71642103	protein_coding	rs67436663	8	71347626	9.37E-10	0.0122
EXOSC3	ENSG00000107371	9	37784204	protein_coding	rs7030813	9	36999369	3.07E-12	0.0122
OR1S1	ENSG00000172774	11	57982706	protein_coding	rs2509805	11	57650796	9.17E-09	0.0122
MAP4K2	ENSG00000168067	11	64563501	protein_coding	rs58621819	11	65314830	1.57E-08	0.0122
MEN1	ENSG00000133895	11	64574877	protein_coding	rs58621819	11	65314830	1.57E-08	0.0122
CDC42BPG	ENSG00000171219	11	64601450	protein_coding	rs58621819	11	65314830	1.57E-08	0.0122
DPF2	ENSG00000133884	11	65110972	protein_coding	rs58621819	11	65314830	1.57E-08	0.0122
SLC25A45	ENSG00000162241	11	65146917	protein_coding	rs58621819	11	65314830	1.57E-08	0.0122
NLRX1	ENSG00000160703	11	119046001	protein_coding	rs2187490	11	118713180	3.82E-08	0.0122
TIRAP	ENSG00000150455	11	126160850	protein_coding	rs57344483	11	127022560	1.82E-08	0.0122
KCTD10	ENSG00000110906	12	109900905	protein_coding	rs10774600	12	110741356	3.39E-08	0.0122
PPP1CC	ENSG00000186298	12	111169114	protein_coding	rs10774600	12	110741356	3.39E-08	0.0122
SRSF9	ENSG00000111786	12	120903533	protein_coding	rs3213572	12	121205078	7.61E-10	0.0122
ANAPC5	ENSG00000089053	12	121791873	protein_coding	rs3213572	12	121205078	7.61E-10	0.0122

L3HYPDH	ENSG00000126790	14	59939114	protein_coding	rs1956373	14	60141822	2.06E-08	0.0122
ALDH6A1	ENSG00000119711	14	74537374	protein_coding	rs1045430	14	75130235	7.31E-13	0.0122
APOPT1	ENSG00000256053	14	104051579	protein_coding	rs10149470	14	104017953	3.72E-14	0.0122
SHISA9	ENSG00000237515	16	13164874	protein_coding	rs56887639	16	13755530	1.51E-12	0.0122
SUPT6H	ENSG00000109111	17	27009275	protein_coding	rs75581564	17	27363750	3.17E-08	0.0122
ERAL1	ENSG00000132591	17	27185052	protein_coding	rs75581564	17	27363750	3.17E-08	0.0122
POP4	ENSG00000105171	19	30102666	protein_coding	rs33431	19	30939989	4.81E-08	0.0122
ZNF334	ENSG00000198185	20	45135953	protein_coding	rs12624433	20	44680853	7.44E-09	0.0122
PMM1	ENSG00000100417	22	41979396	protein_coding	rs5995992	22	41487218	1.30E-11	0.0122
CENPM	ENSG00000100162	22	42338946	protein_coding	rs5995992	22	41487218	1.30E-11	0.0116
ACO2	ENSG00000100412	22	41895055	protein_coding	rs5995992	22	41487218	1.30E-11	0.0110
ENO1	ENSG00000074800	1	8930184	protein_coding	rs301799	1	8489302	1.36E-12	0.0105
AGO3	ENSG00000126070	1	36459191	protein_coding	rs1002656	1	37192741	3.74E-12	0.0105
OSBPL9	ENSG00000117859	1	52148870	protein_coding	rs1890946	1	52342427	2.68E-11	0.0105
INSL5	ENSG00000172410	1	67265181	protein_coding	rs10789214	1	67146817	4.44E-08	0.0105
ATOH8	ENSG00000168874	2	85996828	protein_coding	rs7585722	2	86819128	2.68E-08	0.0105
DYTN	ENSG00000232125	2	207549732	protein_coding	rs62188629	2	208044470	7.13E-10	0.0105
PLXNB1	ENSG00000164050	3	48458427	protein_coding	rs13084037	3	49214066	7.08E-09	0.0105
IP6K1	ENSG00000176095	3	49792851	protein_coding	rs13084037	3	49214066	7.08E-09	0.0105
MUSTN1	ENSG00000243696	3	52860788	protein_coding	rs7624336	3	53244151	3.96E-08	0.0105
VTA1	ENSG00000009844	6	142505226	protein_coding	rs2876520	6	142996618	2.29E-10	0.0105
ZFAND2A	ENSG00000178381	7	1196051	protein_coding	rs3823624	7	2110346	1.99E-09	0.0105
MICALL2	ENSG00000164877	7	1483619	protein_coding	rs3823624	7	2110346	1.99E-09	0.0105
TTYH3	ENSG00000136295	7	2688010	protein_coding	rs3823624	7	2110346	1.99E-09	0.0105
ZBTB6	ENSG00000186130	9	125672972	protein_coding	rs2670139	9	126634255	1.21E-10	0.0105
TMX2	ENSG00000213593	11	57494258	protein_coding	rs2509805	11	57650796	9.17E-09	0.0105
TMEM132A	ENSG00000006118	11	60698283	protein_coding	rs198457	11	61471678	2.99E-10	0.0105
SLC15A3	ENSG00000110446	11	60712279	protein_coding	rs198457	11	61471678	2.99E-10	0.0105
RAB3IL1	ENSG00000167994	11	61676257	protein_coding	rs198457	11	61471678	2.99E-10	0.0105
EEF1G	ENSG00000254772	11	62334737	protein_coding	rs198457	11	61471678	2.99E-10	0.0105
LTBP3	ENSG00000168056	11	65316216	protein_coding	rs58621819	11	65314830	1.57E-08	0.0105
KCNK7	ENSG00000173338	11	65361896	protein_coding	rs58621819	11	65314830	1.57E-08	0.0105
MUS81	ENSG00000172732	11	65629860	protein_coding	rs58621819	11	65314830	1.57E-08	0.0105
EFEMP2	ENSG00000172638	11	65637487	protein_coding	rs58621819	11	65314830	1.57E-08	0.0105

FOSL1	ENSG00000175592	11	65663767	protein_coding	rs58621819	11	65314830	1.57E-08	0.0105
TRIM49	ENSG00000204449	11	89785416	protein_coding	rs7932640	11	88744425	1.62E-15	0.0105
TMPRSS5	ENSG00000166682	11	113567683	protein_coding	rs61902811	11	113370758	1.40E-12	0.0105
HTR3A	ENSG00000166736	11	113853319	protein_coding	rs61902811	11	113370758	1.40E-12	0.0105
ANKRD13A	ENSG00000076513	12	110457279	protein_coding	rs10774600	12	110741356	3.39E-08	0.0105
PLA2G1B	ENSG00000170890	12	120762753	protein_coding	rs3213572	12	121205078	7.61E-10	0.0105
MEDAG	ENSG00000102802	13	31490018	protein_coding	rs1409379	13	31907741	1.67E-09	0.0105
IPO5	ENSG00000065150	13	98641146	protein_coding	rs4772087	13	99115041	3.91E-10	0.0105
UBAC2	ENSG00000134882	13	99945858	protein_coding	rs4772087	13	99115041	3.91E-10	0.0105
PCNXL4	ENSG00000126773	14	60597240	protein_coding	rs1956373	14	60141822	2.06E-08	0.0105
PTGR2	ENSG00000140043	14	74336038	protein_coding	rs1045430	14	75130235	7.31E-13	0.0105
LIN52	ENSG00000205659	14	74609717	protein_coding	rs1045430	14	75130235	7.31E-13	0.0105
ZFYVE21	ENSG00000100711	14	104191036	protein_coding	rs10149470	14	104017953	3.72E-14	0.0105
ASPG	ENSG00000166183	14	104565557	protein_coding	rs10149470	14	104017953	3.72E-14	0.0105
EEF2K	ENSG00000103319	16	22258078	protein_coding	rs12923444	16	21639710	1.30E-09	0.0105
NLK	ENSG00000087095	17	26446294	protein_coding	rs75581564	17	27363750	3.17E-08	0.0105
SARM1	ENSG00000004139	17	26709712	protein_coding	rs75581564	17	27363750	3.17E-08	0.0105
PIGS	ENSG00000087111	17	26889475	protein_coding	rs75581564	17	27363750	3.17E-08	0.0105
GIT1	ENSG00000108262	17	27908566	protein_coding	rs75581564	17	27363750	3.17E-08	0.0105
C18orf54	ENSG00000166845	18	51896679	protein_coding	rs12966052	18	52751639	1.25E-11	0.0105
VSTM2B	ENSG00000187135	19	30036358	protein_coding	rs33431	19	30939989	4.81E-08	0.0105
SREBF2	ENSG00000198911	22	42266210	protein_coding	rs5995992	22	41487218	1.30E-11	0.0105
MFSD10	ENSG00000109736	4	2934437	protein_coding	rs7685686	4	3207142	2.57E-08	0.0102
PARD6G	ENSG00000178184	18	77960272	protein_coding	rs7241572	18	77580712	2.70E-10	0.0091
OSCP1	ENSG00000116885	1	36898757	protein_coding	rs1466887	1	37709328	4.12E-08	0.0087
RGSL1	ENSG00000121446	1	182474495	protein_coding	rs169235	1	181740924	2.98E-08	0.0087
CFHR4	ENSG00000134365	1	196872493	protein_coding	rs17641524	1	197704717	1.52E-13	0.0087
ZNF852	ENSG00000178917	3	44546295	protein_coding	rs4346585	3	44736493	7.13E-10	0.0087
NME6	ENSG00000172113	3	48338964	protein_coding	rs13084037	3	49214066	7.08E-09	0.0087
NCKIPSD	ENSG00000213672	3	48712580	protein_coding	rs13084037	3	49214066	7.08E-09	0.0087
GRK4	ENSG00000125388	4	3003904	protein_coding	rs7685686	4	3207142	2.57E-08	0.0087
IL21	ENSG00000138684	4	123538003	protein_coding	rs45510091	4	123186393	1.83E-08	0.0087
BBS12	ENSG00000181004	4	123659977	protein_coding	rs45510091	4	123186393	1.83E-08	0.0087
ANKRD7	ENSG00000106013	7	117868756	protein_coding	rs7807677	7	117502574	1.82E-11	0.0087

RC3H2	ENSG00000056586	9	125637198	protein_coding	rs2670139	9	126634255	1.21E-10	0.0087
RABGAP1	ENSG00000011454	9	125785128	protein_coding	rs2670139	9	126634255	1.21E-10	0.0087
ELP4	ENSG00000109911	11	31668421	protein_coding	rs1448938	11	30892824	1.30E-09	0.0087
TIMM10	ENSG00000134809	11	57297106	protein_coding	rs2509805	11	57650796	9.17E-09	0.0087
SMTNL1	ENSG00000214872	11	57313543	protein_coding	rs2509805	11	57650796	9.17E-09	0.0087
YPEL4	ENSG00000166793	11	57414988	protein_coding	rs2509805	11	57650796	9.17E-09	0.0087
BATF2	ENSG00000168062	11	64759966	protein_coding	rs58621819	11	65314830	1.57E-08	0.0087
SART1	ENSG00000175467	11	65738229	protein_coding	rs58621819	11	65314830	1.57E-08	0.0087
USP28	ENSG00000048028	11	113707444	protein_coding	rs61902811	11	113370758	1.40E-12	0.0087
TMEM25	ENSG00000149582	11	118409875	protein_coding	rs2187490	11	118713180	3.82E-08	0.0087
UBE3B	ENSG00000151148	12	109944857	protein_coding	rs10774600	12	110741356	3.39E-08	0.0087
RAD9B	ENSG00000151164	12	110954675	protein_coding	rs10774600	12	110741356	3.39E-08	0.0087
RPLP0	ENSG00000089157	12	120636763	protein_coding	rs3213572	12	121205078	7.61E-10	0.0087
MLEC	ENSG00000110917	12	121132169	protein_coding	rs3213572	12	121205078	7.61E-10	0.0087
CKAP2	ENSG00000136108	13	53039990	protein_coding	rs1343605	13	53647048	6.23E-18	0.0087
ELMSAN1	ENSG00000156030	14	74219406	protein_coding	rs1045430	14	75130235	7.31E-13	0.0087
VSX2	ENSG00000119614	14	74717808	protein_coding	rs1045430	14	75130235	7.31E-13	0.0087
PGF	ENSG00000119630	14	75415512	protein_coding	rs1045430	14	75130235	7.31E-13	0.0087
TTLL5	ENSG00000119685	14	76260694	protein_coding	rs1045430	14	75130235	7.31E-13	0.0087
AMN	ENSG00000166126	14	103394463	protein_coding	rs10149470	14	104017953	3.72E-14	0.0087
TNFAIP2	ENSG00000185215	14	103596777	protein_coding	rs10149470	14	104017953	3.72E-14	0.0087
SEBOX	ENSG00000109072	17	26694331	protein_coding	rs75581564	17	27363750	3.17E-08	0.0087
PROCA1	ENSG00000167525	17	27034543	protein_coding	rs75581564	17	27363750	3.17E-08	0.0087
PIPOX	ENSG00000179761	17	27377076	protein_coding	rs75581564	17	27363750	3.17E-08	0.0087
DESI1	ENSG00000100418	22	42005566	protein_coding	rs5995992	22	41487218	1.30E-11	0.0087
MTA2	ENSG00000149480	11	62364999	protein_coding	rs198457	11	61471678	2.99E-10	0.0076
TNFRSF9	ENSG00000049249	1	7990416	protein_coding	rs301799	1	8489302	1.36E-12	0.0070
AGO1	ENSG00000092847	1	36365310	protein_coding	rs1002656	1	37192741	3.74E-12	0.0070
ADPRHL2	ENSG00000116863	1	36557004	protein_coding	rs1002656	1	37192741	3.74E-12	0.0070
TRAPPC3	ENSG00000054116	1	36608635	protein_coding	rs1002656	1	37192741	3.74E-12	0.0070
RNF11	ENSG00000123091	1	51720535	protein_coding	rs1890946	1	52342427	2.68E-11	0.0070
TTC39A	ENSG00000085831	1	51781859	protein_coding	rs1890946	1	52342427	2.68E-11	0.0070
ADAM23	ENSG00000114948	2	207397057	protein_coding	rs62188629	2	208044470	7.13E-10	0.0070
TMEM42	ENSG00000169964	3	44905261	protein_coding	rs4346585	3	44736493	7.13E-10	0.0070

ZDHHC3	ENSG00000163812	3	44987213	protein_coding	rs4346585	3	44736493	7.13E-10	0.0070
TMEM158	ENSG00000249992	3	45266864	protein_coding	rs4346585	3	44736493	7.13E-10	0.0070
CCDC51	ENSG00000164051	3	48477720	protein_coding	rs13084037	3	49214066	7.08E-09	0.0070
SHISA5	ENSG00000164054	3	48525728	protein_coding	rs13084037	3	49214066	7.08E-09	0.0070
AMT	ENSG00000145020	3	49457198	protein_coding	rs13084037	3	49214066	7.08E-09	0.0070
GMPPB	ENSG00000173540	3	49757830	protein_coding	rs13084037	3	49214066	7.08E-09	0.0070
CDHR4	ENSG00000187492	3	49832716	protein_coding	rs13084037	3	49214066	7.08E-09	0.0070
UBA7	ENSG00000182179	3	49847009	protein_coding	rs13084037	3	49214066	7.08E-09	0.0070
ALAS1	ENSG00000023330	3	52240222	protein_coding	rs7624336	3	53244151	3.96E-08	0.0070
SEMA3G	ENSG00000010319	3	52473085	protein_coding	rs7624336	3	53244151	3.96E-08	0.0070
RFT1	ENSG00000163933	3	53143488	protein_coding	rs7624336	3	53244151	3.96E-08	0.0070
NMBR	ENSG00000135577	6	142394701	protein_coding	rs2876520	6	142996618	2.29E-10	0.0070
C7orf50	ENSG00000146540	7	1107259	protein_coding	rs3823624	7	2110346	1.99E-09	0.0070
C7orf66	ENSG00000205174	7	108524338	protein_coding	rs58104186	7	109099919	1.82E-11	0.0070
ST7	ENSG00000004866	7	116731724	protein_coding	rs7807677	7	117502574	1.82E-11	0.0070
KCNV2	ENSG00000168263	9	2723638	protein_coding	rs1354115	9	2983774	7.08E-09	0.0070
GLIPR2	ENSG00000122694	9	36150320	protein_coding	rs7030813	9	36999369	3.07E-12	0.0070
C11orf31	ENSG00000211450	11	57509677	protein_coding	rs2509805	11	57650796	9.17E-09	0.0070
TMEM258	ENSG00000134825	11	61558354	protein_coding	rs198457	11	61471678	2.99E-10	0.0070
TUT1	ENSG00000149016	11	62351083	protein_coding	rs198457	11	61471678	2.99E-10	0.0070
EML3	ENSG00000149499	11	62374963	protein_coding	rs198457	11	61471678	2.99E-10	0.0070
NAALADL1	ENSG00000168060	11	64819155	protein_coding	rs58621819	11	65314830	1.57E-08	0.0070
DRAP1	ENSG00000175550	11	65687880	protein_coding	rs58621819	11	65314830	1.57E-08	0.0070
PACS1	ENSG00000175115	11	65925026	protein_coding	rs58621819	11	65314830	1.57E-08	0.0070
HTR3B	ENSG00000149305	11	113796343	protein_coding	rs61902811	11	113370758	1.40E-12	0.0070
UBE4A	ENSG00000110344	11	118250113	protein_coding	rs2187490	11	118713180	3.82E-08	0.0070
ARCN1	ENSG00000095139	11	118458417	protein_coding	rs2187490	11	118713180	3.82E-08	0.0070
TREH	ENSG00000118094	11	118539212	protein_coding	rs2187490	11	118713180	3.82E-08	0.0070
SLC37A4	ENSG00000137700	11	118898220	processed_transcript	rs2187490	11	118713180	3.82E-08	0.0070
HYOU1	ENSG00000149428	11	118921419	protein_coding	rs2187490	11	118713180	3.82E-08	0.0070
THY1	ENSG00000154096	11	119291892	protein_coding	rs2187490	11	118713180	3.82E-08	0.0070
TCHP	ENSG00000139437	12	110379857	protein_coding	rs10774600	12	110741356	3.39E-08	0.0070
HVCN1	ENSG00000122986	12	111104200	protein_coding	rs10774600	12	110741356	3.39E-08	0.0070
SPPL3	ENSG00000157837	12	121271243	protein_coding	rs3213572	12	121205078	7.61E-10	0.0070

RNF113B	ENSG00000139797	13	98828779	protein_coding	rs4772087	13	99115041	3.91E-10	0.0070
DHRS7	ENSG00000100612	14	60623706	protein_coding	rs1956373	14	60141822	2.06E-08	0.0070
SGPP1	ENSG00000126821	14	64172844	protein_coding	rs1152578	14	64697037	6.36E-10	0.0070
PNMA1	ENSG00000176903	14	74179811	protein_coding	rs1045430	14	75130235	7.31E-13	0.0070
ZC2HC1C	ENSG00000119703	14	75537999	protein_coding	rs1045430	14	75130235	7.31E-13	0.0070
C14orf2	ENSG00000156411	14	104386615	protein_coding	rs10149470	14	104017953	3.72E-14	0.0070
TNFAIP1	ENSG00000109079	17	26668382	protein_coding	rs75581564	17	27363750	3.17E-08	0.0070
TLCD1	ENSG00000160606	17	27052658	protein_coding	rs75581564	17	27363750	3.17E-08	0.0070
SYS1	ENSG00000204070	20	43998007	protein_coding	rs12624433	20	44680853	7.44E-09	0.0070
EPPIN	ENSG00000101448	20	44171008	protein_coding	rs12624433	20	44680853	7.44E-09	0.0070
SNX21	ENSG00000124104	20	44467181	protein_coding	rs12624433	20	44680853	7.44E-09	0.0070
SPATA25	ENSG00000149634	20	44515701	protein_coding	rs12624433	20	44680853	7.44E-09	0.0070
SLC35C2	ENSG00000080189	20	44985605	protein_coding	rs12624433	20	44680853	7.44E-09	0.0070
TP53RK	ENSG00000172315	20	45315711	protein_coding	rs12624433	20	44680853	7.44E-09	0.0070
ADSL	ENSG00000239900	22	40753665	protein_coding	rs5995992	22	41487218	1.30E-11	0.0070
ZC3H7B	ENSG00000100403	22	41726838	protein_coding	rs5995992	22	41487218	1.30E-11	0.0070
SIRT4	ENSG00000089163	12	120745585	protein_coding	rs3213572	12	121205078	7.61E-10	0.0061
GNA12	ENSG00000146535	7	2825852	protein_coding	rs3823624	7	2110346	1.99E-09	0.0060
EIF1AD	ENSG00000175376	11	65766831	protein_coding	rs58621819	11	65314830	1.57E-08	0.0059
SLC2A7	ENSG00000197241	1	9074881	protein_coding	rs301799	1	8489302	1.36E-12	0.0052
SPSB1	ENSG00000171621	1	9391265	protein_coding	rs301799	1	8489302	1.36E-12	0.0052
MRPS15	ENSG00000116898	1	36925678	protein_coding	rs1002656	1	37192741	3.74E-12	0.0052
MEAF6	ENSG00000163875	1	37969275	protein_coding	rs1466887	1	37709328	4.12E-08	0.0052
STX6	ENSG00000135823	1	180966954	protein_coding	rs169235	1	181740924	2.98E-08	0.0052
F13B	ENSG00000143278	1	197022359	protein_coding	rs17641524	1	197704717	1.52E-13	0.0052
PTCD3	ENSG00000132300	2	86351292	protein_coding	rs7585722	2	86819128	2.68E-08	0.0052
PLEKHM3	ENSG00000178385	2	208791655	protein_coding	rs62188629	2	208044470	7.13E-10	0.0052
ABHD5	ENSG00000011198	3	43753734	protein_coding	rs4346585	3	44736493	7.13E-10	0.0052
ZNF589	ENSG00000164048	3	48311666	protein_coding	rs13084037	3	49214066	7.08E-09	0.0052
UQCRC1	ENSG00000010256	3	48642422	protein_coding	rs13084037	3	49214066	7.08E-09	0.0052
SLC25A20	ENSG00000178537	3	48915397	protein_coding	rs13084037	3	49214066	7.08E-09	0.0052
DALRD3	ENSG00000178149	3	49056323	protein_coding	rs13084037	3	49214066	7.08E-09	0.0052
NDUFAF3	ENSG00000178057	3	49059398	protein_coding	rs13084037	3	49214066	7.08E-09	0.0052
NICN1	ENSG00000145029	3	49463569	protein_coding	rs13084037	3	49214066	7.08E-09	0.0052

TLR9	ENSG00000239732	3	52257637	protein_coding	rs7624336	3	53244151	3.96E-08	0.0052
PPM1M	ENSG00000164088	3	52282227	protein_coding	rs7624336	3	53244151	3.96E-08	0.0052
BAP1	ENSG00000163930	3	52439697	protein_coding	rs7624336	3	53244151	3.96E-08	0.0052
PHF7	ENSG00000010318	3	52450583	protein_coding	rs7624336	3	53244151	3.96E-08	0.0052
NEK4	ENSG00000114904	3	52774882	protein_coding	rs7624336	3	53244151	3.96E-08	0.0052
ITIH4	ENSG00000055955	3	52856243	protein_coding	rs7624336	3	53244151	3.96E-08	0.0052
HAUS3	ENSG00000214367	4	2236541	protein_coding	rs7685686	4	3207142	2.57E-08	0.0052
HGFAC	ENSG00000109758	4	3447412	protein_coding	rs7685686	4	3207142	2.57E-08	0.0052
LRPAP1	ENSG00000163956	4	3521194	protein_coding	rs7685686	4	3207142	2.57E-08	0.0052
MCHR2	ENSG00000152034	6	100404950	protein_coding	rs7758630	6	101387304	5.56E-10	0.0052
FUCA2	ENSG00000001036	6	143824720	protein_coding	rs2876520	6	142996618	2.29E-10	0.0052
BRAT1	ENSG00000106009	7	2586436	protein_coding	rs3823624	7	2110346	1.99E-09	0.0052
CCIN	ENSG00000185972	9	36170359	protein_coding	rs7030813	9	36999369	3.07E-12	0.0052
MPPED2	ENSG00000066382	11	30507229	protein_coding	rs1448938	11	30892824	1.30E-09	0.0052
IMMP1L	ENSG00000148950	11	31492570	protein_coding	rs1448938	11	30892824	1.30E-09	0.0052
MED19	ENSG00000156603	11	57475439	protein_coding	rs2509805	11	57650796	9.17E-09	0.0052
OR5B3	ENSG00000172769	11	58170409	protein_coding	rs2509805	11	57650796	9.17E-09	0.0052
OR5B2	ENSG00000172365	11	58190262	protein_coding	rs2509805	11	57650796	9.17E-09	0.0052
RASGRP2	ENSG00000068831	11	64503655	protein_coding	rs58621819	11	65314830	1.57E-08	0.0052
SF1	ENSG00000168066	11	64539168	protein_coding	rs58621819	11	65314830	1.57E-08	0.0052
PPP2R5B	ENSG00000068971	11	64693486	protein_coding	rs58621819	11	65314830	1.57E-08	0.0052
VPS51	ENSG00000149823	11	64868064	protein_coding	rs58621819	11	65314830	1.57E-08	0.0052
TIGD3	ENSG00000173825	11	65123661	protein_coding	rs58621819	11	65314830	1.57E-08	0.0052
RNASEH2C	ENSG00000172922	11	65485392	protein_coding	rs58621819	11	65314830	1.57E-08	0.0052
FIBP	ENSG00000172500	11	65653611	protein_coding	rs58621819	11	65314830	1.57E-08	0.0052
SF3B2	ENSG00000087365	11	65827489	protein_coding	rs58621819	11	65314830	1.57E-08	0.0052
NPAS4	ENSG00000174576	11	66191326	protein_coding	rs58621819	11	65314830	1.57E-08	0.0052
DEFB108B	ENSG00000184276	11	71546501	protein_coding	rs7117514	11	70544937	7.29E-09	0.0052
TRIM77	ENSG00000214414	11	89447253	pseudogene	rs7932640	11	88744425	1.62E-15	0.0052
MPZL3	ENSG00000160588	11	118110237	protein_coding	rs2187490	11	118713180	3.82E-08	0.0052
IFT46	ENSG00000118096	11	118429464	protein_coding	rs2187490	11	118713180	3.82E-08	0.0052
HMBS	ENSG00000256269	11	118959917	protein_coding	rs2187490	11	118713180	3.82E-08	0.0052
GPN3	ENSG00000111231	12	110898654	protein_coding	rs10774600	12	110741356	3.39E-08	0.0052
CIT	ENSG00000122966	12	120219345	protein_coding	rs3213572	12	121205078	7.61E-10	0.0052

RNF34	ENSG00000170633	12	121853116	protein_coding	rs3213572	12	121205078	7.61E-10	0.0052
RAP2A	ENSG00000125249	13	98103360	protein_coding	rs4772087	13	99115041	3.91E-10	0.0052
CHURC1	ENSG00000258289	14	65396194	protein_coding	rs1152578	14	64697037	6.36E-10	0.0052
NPC2	ENSG00000119655	14	74951887	protein_coding	rs1045430	14	75130235	7.31E-13	0.0052
ERI2	ENSG00000196678	16	20851593	protein_coding	rs12923444	16	21639710	1.30E-09	0.0052
ANKS4B	ENSG00000175311	16	21254368	protein_coding	rs12923444	16	21639710	1.30E-09	0.0052
TMEM97	ENSG00000109084	17	26650936	protein_coding	rs75581564	17	27363750	3.17E-08	0.0052
SPAG5	ENSG00000076382	17	26915452	protein_coding	rs75581564	17	27363750	3.17E-08	0.0052
TP53I13	ENSG00000167543	17	27897955	protein_coding	rs75581564	17	27363750	3.17E-08	0.0052
RBPJL	ENSG00000124232	20	43940647	protein_coding	rs12624433	20	44680853	7.44E-09	0.0052
OCSTAMP	ENSG00000149635	20	45174399	protein_coding	rs12624433	20	44680853	7.44E-09	0.0052
MCHR1	ENSG00000128285	22	41076786	protein_coding	rs5995992	22	41487218	1.30E-11	0.0052
PHF5A	ENSG00000100410	22	41860225	protein_coding	rs5995992	22	41487218	1.30E-11	0.0052
CSDC2	ENSG00000172346	22	42013382	protein_coding	rs5995992	22	41487218	1.30E-11	0.0052
NHP2L1	ENSG00000100138	22	42078221	protein_coding	rs5995992	22	41487218	1.30E-11	0.0052
CA6	ENSG00000131686	1	9020536	protein_coding	rs301799	1	8489302	1.36E-12	0.0035
SLC2A5	ENSG00000142583	1	9121851	protein_coding	rs301799	1	8489302	1.36E-12	0.0035
GPR157	ENSG00000180758	1	9174807	protein_coding	rs301799	1	8489302	1.36E-12	0.0035
EVA1B	ENSG00000142694	1	36788693	protein_coding	rs1002656	1	37192741	3.74E-12	0.0035
DNALI1	ENSG00000163879	1	38027489	protein_coding	rs1466887	1	37709328	4.12E-08	0.0035
GNL2	ENSG00000134697	1	38046978	protein_coding	rs1002656	1	37192741	3.74E-12	0.0035
KTI12	ENSG00000198841	1	52498631	protein_coding	rs1890946	1	52342427	2.68E-11	0.0035
PRPF38A	ENSG00000134748	1	52877114	protein_coding	rs1890946	1	52342427	2.68E-11	0.0035
SLC35D1	ENSG00000116704	1	67492398	protein_coding	rs10789214	1	67146817	4.44E-08	0.0035
VAMP5	ENSG00000168899	2	85816033	protein_coding	rs7585722	2	86819128	2.68E-08	0.0035
CRYGB	ENSG00000182187	2	209009094	protein_coding	rs62188629	2	208044470	7.13E-10	0.0035
TOPAZ1	ENSG00000173769	3	44328484	protein_coding	rs4346585	3	44736493	7.13E-10	0.0035
SPINK8	ENSG00000229453	3	48359083	protein_coding	rs13084037	3	49214066	7.08E-09	0.0035
DNAH1	ENSG00000114841	3	52392421	protein_coding	rs7624336	3	53244151	3.96E-08	0.0035
ITIH1	ENSG00000055957	3	52818840	protein_coding	rs7624336	3	53244151	3.96E-08	0.0035
ITIH3	ENSG00000162267	3	52835904	protein_coding	rs7624336	3	53244151	3.96E-08	0.0035
CACNA2D3	ENSG00000157445	3	54632579	protein_coding	rs7624336	3	53244151	3.96E-08	0.0035
ASB5	ENSG00000164122	4	177166773	protein_coding	rs7659414	4	177350956	1.20E-08	0.0035
RASA1	ENSG00000145715	5	86625925	protein_coding	rs3099439	5	87545318	5.05E-15	0.0035

TMEM161B	ENSG00000164180	5	87525371	protein_coding	rs3099439	5	87545318	5.05E-15	0.0035
TMEM184A	ENSG00000164855	7	1593126	protein_coding	rs3823624	7	2110346	1.99E-09	0.0035
ELFN1	ENSG00000225968	7	1757672	protein_coding	rs3823624	7	2110346	1.99E-09	0.0035
FTSJ2	ENSG00000122687	7	2277853	protein_coding	rs3823624	7	2110346	1.99E-09	0.0035
CARD11	ENSG00000198286	7	3014677	protein_coding	rs3823624	7	2110346	1.99E-09	0.0035
RNF38	ENSG00000137075	9	36411969	protein_coding	rs7030813	9	36999369	3.07E-12	0.0035
POLR1E	ENSG00000137054	9	37494813	protein_coding	rs7030813	9	36999369	3.07E-12	0.0035
GPR21	ENSG00000188394	9	125797390	protein_coding	rs2670139	9	126634255	1.21E-10	0.0035
IDI2	ENSG00000148377	10	1068323	protein_coding	rs997934	10	1795194	4.81E-08	0.0035
IDI1	ENSG00000067064	10	1090479	protein_coding	rs997934	10	1795194	4.81E-08	0.0035
DNAJC24	ENSG00000170946	11	31422391	protein_coding	rs1448938	11	30892824	1.30E-09	0.0035
OR5AK2	ENSG00000181273	11	56756844	protein_coding	rs2509805	11	57650796	9.17E-09	0.0035
LRRC55	ENSG00000183908	11	56954206	protein_coding	rs2509805	11	57650796	9.17E-09	0.0035
RTN4RL2	ENSG00000186907	11	57236514	protein_coding	rs2509805	11	57650796	9.17E-09	0.0035
OR5B17	ENSG00000197786	11	58126069	protein_coding	rs2509805	11	57650796	9.17E-09	0.0035
PGA5	ENSG00000256713	11	61013721	protein_coding	rs198457	11	61471678	2.99E-10	0.0035
TMEM216	ENSG00000187049	11	61162747	protein_coding	rs198457	11	61471678	2.99E-10	0.0035
SLC22A11	ENSG00000168065	11	64331722	protein_coding	rs58621819	11	65314830	1.57E-08	0.0035
PYGM	ENSG00000068976	11	64520815	protein_coding	rs58621819	11	65314830	1.57E-08	0.0035
ATG2A	ENSG00000110046	11	64673364	protein_coding	rs58621819	11	65314830	1.57E-08	0.0035
ZFPL1	ENSG00000162300	11	64853782	protein_coding	rs58621819	11	65314830	1.57E-08	0.0035
MRPL49	ENSG00000149792	11	64892249	protein_coding	rs58621819	11	65314830	1.57E-08	0.0035
SSSCA1	ENSG00000173465	11	65339657	protein_coding	rs58621819	11	65314830	1.57E-08	0.0035
CCDC85B	ENSG00000175602	11	65658490	protein_coding	rs58621819	11	65314830	1.57E-08	0.0035
RAB1B	ENSG00000174903	11	66040483	protein_coding	rs58621819	11	65314830	1.57E-08	0.0035
BRMS1	ENSG00000174744	11	66108700	protein_coding	rs58621819	11	65314830	1.57E-08	0.0035
PELI3	ENSG00000174516	11	66239512	protein_coding	rs58621819	11	65314830	1.57E-08	0.0035
ANKK1	ENSG00000170209	11	113264826	protein_coding	rs61902811	11	113370758	1.40E-12	0.0035
CD3G	ENSG00000160654	11	118220467	protein_coding	rs2187490	11	118713180	3.82E-08	0.0035
C2CD2L	ENSG00000172375	11	118981080	protein_coding	rs2187490	11	118713180	3.82E-08	0.0035
C1QTNF5	ENSG00000223953	11	119213517	protein_coding	rs2187490	11	118713180	3.82E-08	0.0035
MVK	ENSG00000110921	12	110023063	protein_coding	rs10774600	12	110741356	3.39E-08	0.0035
IFT81	ENSG00000122970	12	110609371	protein_coding	rs10774600	12	110741356	3.39E-08	0.0035
CCDC64	ENSG00000135127	12	120479973	protein_coding	rs3213572	12	121205078	7.61E-10	0.0035

GCN1L1	ENSG00000089154	12	120598760	protein_coding	rs3213572	12	121205078	7.61E-10	0.0035
GATC	ENSG00000111780	12	120886905	protein_coding	rs3213572	12	121205078	7.61E-10	0.0035
POP5	ENSG00000167272	12	121017884	protein_coding	rs3213572	12	121205078	7.61E-10	0.0035
UNC119B	ENSG00000175970	12	121154840	protein_coding	rs3213572	12	121205078	7.61E-10	0.0035
TMEM120B	ENSG00000188735	12	122185782	protein_coding	rs3213572	12	121205078	7.61E-10	0.0035
PPP2R5E	ENSG00000154001	14	63924083	protein_coding	rs1152578	14	64697037	6.36E-10	0.0035
FNTB	ENSG00000257365	14	65491403	protein_coding	rs1152578	14	64697037	6.36E-10	0.0035
SYNDIG1L	ENSG00000183379	14	74882700	protein_coding	rs1045430	14	75130235	7.31E-13	0.0035
TMCO5A	ENSG00000166069	15	38237032	protein_coding	rs8037355	15	37643831	3.94E-11	0.0035
IFT20	ENSG00000109083	17	26658924	protein_coding	rs75581564	17	27363750	3.17E-08	0.0035
KIAA0100	ENSG00000007202	17	26956965	protein_coding	rs75581564	17	27363750	3.17E-08	0.0035
STARD6	ENSG00000174448	18	51865953	protein_coding	rs62091461	18	52488672	1.95E-09	0.0035
SALL3	ENSG00000256463	18	76751476	protein_coding	rs7241572	18	77580712	2.70E-10	0.0035
KCNS1	ENSG00000124134	20	43725352	protein_coding	rs12624433	20	44680853	7.44E-09	0.0035
WFDC10B	ENSG00000182931	20	44323475	protein_coding	rs12624433	20	44680853	7.44E-09	0.0035
SPINT4	ENSG00000149651	20	44352729	protein_coding	rs12624433	20	44680853	7.44E-09	0.0035
ACOT8	ENSG00000101473	20	44478202	protein_coding	rs12624433	20	44680853	7.44E-09	0.0035
ZSWIM1	ENSG00000168612	20	44511885	protein_coding	rs12624433	20	44680853	7.44E-09	0.0035
NEURL2	ENSG00000124257	20	44518595	protein_coding	rs12624433	20	44680853	7.44E-09	0.0035
UTS2	ENSG00000049247	1	7908357	protein_coding	rs301799	1	8489302	1.36E-12	0.0017
EPHA10	ENSG00000183317	1	38205178	protein_coding	rs1002656	1	37192741	3.74E-12	0.0017
MANEAL	ENSG00000185090	1	38263141	protein_coding	rs1466887	1	37709328	4.12E-08	0.0017
TXNDC12	ENSG00000117862	1	52503823	protein_coding	rs1890946	1	52342427	2.68E-11	0.0017
FAM159A	ENSG00000182183	1	53117185	protein_coding	rs1890946	1	52342427	2.68E-11	0.0017
IL12RB2	ENSG00000081985	1	67817815	protein_coding	rs10789214	1	67146817	4.44E-08	0.0017
SERBP1	ENSG00000142864	1	67884795	protein_coding	rs10789214	1	67146817	4.44E-08	0.0017
MRPS14	ENSG00000120333	1	174986243	protein_coding	rs10913112	1	175913828	3.40E-13	0.0017
RGS8	ENSG00000135824	1	182634475	protein_coding	rs169235	1	181740924	2.98E-08	0.0017
CFH	ENSG00000000971	1	196668821	protein_coding	rs17641524	1	197704717	1.52E-13	0.0017
CFHR5	ENSG00000134389	1	196962735	protein_coding	rs17641524	1	197704717	1.52E-13	0.0017
ASPM	ENSG00000066279	1	197084541	protein_coding	rs17641524	1	197704717	1.52E-13	0.0017
RNF181	ENSG00000168894	2	85823792	protein_coding	rs7585722	2	86819128	2.68E-08	0.0017
MRPL35	ENSG00000132313	2	86433748	protein_coding	rs7585722	2	86819128	2.68E-08	0.0017
GPR1	ENSG00000183671	2	207061405	protein_coding	rs62188629	2	208044470	7.13E-10	0.0017

ZDBF2	ENSG00000204186	2	207159267	protein_coding	rs62188629	2	208044470	7.13E-10	0.0017
FASTKD2	ENSG00000118246	2	207643657	protein_coding	rs62188629	2	208044470	7.13E-10	0.0017
CRYGC	ENSG00000163254	2	208993707	protein_coding	rs62188629	2	208044470	7.13E-10	0.0017
CAMP	ENSG00000164047	3	48265909	protein_coding	rs13084037	3	49214066	7.08E-09	0.0017
TREX1	ENSG00000213689	3	48507744	protein_coding	rs13084037	3	49214066	7.08E-09	0.0017
SLC26A6	ENSG00000225697	3	48668041	protein_coding	rs13084037	3	49214066	7.08E-09	0.0017
ARIH2OS	ENSG00000221883	3	48956019	protein_coding	rs13084037	3	49214066	7.08E-09	0.0017
AMIGO3	ENSG00000176020	3	49757808	protein_coding	rs13084037	3	49214066	7.08E-09	0.0017
FAM212A	ENSG00000185614	3	49841575	protein_coding	rs13084037	3	49214066	7.08E-09	0.0017
SEMA3F	ENSG00000001617	3	50209493	protein_coding	rs13084037	3	49214066	7.08E-09	0.0017
TNNC1	ENSG00000114854	3	52486602	protein_coding	rs7624336	3	53244151	3.96E-08	0.0017
NT5DC2	ENSG00000168268	3	52563728	protein_coding	rs7624336	3	53244151	3.96E-08	0.0017
GLT8D1	ENSG00000016864	3	52734276	protein_coding	rs7624336	3	53244151	3.96E-08	0.0017
SPCS1	ENSG00000114902	3	52740576	protein_coding	rs7624336	3	53244151	3.96E-08	0.0017
IL17RB	ENSG00000056736	3	53890217	protein_coding	rs7624336	3	53244151	3.96E-08	0.0017
ADRA2C	ENSG00000184160	4	3769163	protein_coding	rs7685686	4	3207142	2.57E-08	0.0017
UNCX	ENSG00000164853	7	1274748	protein_coding	rs3823624	7	2110346	1.99E-09	0.0017
CHST12	ENSG00000136213	7	2458732	protein_coding	rs3823624	7	2110346	1.99E-09	0.0017
IQCE	ENSG00000106012	7	2626500	protein_coding	rs3823624	7	2110346	1.99E-09	0.0017
RECK	ENSG00000122707	9	36080439	protein_coding	rs7030813	9	36999369	3.07E-12	0.0017
CLTA	ENSG00000122705	9	36247815	protein_coding	rs7030813	9	36999369	3.07E-12	0.0017
DCAF10	ENSG00000122741	9	37834081	protein_coding	rs7030813	9	36999369	3.07E-12	0.0017
GPR144	ENSG00000180264	9	127227145	protein_coding	rs2670139	9	126634255	1.21E-10	0.0017
OLFML2A	ENSG00000185585	9	127558300	protein_coding	rs2670139	9	126634255	1.21E-10	0.0017
PRG2	ENSG00000186652	11	57156198	protein_coding	rs2509805	11	57650796	9.17E-09	0.0017
CNTF	ENSG00000242689	11	58391672	protein_coding	rs2509805	11	57650796	9.17E-09	0.0017
MS4A8	ENSG00000166959	11	60475165	protein_coding	rs198457	11	61471678	2.99E-10	0.0017
ZP1	ENSG00000149506	11	60639100	protein_coding	rs198457	11	61471678	2.99E-10	0.0017
VWCE	ENSG00000167992	11	61044329	protein_coding	rs198457	11	61471678	2.99E-10	0.0017
CYB561A3	ENSG00000162144	11	61122998	protein_coding	rs198457	11	61471678	2.99E-10	0.0017
TMEM138	ENSG00000149483	11	61133078	protein_coding	rs198457	11	61471678	2.99E-10	0.0017
PPP1R32	ENSG00000162148	11	61253497	protein_coding	rs198457	11	61471678	2.99E-10	0.0017
B3GAT3	ENSG00000149541	11	62386207	protein_coding	rs198457	11	61471678	2.99E-10	0.0017
UBXN1	ENSG00000162191	11	62445268	protein_coding	rs198457	11	61471678	2.99E-10	0.0017

ARL2	ENSG00000213465	11	64785620	protein_coding	rs58621819	11	65314830	1.57E-08	0.0017
SPDYC	ENSG00000204710	11	64939170	protein_coding	rs58621819	11	65314830	1.57E-08	0.0017
AP5B1	ENSG00000254470	11	65545818	protein_coding	rs58621819	11	65314830	1.57E-08	0.0017
CST6	ENSG00000175315	11	65780144	protein_coding	rs58621819	11	65314830	1.57E-08	0.0017
CATSPER1	ENSG00000175294	11	65789105	protein_coding	rs58621819	11	65314830	1.57E-08	0.0017
CNIH2	ENSG00000174871	11	66049216	protein_coding	rs58621819	11	65314830	1.57E-08	0.0017
YIF1A	ENSG00000174851	11	66054346	protein_coding	rs58621819	11	65314830	1.57E-08	0.0017
TRIM64	ENSG00000204450	11	89705034	pseudogene	rs7932640	11	88744425	1.62E-15	0.0017
TMPRSS4	ENSG00000137648	11	117970179	protein_coding	rs2187490	11	118713180	3.82E-08	0.0017
PDZD3	ENSG00000172367	11	119058549	protein_coding	rs2187490	11	118713180	3.82E-08	0.0017
MCAM	ENSG00000076706	11	119185520	protein_coding	rs2187490	11	118713180	3.82E-08	0.0017
MFRP	ENSG00000259159	11	119213510	protein_coding	rs2187490	11	118713180	3.82E-08	0.0017
MMAB	ENSG00000139428	12	110002467	protein_coding	rs10774600	12	110741356	3.39E-08	0.0017
RAB35	ENSG00000111737	12	120544102	protein_coding	rs3213572	12	121205078	7.61E-10	0.0017
TRIAP1	ENSG00000170855	12	120882989	protein_coding	rs3213572	12	121205078	7.61E-10	0.0017
RHOJ	ENSG00000126785	14	63715384	protein_coding	rs1152578	14	64697037	6.36E-10	0.0017
GPHB5	ENSG00000179600	14	63782571	lincRNA	rs1152578	14	64697037	6.36E-10	0.0017
PPP1R36	ENSG00000165807	14	65036359	protein_coding	rs1152578	14	64697037	6.36E-10	0.0017
PLEKHG3	ENSG00000126822	14	65192215	protein_coding	rs1152578	14	64697037	6.36E-10	0.0017
ENTPD5	ENSG00000187097	14	74455407	protein_coding	rs1045430	14	75130235	7.31E-13	0.0017
PROX2	ENSG00000119608	14	75325136	protein_coding	rs1045430	14	75130235	7.31E-13	0.0017
RPS6KL1	ENSG00000198208	14	75380378	protein_coding	rs1045430	14	75130235	7.31E-13	0.0017
ACYP1	ENSG00000119640	14	75528055	protein_coding	rs1045430	14	75130235	7.31E-13	0.0017
TRMT61A	ENSG00000166166	14	103999465	protein_coding	rs10149470	14	104017953	3.72E-14	0.0017
TMEM114	ENSG00000232258	16	8620903	protein_coding	rs7198928	16	7666402	4.45E-11	0.0017
ACSM1	ENSG00000166743	16	20672385	protein_coding	rs12923444	16	21639710	1.30E-09	0.0017
THUMPD1	ENSG00000066654	16	20749196	protein_coding	rs12923444	16	21639710	1.30E-09	0.0017
SDR42E2	ENSG00000183921	16	22185605	protein_coding	rs12923444	16	21639710	1.30E-09	0.0017
TMEM199	ENSG00000244045	17	26696681	protein_coding	rs75581564	17	27363750	3.17E-08	0.0017
VTN	ENSG00000255604	17	26697203	protein_coding	rs75581564	17	27363750	3.17E-08	0.0017
SDF2	ENSG00000132581	17	26982288	protein_coding	rs75581564	17	27363750	3.17E-08	0.0017
ABHD15	ENSG00000168792	17	27890866	protein_coding	rs75581564	17	27363750	3.17E-08	0.0017
CORO6	ENSG00000167549	17	27945845	protein_coding	rs75581564	17	27363750	3.17E-08	0.0017
EFCAB5	ENSG00000176927	17	28345844	protein_coding	rs75581564	17	27363750	3.17E-08	0.0017

WFDC5	ENSG00000175121	20	43740953	protein_coding	rs12624433	20	44680853	7.44E-09	0.0017
WFDC12	ENSG00000168703	20	43752586	protein_coding	rs12624433	20	44680853	7.44E-09	0.0017
SEMG1	ENSG00000124233	20	43837025	protein_coding	rs12624433	20	44680853	7.44E-09	0.0017
MATN4	ENSG00000124159	20	43929627	protein_coding	rs12624433	20	44680853	7.44E-09	0.0017
SDC4	ENSG00000124145	20	43965496	protein_coding	rs12624433	20	44680853	7.44E-09	0.0017
TP53TG5	ENSG00000124251	20	44019527	protein_coding	rs12624433	20	44680853	7.44E-09	0.0017
DBNDD2	ENSG00000244274	20	44036973	protein_coding	rs12624433	20	44680853	7.44E-09	0.0017
PIGT	ENSG00000124155	20	44049795	protein_coding	rs12624433	20	44680853	7.44E-09	0.0017
WFDC2	ENSG00000101443	20	44104259	protein_coding	rs12624433	20	44680853	7.44E-09	0.0017
WFDC6	ENSG00000243543	20	44165484	protein_coding	rs12624433	20	44680853	7.44E-09	0.0017
WFDC11	ENSG00000180083	20	44288055	protein_coding	rs12624433	20	44680853	7.44E-09	0.0017
WFDC13	ENSG00000168634	20	44334055	protein_coding	rs12624433	20	44680853	7.44E-09	0.0017
WFDC3	ENSG00000124116	20	44398577	protein_coding	rs12624433	20	44680853	7.44E-09	0.0017
CHADL	ENSG00000100399	22	41631227	protein_coding	rs5995992	22	41487218	1.30E-11	0.0017
POLR3H	ENSG00000100413	22	41931209	protein_coding	rs5995992	22	41487218	1.30E-11	0.0017
C22orf46	ENSG00000184208	22	42089541	protein_coding	rs5995992	22	41487218	1.30E-11	0.0017
CCDC134	ENSG00000100147	22	42209493	protein_coding	rs5995992	22	41487218	1.30E-11	0.0017
TNFRSF13(	ENSG00000159958	22	42321933	protein_coding	rs5995992	22	41487218	1.30E-11	0.0017
NAGA	ENSG00000198951	22	42447785	protein_coding	rs5995992	22	41487218	1.30E-11	0.0017
FAM109B	ENSG00000177096	22	42472850	protein_coding	rs5995992	22	41487218	1.30E-11	0.0017
FCF1	ENSG00000119616	14	75191620	protein_coding	rs1045430	14	75130235	7.31E-13	0.0001
H6PD	ENSG00000049239	1	9313129	protein_coding	rs301799	1	8489302	1.36E-12	0
AGO4	ENSG00000134698	1	36298632	protein_coding	rs1002656	1	37192741	3.74E-12	0
RSPO1	ENSG00000169218	1	38088773	protein_coding	rs1002656	1	37192741	3.74E-12	0
C1orf109	ENSG00000116922	1	38152578	protein_coding	rs1002656	1	37192741	3.74E-12	0
C1orf122	ENSG00000197982	1	38273888	protein_coding	rs1466887	1	37709328	4.12E-08	0
C1orf185	ENSG00000204006	1	51590829	protein_coding	rs1890946	1	52342427	2.68E-11	0
BTF3L4	ENSG00000134717	1	52539092	protein_coding	rs1890946	1	52342427	2.68E-11	0
COA7	ENSG00000162377	1	53158273	protein_coding	rs1890946	1	52342427	2.68E-11	0
ZYG11B	ENSG00000162378	1	53242570	protein_coding	rs1890946	1	52342427	2.68E-11	0
ZYG11A	ENSG00000203995	1	53334426	protein_coding	rs1890946	1	52342427	2.68E-11	0
TCTEX1D1	ENSG00000152760	1	67231306	protein_coding	rs10789214	1	67146817	4.44E-08	0
WDR78	ENSG00000152763	1	67334569	protein_coding	rs10789214	1	67146817	4.44E-08	0
C1orf141	ENSG00000203963	1	67579249	protein_coding	rs10789214	1	67146817	4.44E-08	0

IL23R	ENSG00000162594	1	67678872	protein_coding	rs10789214	1	67146817	4.44E-08	0
LRRIQ3	ENSG00000162620	1	74577785	protein_coding	rs10890020	1	73668836	4.03E-15	0
TNN	ENSG00000120332	1	175077098	protein_coding	rs10913112	1	175913828	3.40E-13	0
KIAA1614	ENSG00000135835	1	180901520	protein_coding	rs169235	1	181740924	2.98E-08	0
IER5	ENSG00000162783	1	181058807	protein_coding	rs169235	1	181740924	2.98E-08	0
CFHR3	ENSG00000116785	1	196754230	protein_coding	rs17641524	1	197704717	1.52E-13	0
CFHR1	ENSG00000244414	1	196795097	protein_coding	rs17641524	1	197704717	1.52E-13	0
CFHR2	ENSG00000080910	1	196858627	protein_coding	rs17641524	1	197704717	1.52E-13	0
C1orf53	ENSG00000203724	1	197874137	protein_coding	rs17641524	1	197704717	1.52E-13	0
TMEM150A	ENSG00000168890	2	85827995	protein_coding	rs7585722	2	86819128	2.68E-08	0
C2orf68	ENSG00000168887	2	85836483	protein_coding	rs7585722	2	86819128	2.68E-08	0
SFTPB	ENSG00000168878	2	85890150	protein_coding	rs7585722	2	86819128	2.68E-08	0
GNLY	ENSG00000115523	2	85919137	protein_coding	rs7585722	2	86819128	2.68E-08	0
RMND5A	ENSG00000153561	2	86976230	protein_coding	rs7585722	2	86819128	2.68E-08	0
RGPD1	ENSG00000187627	2	87188090	protein_coding	rs7585722	2	86819128	2.68E-08	0
PLGLB1	ENSG00000183281	2	87239328	protein_coding	rs7585722	2	86819128	2.68E-08	0
MDH1B	ENSG00000138400	2	207616379	protein_coding	rs62188629	2	208044470	7.13E-10	0
CCNYL1	ENSG00000163249	2	208601413	protein_coding	rs62188629	2	208044470	7.13E-10	0
CRYGD	ENSG00000118231	2	208987778	protein_coding	rs62188629	2	208044470	7.13E-10	0
CRYGA	ENSG00000168582	2	209026882	protein_coding	rs62188629	2	208044470	7.13E-10	0
C2orf80	ENSG00000188674	2	209042432	protein_coding	rs62188629	2	208044470	7.13E-10	0
TCAIM	ENSG00000179152	3	44415277	protein_coding	rs4346585	3	44736493	7.13E-10	0
KIAA1143	ENSG00000163807	3	44791153	protein_coding	rs4346585	3	44736493	7.13E-10	0
FBXW12	ENSG00000164049	3	48428187	protein_coding	rs13084037	3	49214066	7.08E-09	0
TMA7	ENSG00000232112	3	48483641	protein_coding	rs13084037	3	49214066	7.08E-09	0
UCN2	ENSG00000145040	3	48600183	protein_coding	rs13084037	3	49214066	7.08E-09	0
TMEM89	ENSG00000183396	3	48658740	protein_coding	rs13084037	3	49214066	7.08E-09	0
CELSR3	ENSG00000008300	3	48687125	protein_coding	rs13084037	3	49214066	7.08E-09	0
CCDC71	ENSG00000177352	3	49201861	protein_coding	rs13084037	3	49214066	7.08E-09	0
KLHDC8B	ENSG00000185909	3	49211481	protein_coding	rs13084037	3	49214066	7.08E-09	0
CCDC36	ENSG00000173421	3	49265699	protein_coding	rs13084037	3	49214066	7.08E-09	0
C3orf62	ENSG00000188315	3	49310688	protein_coding	rs13084037	3	49214066	7.08E-09	0
TCTA	ENSG00000145022	3	49451773	protein_coding	rs13084037	3	49214066	7.08E-09	0
CAMKV	ENSG00000164076	3	49901538	protein_coding	rs13084037	3	49214066	7.08E-09	0

MON1A	ENSG00000164077	3	49956954	protein_coding	rs13084037	3	49214066	7.08E-09	0
CHDH	ENSG00000016391	3	53865402	protein_coding	rs7624336	3	53244151	3.96E-08	0
C3orf55	ENSG00000174899	3	157328286	protein_coding	rs1095626	3	157977962	7.13E-14	0
IQCJ	ENSG00000214216	3	158832406	protein_coding	rs1095626	3	157977962	7.13E-14	0
FAM193A	ENSG00000125386	4	2680640	protein_coding	rs7685686	4	3207142	2.57E-08	0
MSANTD1	ENSG00000188981	4	3259780	protein_coding	rs7685686	4	3207142	2.57E-08	0
OTOP1	ENSG00000163982	4	4209573	protein_coding	rs7685686	4	3207142	2.57E-08	0
DCAF4L1	ENSG00000182308	4	41986094	protein_coding	rs34937911	4	42110353	4.13E-08	0
BEND4	ENSG00000188848	4	42133882	protein_coding	rs34937911	4	42110353	4.13E-08	0
GRXCR1	ENSG00000215203	4	42963979	protein_coding	rs34937911	4	42110353	4.13E-08	0
WDR17	ENSG00000150627	4	177045485	protein_coding	rs7659414	4	177350956	1.20E-08	0
KIAA0825	ENSG00000185261	5	93721490	protein_coding	rs10061069	5	93071630	8.15E-11	0
ADAT2	ENSG00000189007	6	143759444	protein_coding	rs2876520	6	142996618	2.29E-10	0
SAMD5	ENSG00000203727	6	147944373	protein_coding	rs725616	6	147950422	1.87E-08	0
C6orf118	ENSG00000112539	6	165708124	protein_coding	rs2029865	6	165121844	1.20E-08	0
PSMG3	ENSG00000157778	7	1608803	protein_coding	rs3823624	7	2110346	1.99E-09	0
GRIFIN	ENSG00000236734	7	2515444	lincRNA	rs3823624	7	2110346	1.99E-09	0
AMZ1	ENSG00000174945	7	2761957	protein_coding	rs3823624	7	2110346	1.99E-09	0
ARMC1	ENSG00000104442	8	66530568	protein_coding	rs7837935	8	65562019	3.34E-09	0
CCDC171	ENSG00000164989	9	15807278	protein_coding	rs263645	9	17016503	3.70E-10	0
C9orf92	ENSG00000205549	9	16240122	protein_coding	rs263645	9	17016503	3.70E-10	0
TUSC1	ENSG00000198680	9	25677626	protein_coding	rs59283172	9	25232978	1.02E-08	0
TOMM5	ENSG00000175768	9	37587641	protein_coding	rs7030813	9	36999369	3.07E-12	0
TRMT10B	ENSG00000165275	9	37766386	protein_coding	rs7030813	9	36999369	3.07E-12	0
ZBTB26	ENSG00000171448	9	125685812	protein_coding	rs2670139	9	126634255	1.21E-10	0
WDR38	ENSG00000136918	9	127617957	protein_coding	rs2670139	9	126634255	1.21E-10	0
WDR37	ENSG00000047056	10	1136857	protein_coding	rs997934	10	1795194	4.81E-08	0
KCNA4	ENSG00000182255	11	30034929	protein_coding	rs1448938	11	30892824	1.30E-09	0
DCDC1	ENSG00000188682	11	31178053	protein_coding	rs1448938	11	30892824	1.30E-09	0
PRG3	ENSG00000156575	11	57146432	protein_coding	rs2509805	11	57650796	9.17E-09	0
BTBD18	ENSG00000233436	11	57515121	protein_coding	rs2509805	11	57650796	9.17E-09	0
ZFP91	ENSG00000186660	11	58367549	protein_coding	rs2509805	11	57650796	9.17E-09	0
GLYAT	ENSG00000149124	11	58487992	protein_coding	rs2509805	11	57650796	9.17E-09	0
GLYATL2	ENSG00000156689	11	58636615	protein_coding	rs2509805	11	57650796	9.17E-09	0

MS4A15	ENSG00000166961	11	60534315	protein_coding	rs198457	11	61471678	2.99E-10	0
MS4A10	ENSG00000172689	11	60560799	protein_coding	rs198457	11	61471678	2.99E-10	0
PTGDR2	ENSG00000183134	11	60620928	protein_coding	rs198457	11	61471678	2.99E-10	0
PGA4	ENSG00000229183	11	61004308	protein_coding	rs198457	11	61471678	2.99E-10	0
LRRC10B	ENSG00000204950	11	61277377	protein_coding	rs198457	11	61471678	2.99E-10	0
SCGB1D1	ENSG00000168515	11	61959349	protein_coding	rs198457	11	61471678	2.99E-10	0
SCGB2A1	ENSG00000124939	11	61978774	protein_coding	rs198457	11	61471678	2.99E-10	0
SCGB1D2	ENSG00000124935	11	62010981	protein_coding	rs198457	11	61471678	2.99E-10	0
SCGB2A2	ENSG00000110484	11	62039127	protein_coding	rs198457	11	61471678	2.99E-10	0
SCGB1D4	ENSG00000197745	11	62065145	protein_coding	rs198457	11	61471678	2.99E-10	0
ROM1	ENSG00000149489	11	62380893	protein_coding	rs198457	11	61471678	2.99E-10	0
GANAB	ENSG00000089597	11	62403201	protein_coding	rs198457	11	61471678	2.99E-10	0
INTS5	ENSG00000185085	11	62417547	protein_coding	rs198457	11	61471678	2.99E-10	0
METTL12	ENSG00000214756	11	62434374	protein_coding	rs198457	11	61471678	2.99E-10	0
C11orf48	ENSG00000162194	11	62435007	protein_coding	rs198457	11	61471678	2.99E-10	0
LRRN4CL	ENSG00000177363	11	62455622	protein_coding	rs198457	11	61471678	2.99E-10	0
BSCL2	ENSG00000168000	11	62467532	protein_coding	rs198457	11	61471678	2.99E-10	0
SLC22A12	ENSG00000197891	11	64363966	protein_coding	rs58621819	11	65314830	1.57E-08	0
GPHA2	ENSG00000149735	11	64702651	protein_coding	rs58621819	11	65314830	1.57E-08	0
C11orf85	ENSG00000168070	11	64722273	protein_coding	rs58621819	11	65314830	1.57E-08	0
SNX15	ENSG00000110025	11	64794849	protein_coding	rs58621819	11	65314830	1.57E-08	0
SAC3D1	ENSG00000168061	11	64810336	protein_coding	rs58621819	11	65314830	1.57E-08	0
CDCA5	ENSG00000146670	11	64842704	protein_coding	rs58621819	11	65314830	1.57E-08	0
TM7SF2	ENSG00000149809	11	64881586	protein_coding	rs58621819	11	65314830	1.57E-08	0
ZNHIT2	ENSG00000174276	11	64884522	protein_coding	rs58621819	11	65314830	1.57E-08	0
SYVN1	ENSG00000162298	11	64895628	protein_coding	rs58621819	11	65314830	1.57E-08	0
POLA2	ENSG00000014138	11	65051146	protein_coding	rs58621819	11	65314830	1.57E-08	0
FRMD8	ENSG00000126391	11	65167533	protein_coding	rs58621819	11	65314830	1.57E-08	0
FAM89B	ENSG00000176973	11	65340744	protein_coding	rs58621819	11	65314830	1.57E-08	0
PCNXL3	ENSG00000197136	11	65394077	protein_coding	rs58621819	11	65314830	1.57E-08	0
OVOL1	ENSG00000172818	11	65559591	protein_coding	rs58621819	11	65314830	1.57E-08	0
SNX32	ENSG00000172803	11	65612739	protein_coding	rs58621819	11	65314830	1.57E-08	0
CTSW	ENSG00000172543	11	65649246	protein_coding	rs58621819	11	65314830	1.57E-08	0
C11orf68	ENSG00000175573	11	65685433	protein_coding	rs58621819	11	65314830	1.57E-08	0

GAL3ST3	ENSG00000175229	11	65813037	protein_coding	rs58621819	11	65314830	1.57E-08	0
KLC2	ENSG00000174996	11	66030048	protein_coding	rs58621819	11	65314830	1.57E-08	0
TMEM151A	ENSG00000179292	11	66061738	protein_coding	rs58621819	11	65314830	1.57E-08	0
CD248	ENSG00000174807	11	66083236	protein_coding	rs58621819	11	65314830	1.57E-08	0
RIN1	ENSG00000174791	11	66101012	protein_coding	rs58621819	11	65314830	1.57E-08	0
B3GNT1	ENSG00000174684	11	66114003	protein_coding	rs58621819	11	65314830	1.57E-08	0
SLC29A2	ENSG00000174669	11	66134838	protein_coding	rs58621819	11	65314830	1.57E-08	0
MRPL11	ENSG00000174547	11	66218377	protein_coding	rs58621819	11	65314830	1.57E-08	0
BBS1	ENSG00000174483	11	66289587	protein_coding	rs58621819	11	65314830	1.57E-08	0
ZDHHC24	ENSG00000174165	11	66300908	protein_coding	rs58621819	11	65314830	1.57E-08	0
ACTN3	ENSG00000248746	11	66322333	polymorphic_pseudogen	rs58621819	11	65314830	1.57E-08	0
FAM86C1	ENSG00000158483	11	71505419	protein_coding	rs7117514	11	70544937	7.29E-09	0
TTC12	ENSG00000149292	11	113219758	protein_coding	rs61902811	11	113370758	1.40E-12	0
C11orf71	ENSG00000180425	11	114266699	protein_coding	rs61902811	11	113370758	1.40E-12	0
RBM7	ENSG00000076053	11	114277838	protein_coding	rs61902811	11	113370758	1.40E-12	0
REXO2	ENSG00000076043	11	114315554	protein_coding	rs61902811	11	113370758	1.40E-12	0
SCN4B	ENSG00000177098	11	118013847	protein_coding	rs2187490	11	118713180	3.82E-08	0
SCN2B	ENSG00000149575	11	118040027	protein_coding	rs2187490	11	118713180	3.82E-08	0
MPZL2	ENSG00000149573	11	118129684	protein_coding	rs2187490	11	118713180	3.82E-08	0
ATP5L	ENSG00000167283	11	118287040	protein_coding	rs2187490	11	118713180	3.82E-08	0
TTC36	ENSG00000172425	11	118400049	protein_coding	rs2187490	11	118713180	3.82E-08	0
PHLDB1	ENSG00000019144	11	118502948	protein_coding	rs2187490	11	118713180	3.82E-08	0
CCDC84	ENSG00000186166	11	118877676	protein_coding	rs2187490	11	118713180	3.82E-08	0
TRAPPC4	ENSG00000196655	11	118892653	protein_coding	rs2187490	11	118713180	3.82E-08	0
CCDC153	ENSG00000248712	11	119063913	protein_coding	rs2187490	11	118713180	3.82E-08	0
RNF26	ENSG00000173456	11	119206630	protein_coding	rs2187490	11	118713180	3.82E-08	0
FAM118B	ENSG00000197798	11	126107095	protein_coding	rs57344483	11	127022560	1.82E-08	0
LRRIQ1	ENSG00000133640	12	85543547	protein_coding	rs56314503	12	84465022	2.95E-10	0
FAM222A	ENSG00000139438	12	110180172	protein_coding	rs10774600	12	110741356	3.39E-08	0
FAM216A	ENSG00000204856	12	110917179	protein_coding	rs10774600	12	110741356	3.39E-08	0
CCDC63	ENSG00000173093	12	111314956	protein_coding	rs10774600	12	110741356	3.39E-08	0
COX6A1	ENSG00000111775	12	120877219	protein_coding	rs3213572	12	121205078	7.61E-10	0
COQ5	ENSG00000110871	12	120956657	protein_coding	rs3213572	12	121205078	7.61E-10	0
C12orf43	ENSG00000157895	12	121447274	protein_coding	rs3213572	12	121205078	7.61E-10	0

MORN3	ENSG00000139714	12	122099780	protein_coding	rs3213572	12	121205078	7.61E-10	0
TEX26	ENSG00000175664	13	31528239	protein_coding	rs1409379	13	31907741	1.67E-09	0
ZAR1L	ENSG00000189167	13	32883659	protein_coding	rs1409379	13	31907741	1.67E-09	0
CCDC175	ENSG00000151838	14	60007675	protein_coding	rs1956373	14	60141822	2.06E-08	0
C14orf39	ENSG00000179008	14	60922724	protein_coding	rs1956373	14	60141822	2.06E-08	0
WDR89	ENSG00000140006	14	64086168	protein_coding	rs1152578	14	64697037	6.36E-10	0
RAB15	ENSG00000139998	14	65426013	protein_coding	rs1152578	14	64697037	6.36E-10	0
DNAL1	ENSG00000119661	14	74138591	protein_coding	rs1045430	14	75130235	7.31E-13	0
FAM161B	ENSG00000156050	14	74407660	protein_coding	rs1045430	14	75130235	7.31E-13	0
COQ6	ENSG00000119723	14	74423501	protein_coding	rs1045430	14	75130235	7.31E-13	0
CCDC176	ENSG00000119636	14	74517811	protein_coding	rs1045430	14	75130235	7.31E-13	0
VRTN	ENSG00000133980	14	74798241	protein_coding	rs1045430	14	75130235	7.31E-13	0
ISCA2	ENSG00000165898	14	74962116	protein_coding	rs1045430	14	75130235	7.31E-13	0
C14orf1	ENSG00000133935	14	76121833	protein_coding	rs1045430	14	75130235	7.31E-13	0
EXOC3L4	ENSG00000205436	14	103571688	protein_coding	rs10149470	14	104017953	3.72E-14	0
C15orf41	ENSG00000186073	15	36987130	protein_coding	rs8037355	15	37643831	3.94E-11	0
PDZD9	ENSG00000155714	16	22003809	protein_coding	rs12923444	16	21639710	1.30E-09	0
C16orf52	ENSG00000185716	16	22058907	protein_coding	rs12923444	16	21639710	1.30E-09	0
VWA3A	ENSG00000175267	16	22136073	protein_coding	rs12923444	16	21639710	1.30E-09	0
POLDIP2	ENSG00000004142	17	26679102	processed_transcript	rs75581564	17	27363750	3.17E-08	0
SLC13A2	ENSG00000007216	17	26812555	protein_coding	rs75581564	17	27363750	3.17E-08	0
ALDOC	ENSG00000109107	17	26902042	protein_coding	rs75581564	17	27363750	3.17E-08	0
FAM222B	ENSG00000173065	17	27126418	protein_coding	rs75581564	17	27363750	3.17E-08	0
ANKRD13B	ENSG00000198720	17	27929290	protein_coding	rs75581564	17	27363750	3.17E-08	0
KIAA1328	ENSG00000150477	18	34610275	protein_coding	rs12967855	18	35138245	1.18E-12	0
CCDC68	ENSG00000166510	18	52597739	protein_coding	rs12966052	18	52751639	1.25E-11	0
HSBP1L1	ENSG00000226742	18	77727701	protein_coding	rs7241572	18	77580712	2.70E-10	0
PI3	ENSG00000124102	20	43804351	protein_coding	rs12624433	20	44680853	7.44E-09	0
SEMG2	ENSG00000124157	20	43851520	protein_coding	rs12624433	20	44680853	7.44E-09	0
SPINT3	ENSG00000101446	20	44142682	protein_coding	rs12624433	20	44680853	7.44E-09	0
WFDC8	ENSG00000158901	20	44193878	protein_coding	rs12624433	20	44680853	7.44E-09	0
WFDC9	ENSG00000180205	20	44248242	protein_coding	rs12624433	20	44680853	7.44E-09	0
WFDC10A	ENSG00000180305	20	44259000	protein_coding	rs12624433	20	44680853	7.44E-09	0
ZSWIM3	ENSG00000132801	20	44497008	protein_coding	rs12624433	20	44680853	7.44E-09	0

PLTP	ENSG00000100979	20	44534096	protein_coding	rs12624433	20	44680853	7.44E-09	0
SLC13A3	ENSG00000158296	20	45245588	protein_coding	rs12624433	20	44680853	7.44E-09	0
DNAJB7	ENSG00000172404	22	41256841	protein_coding	rs5995992	22	41487218	1.30E-11	0
MEI1	ENSG00000167077	22	42145481	protein_coding	rs5995992	22	41487218	1.30E-11	0
SHISA8	ENSG00000234965	22	42308933	protein_coding	rs5995992	22	41487218	1.30E-11	0
WBP2NL	ENSG00000183066	22	42424594	protein_coding	rs5995992	22	41487218	1.30E-11	0
SMDT1	ENSG00000183172	22	42503978	protein_coding	rs5995992	22	41487218	1.30E-11	0

Table S2. DCC eQTL SNPs in fetal brains from the Human Developmental Biology Resource and their associations with depression							
**SNP_CHR**	**SNP_BP**	**SNP_rsID**	**DCC_eQTL_P**	**GWAS_A1**	**GWAS_A2**	**GWAS_LogOR**	**GWAS_P**
18	50776391	rs17410557	0.03891	t	c	-0.0267	1.42E-09
18	50731802	rs7227069	0.01004	a	g	0.0254	4.64E-09
18	50730524	rs62099234	0.01013	t	c	0.0253	5.09E-09
18	50730603	rs62099235	0.01013	a	g	0.0253	5.15E-09
18	50731055	rs62099236	0.01013	t	g	0.0252	6.05E-09
18	50739860	rs62100769	0.01003	a	g	0.0252	6.52E-09
18	50741297	rs62100772	0.01002	a	g	0.0252	6.56E-09
18	50740163	rs55906054	0.01003	a	g	-0.0251	6.63E-09
18	50739344	rs8086812	0.01004	c	g	-0.0251	6.80E-09
18	50726294	rs10221371	0.01013	a	c	0.0251	7.38E-09
18	50745236	rs11663824	0.02956	a	c	0.0253	7.92E-09
18	50729267	rs60939828	0.01013	t	c	-0.0247	1.14E-08
18	50718757	rs7232543	0.01151	a	g	-0.0246	1.28E-08
18	50614732	rs11663393	0.02603	a	g	0.0242	2.23E-08
18	50641696	rs5020052	0.00928	t	g	-0.024	2.52E-08
18	50620708	rs4459619	0.00626	t	c	-0.0239	2.87E-08
18	50633449	rs4494619	0.00625	t	g	-0.0239	2.93E-08
18	50718880	rs7233123	0.01666	a	g	0.024	3.04E-08
18	50622992	rs62099183	0.00625	t	c	-0.0239	3.05E-08
18	50749935	rs17488316	0.04151	a	t	-0.0236	4.67E-08
18	50747956	rs1367633	0.04129	a	g	0.0235	5.31E-08
18	50635096	rs4296324	0.02187	c	g	-0.0235	5.73E-08
18	50617175	rs4291980	0.02560	t	g	-0.0234	6.93E-08
18	50755757	rs62100777	0.04529	a	g	0.023	9.58E-08
18	50623640	rs4321259	0.01843	a	g	0.023	1.10E-07
18	50669725	rs8089828	0.01283	t	c	0.0227	1.35E-07
18	50672763	rs4939722	0.03100	t	c	0.0227	1.37E-07
18	50643812	rs4414555	0.00888	t	c	-0.0225	1.70E-07
18	50688080	rs8095327	0.04924	t	c	0.0225	1.96E-07
18	50611280	rs4468701	0.00731	t	c	-0.0223	2.47E-07
18	50611212	rs4368222	0.00729	c	g	0.0223	2.51E-07

18	50664582	rs8098809	0.03110	c	g	-0.0223	2.62E-07
18	50656126	rs7229097	0.03276	a	g	-0.0218	4.92E-07
18	50555931	rs62097947	0.03670	a	t	-0.0221	5.05E-07
18	50645664	rs4343352	0.03917	t	c	-0.0217	5.46E-07
18	50571954	rs11661483	0.03487	a	g	-0.0219	6.25E-07
18	50728987	rs11662175	0.03188	t	c	-0.0214	6.55E-07
18	50570431	rs7506451	0.03482	a	t	0.0218	6.74E-07
18	50570727	rs7506328	0.03508	a	t	-0.0217	7.35E-07
18	50559986	rs11663694	0.03455	t	c	-0.0218	7.47E-07
18	50573936	rs62097950	0.03453	t	c	0.0217	7.47E-07
18	50597654	rs4506980	0.03266	a	g	0.0217	8.12E-07
18	50570403	rs7506245	0.03514	a	g	-0.0216	8.52E-07
18	50595936	rs62099173	0.03221	t	g	-0.0216	9.01E-07
18	50597737	rs4510098	0.03269	t	g	0.0215	9.85E-07
18	50589083	rs4129439	0.03377	t	c	-0.0215	9.87E-07
18	50660450	rs4445960	0.04635	a	g	-0.0197	4.85E-06
18	50656120	rs7228703	0.04631	a	c	0.0195	6.16E-06
18	50642307	rs12326559	0.04616	a	g	0.0195	6.18E-06
18	50627680	rs9950661	0.04626	t	c	-0.0194	6.71E-06
18	50639540	rs8088927	0.04633	a	g	0.0192	7.90E-06
18	50629249	rs17393029	0.04625	a	t	0.0192	8.47E-06
18	50652265	rs8083043	0.04612	a	g	-0.0188	1.34E-05
18	50671627	rs4939721	0.03433	t	g	0.0186	1.55E-05
18	50652346	rs8083977	0.04636	t	c	0.0186	1.69E-05
18	50607242	rs72917329	0.03076	a	g	0.0183	3.39E-05
18	50552965	rs34569535	0.04031	a	c	-0.0176	6.19E-05
18	50517509	rs62097899	0.04976	t	c	0.017	9.33E-05
18	50622242	rs8082811	0.00482	a	g	0.0219	0.000107
18	50621455	rs10502963	0.00482	t	c	0.0218	0.000114
18	50622457	rs8083463	0.00482	a	g	-0.0219	0.000119
18	50622104	rs34006123	0.00482	t	c	-0.0216	0.000135
18	50547499	rs9807185	0.03778	t	g	0.0166	0.000137
18	50622374	rs56173310	0.01720	a	g	0.0215	0.000147
18	50618359	rs7233997	0.02206	a	g	-0.0214	0.000158

18	50543302	rs56796226	0.03756	a	g	-0.0164	0.000160
18	50622885	rs16956110	0.00481	t	c	-0.0213	0.000163
18	50621338	rs12607886	0.00482	t	c	-0.0213	0.000167
18	50621755	rs12605900	0.00482	a	t	-0.0213	0.000167
18	50622068	rs34540811	0.00482	t	c	-0.0213	0.000168
18	50622354	rs35946824	0.00482	c	g	-0.0212	0.000173
18	50531232	rs62097903	0.04598	a	g	-0.0164	0.000175
18	50622550	rs8084433	0.00482	a	t	-0.0212	0.000178
18	50622857	rs9957443	0.00482	t	g	0.0212	0.000181
18	50621987	rs34095568	0.00482	t	c	-0.0211	0.000184
18	50536188	rs62097940	0.03745	c	g	-0.0162	0.000194
18	50622015	rs35585120	0.00482	a	c	0.0208	0.000231
18	50671452	rs28572000	0.01552	t	g	-0.0207	0.000351
18	50670684	rs7238685	0.00813	a	g	-0.0203	0.000441
18	50668321	rs9956477	0.00812	a	c	-0.0202	0.000479
18	50669784	rs8088857	0.00406	t	c	-0.0196	0.000790
18	50595418	rs9947923	0.03009	c	g	-0.017	0.000926
18	50653472	rs35594898	0.00814	a	t	-0.0194	0.000926
18	50653816	rs35458640	0.00814	a	g	-0.0194	0.000926
18	50594568	rs8082863	0.04688	c	g	0.017	0.000933
18	50706247	rs10775504	0.02735	a	g	-0.0142	0.00103
18	50596618	rs9963521	0.02418	t	c	0.0168	0.00127
18	50593115	rs8094435	0.03002	a	g	0.0165	0.00132
18	50589376	rs8084270	0.02371	a	g	-0.0163	0.00152
18	50606585	rs12327491	0.03868	a	g	-0.0164	0.00155
18	50588945	rs8084454	0.03770	a	t	-0.0163	0.00156
18	50603493	rs12606343	0.03550	a	c	0.0162	0.00170
18	50602476	rs6508195	0.03417	t	c	-0.0162	0.00175
18	50350050	rs1835711	0.01456	a	g	0.0209	0.00184
18	50350059	rs1835710	0.01456	t	c	0.0209	0.00192
18	50576410	rs7244694	0.04502	t	c	0.0158	0.00198
18	50349934	rs5008899	0.03245	a	g	0.0207	0.00203
18	50585438	rs8099235	0.04391	c	g	-0.0156	0.00228
18	50595725	rs9947974	0.03015	a	g	0.0157	0.00236

18	50585467	rs6508194	0.04383	c	g	-0.0156	0.00237
18	50585582	rs7226789	0.04386	t	g	0.0155	0.00241
18	50514384	rs8088528	0.01606	a	g	-0.0132	0.00250
18	50614805	rs113034611	0.03238	a	t	0.0179	0.00342
18	50526501	rs10775503	0.01103	t	c	0.0124	0.00434
18	50521436	rs9807752	0.00795	a	t	-0.0121	0.00545
18	50682301	rs7226725	0.02340	a	g	-0.017	0.00708
18	50678942	rs11877180	0.02325	t	c	0.0169	0.00738
18	50677256	rs8083973	0.01359	a	g	0.0167	0.00829
18	50666143	rs72921429	0.01364	a	g	-0.0166	0.00845
18	50649171	rs17406589	0.01330	a	g	-0.0166	0.00878
18	50623189	rs16956114	0.01230	a	g	-0.0163	0.00888
18	50664446	rs8098423	0.01331	t	c	-0.0165	0.00900
18	50656054	rs11875455	0.01347	a	g	0.0165	0.00920
18	50631316	rs17484212	0.01346	a	g	-0.0164	0.00929
18	50618245	rs112866638	0.01792	a	c	0.0164	0.00933
18	50636267	rs17393231	0.01346	t	g	0.0162	0.0102
18	50635363	rs72919482	0.01346	t	c	-0.0162	0.0104
18	50670111	rs8092993	0.01233	a	g	-0.0162	0.0104
18	50670963	rs58142657	0.01233	c	g	0.0161	0.0109
18	50599751	rs60638615	0.01230	t	c	-0.0159	0.0109
18	50664079	rs4273117	0.01234	a	g	-0.016	0.0111
18	50599812	rs60237858	0.01231	a	g	0.0158	0.0115
18	50627322	rs11876405	0.01232	t	c	-0.016	0.0116
18	50636674	rs7235942	0.01232	a	g	0.0159	0.0118
18	50641515	rs8089823	0.01232	t	c	-0.0159	0.0121
18	50636319	rs7236657	0.01445	t	g	0.0159	0.0121
18	50657106	rs34556005	0.01233	a	c	-0.0158	0.0121
18	50642745	rs8094882	0.01232	a	t	0.0158	0.0122
18	50657150	rs56393416	0.01233	t	c	-0.0158	0.0125
18	50654992	rs8096803	0.02720	a	t	0.0158	0.0126
18	50627222	rs11873586	0.01232	t	g	0.0158	0.0126
18	50627016	rs72919449	0.01232	a	g	0.0158	0.0126
18	50636373	rs7235602	0.01233	t	c	-0.0158	0.0127

18	50650591	rs8083111	0.02718	t	c	0.0157	0.0128
18	50634839	rs11082966	0.01232	t	c	0.0157	0.0129
18	50657570	rs11873641	0.02721	t	c	0.0157	0.0129
18	50634290	rs8098518	0.01232	a	g	-0.0157	0.0131
18	50636909	rs11082968	0.01232	t	c	-0.0156	0.0133
18	50616180	rs56287586	0.01231	a	g	0.0156	0.0134
18	50616119	rs56229265	0.01231	a	g	0.0156	0.0136
18	50609008	rs72917331	0.03356	a	g	0.0156	0.0137
18	50647667	rs11872442	0.01542	a	t	0.0156	0.0140
18	50639275	rs7233664	0.02720	t	g	0.0155	0.0141
18	50640427	rs57863253	0.02720	a	g	-0.0155	0.0144
18	50622714	rs60830517	0.01230	t	g	-0.0154	0.0151
18	50605990	rs72917327	0.01344	a	c	-0.0152	0.0164
18	50423329	rs7237177	0.00825	c	g	-0.0158	0.0164
18	50623957	rs7238021	0.01230	a	t	-0.0151	0.0165
18	50429920	rs8091553	0.03024	c	g	-0.0184	0.0196
18	50634905	rs11082967	0.01105	a	g	-0.0145	0.0196
18	50425132	rs1943107	0.00810	a	g	0.0152	0.0203
18	50438655	rs4401134	0.00824	t	c	-0.0152	0.0211
18	50605182	rs72917325	0.01231	a	c	-0.0145	0.0214
18	50505640	rs9965121	0.01524	a	g	0.0122	0.0215
18	50540352	rs58427689	0.00637	t	c	0.0099	0.0219
18	50429238	rs77673472	0.00795	a	g	0.0151	0.0220
18	50425413	rs74502735	0.00809	a	c	-0.0149	0.0233
18	50448235	rs16955848	0.01944	t	c	-0.0178	0.0247
18	50164676	rs148273607	0.01364	a	c	-0.0138	0.0264
18	50536108	rs62097939	0.00210	a	g	-0.0093	0.0306
18	49631847	rs8084161	0.03829	t	c	0.0096	0.0306
18	50484996	rs1893569	0.00244	a	g	-0.0115	0.0309
18	50492071	rs11082959	0.00812	t	c	0.0114	0.0323
18	50490837	rs35196449	0.00811	t	c	-0.0114	0.0327
18	49634581	rs11662078	0.04324	t	c	0.0094	0.0334
18	50529659	rs7504478	0.00287	t	c	0.0091	0.0340
18	50349570	rs75807737	0.01775	a	g	-0.0153	0.0351

18	50174859	rs28516859	0.02692	a	g	0.013	0.0371
18	50349401	rs9956101	0.04113	t	c	-0.0149	0.0394
18	49630717	rs11660276	0.03520	t	c	-0.0091	0.0402
18	50544871	rs7505465	0.00988	t	c	-0.0089	0.0409
18	49630658	rs11663291	0.03069	a	g	-0.009	0.0416
18	50348576	rs12605728	0.04116	a	g	-0.0144	0.0459
18	50467040	rs28403887	0.00759	t	g	0.0107	0.0465
18	50348383	rs12605630	0.04318	a	t	0.0142	0.0498
18	50348095	rs9964788	0.04502	t	g	0.0141	0.0504
18	50347682	rs2082290	0.04766	t	c	-0.0141	0.0506
18	49266686	rs117394130	0.02520	a	c	0.0157	0.0708
18	50483333	rs67973712	0.01314	c	g	0.028	0.0768
18	50510230	rs72928193	0.04264	t	c	0.0277	0.0774
18	50506591	rs66804102	0.03158	a	c	-0.0276	0.0808
18	50506608	rs67300728	0.03160	a	g	0.0275	0.0819
18	50534395	rs55749022	0.03820	c	g	-0.0076	0.0842
18	50511324	rs67230963	0.03165	a	c	0.0272	0.0847
18	50507187	rs67539636	0.04262	a	c	0.0269	0.0868
18	49728512	rs9951603	0.04117	a	t	0.0096	0.0868
18	50499419	rs56888517	0.04249	a	g	0.0265	0.0897
18	49728514	rs9951824	0.04120	t	g	-0.0095	0.0899
18	50229634	rs10163674	0.03698	t	c	-0.0087	0.0931
18	50505739	rs67330260	0.03278	a	g	0.0262	0.0957
18	50503853	rs8086856	0.03278	t	c	-0.0263	0.0958
18	50470258	rs2156287	0.01418	t	c	0.0259	0.0958
18	50529171	rs9945571	0.03167	t	g	-0.0261	0.0965
18	50553133	rs67277226	0.03944	a	g	0.0266	0.0967
18	50515871	rs67211888	0.03166	t	c	-0.0249	0.1016
18	50515664	rs111244708	0.03236	a	g	-0.0254	0.1079
18	50512991	rs59456053	0.03168	a	g	-0.025	0.1133
18	50542535	rs72932047	0.03677	a	g	-0.025	0.1143
18	50527622	rs9962352	0.03247	a	g	0.0246	0.1163
18	50518567	rs58735160	0.03166	a	g	-0.0247	0.1176
18	50540018	rs113956550	0.03586	t	c	-0.0247	0.1176

18	50533785	rs56005836	0.03346	t	c	-0.0239	0.1305
18	50524139	rs28569227	0.03406	a	g	-0.0235	0.1354
18	49341470	rs7239900	0.01026	a	g	-0.0075	0.1397
18	50495028	rs56071382	0.03277	t	c	0.0232	0.1401
18	49303801	rs12604954	0.00495	t	c	0.0076	0.1451
18	50068475	rs1145273	0.02566	t	c	0.0135	0.1573
18	49186917	rs12608309	0.00591	t	c	0.0101	0.1682
18	49304611	rs975733	0.00709	t	c	-0.0072	0.1684
18	49302211	rs8083614	0.00497	a	g	-0.0072	0.1695
18	49434028	rs2928932	0.03569	a	c	0.0062	0.1697
18	49316854	rs1354477	0.00515	a	g	0.0072	0.17
18	49318430	rs7240067	0.00531	a	g	0.0072	0.1704
18	50049731	rs1145263	0.02135	t	g	0.0132	0.1705
18	50742384	rs12964204	0.00723	a	g	-0.0093	0.1723
18	50049824	rs1145264	0.02135	c	g	-0.0131	0.1731
18	50540364	rs67744445	0.03620	a	g	-0.0212	0.1748
18	50070167	rs1144044	0.02566	t	c	0.0129	0.1775
18	50743414	rs7243444	0.00722	a	g	-0.0091	0.1814
18	50742520	rs9951763	0.00723	a	g	-0.0091	0.1822
18	50743518	rs7243345	0.00722	t	c	-0.009	0.1875
18	50740781	rs7237256	0.00723	a	g	-0.0089	0.1899
18	49313585	rs1504508	0.00503	t	c	-0.0068	0.1925
18	49956578	rs11665179	0.03983	a	g	-0.009	0.1963
18	49302397	rs2134894	0.03500	a	g	0.0069	0.1981
18	50175582	rs112596036	0.01278	a	t	0.0093	0.2018
18	50160781	rs79851445	0.00616	a	g	0.007	0.2085
18	49298415	rs9949657	0.00607	t	c	0.0065	0.2106
18	50163614	rs4575625	0.01046	t	c	0.0095	0.2109
18	49462766	rs2953262	0.02518	a	c	0.0054	0.2159
18	49459814	rs2928945	0.02592	a	g	0.0053	0.2216
18	49461960	rs2953263	0.02529	t	c	-0.0053	0.2225
18	49313109	rs6508088	0.00502	a	t	-0.0063	0.2275
18	49447240	rs72918662	0.03931	a	g	0.0093	0.2294
18	49287303	rs1857732	0.04807	t	c	-0.0062	0.2317

18	50106919	rs72912995	0.04974	a	c	0.0085	0.2332
18	49450305	rs11872593	0.02786	a	c	-0.0092	0.2334
18	49311479	rs7240494	0.00475	a	g	0.0062	0.2337
18	49444526	rs72918659	0.03066	a	g	-0.0092	0.2357
18	50744585	rs12956682	0.02824	t	g	-0.0082	0.2363
18	50757622	rs72923285	0.03309	t	c	-0.0113	0.2452
18	50166147	rs75080518	0.01100	t	c	-0.0087	0.2475
18	49460119	rs2953264	0.02496	a	t	-0.005	0.2501
18	49336877	rs822067	0.00535	a	g	0.006	0.2519
18	49282969	rs323117	0.04752	a	c	-0.0058	0.2596
18	50720363	rs8097377	0.01135	a	t	0.0078	0.26
18	49254615	rs323076	0.04982	a	g	-0.0072	0.2674
18	49242852	rs2615547	0.04584	t	c	0.0072	0.268
18	50190888	rs56125618	0.00951	t	g	0.0066	0.2706
18	49251473	rs1676109	0.02151	a	g	-0.0072	0.2707
18	50188520	rs11082933	0.00949	c	g	0.0066	0.2713
18	49249996	rs323078	0.04982	t	c	-0.0072	0.2715
18	49244058	rs323080	0.04981	a	g	-0.0072	0.2722
18	49243635	rs323081	0.04582	t	c	0.0071	0.2725
18	49459266	rs12606702	0.03395	t	c	0.0047	0.2789
18	50187873	rs768204	0.00949	a	c	-0.0064	0.2798
18	50165405	rs147320414	0.01213	a	g	0.0081	0.2814
18	49265316	rs11876947	0.03501	a	g	0.007	0.2827
18	50231285	rs1160400	0.00735	a	g	0.0064	0.2834
18	49458174	rs2953265	0.02524	c	g	-0.0046	0.2863
18	49464724	rs35624144	0.02431	t	c	-0.0047	0.2867
18	50187843	rs768203	0.00954	t	c	0.0063	0.2888
18	49384374	rs1463585	0.02572	t	c	0.0068	0.2888
18	49239209	rs172829	0.04578	t	c	0.007	0.2895
18	49460260	rs6508101	0.03945	t	c	-0.0046	0.2898
18	49454047	rs8096258	0.03538	c	g	0.0046	0.2899
18	50189408	rs4387680	0.00946	t	c	-0.0063	0.2904
18	49350277	rs12457252	0.03687	a	t	-0.0052	0.3006
18	50189895	rs12458018	0.00903	t	g	0.0061	0.3033

18	49351749	rs1383525	0.03687	a	g	0.0051	0.308
18	49437082	rs2219926	0.04383	t	c	0.0062	0.3098
18	50204506	rs72929163	0.00929	a	t	0.0059	0.3099
18	49326609	rs822069	0.01008	a	g	-0.0052	0.3121
18	49320789	rs905732	0.01046	t	g	-0.0052	0.3129
18	50217774	rs12455373	0.00720	t	c	-0.0058	0.3179
18	49442033	rs2953271	0.01196	a	t	0.0043	0.3194
18	50217735	rs12455371	0.00772	c	g	0.0058	0.3196
18	50212100	rs8084402	0.00799	a	t	0.0058	0.3219
18	50208831	rs72914224	0.00770	t	c	-0.0058	0.3229
18	50211259	rs11661813	0.00766	a	g	0.0058	0.3237
18	50212069	rs8084407	0.00799	t	c	-0.0057	0.3253
18	50190193	rs1841302	0.01119	a	g	0.0059	0.3257
18	50160583	rs76925895	0.01069	t	c	-0.0095	0.3259
18	50224972	rs72914255	0.00701	t	c	0.0057	0.3287
18	50206480	rs766372	0.00933	t	c	-0.0057	0.3292
18	49436798	rs936638	0.04302	a	c	-0.0059	0.3299
18	49325699	rs1094478	0.02613	a	g	0.005	0.3307
18	49297654	rs7230877	0.00845	t	c	-0.005	0.3328
18	50071037	rs3910706	0.04860	t	c	0.0083	0.3331
18	49428003	rs2339282	0.02678	a	g	0.0058	0.3359
18	50198618	rs72929152	0.00962	c	g	-0.0056	0.3435
18	50205965	rs17681963	0.01085	a	g	-0.0055	0.3464
18	49430320	rs1351434	0.02810	a	g	0.0056	0.3547
18	49424276	rs1351433	0.02667	t	g	0.0055	0.3676
18	49322997	rs822070	0.00911	t	c	-0.0046	0.3694
18	49322747	rs822071	0.00904	a	c	-0.0046	0.3739
18	49322478	rs822072	0.00894	a	t	-0.0046	0.3744
18	49420854	rs1383528	0.02650	a	g	0.0054	0.3766
18	49452887	rs1351428	0.01186	t	g	-0.0037	0.397
18	49448657	rs2928943	0.01162	a	c	-0.0036	0.4033
18	50222524	rs66893782	0.01701	a	c	0.0045	0.411
18	49444855	rs2928941	0.02262	a	g	-0.0037	0.4138
18	49440953	rs2928937	0.01124	t	g	-0.0035	0.4182

18	49449088	rs2953268	0.01303	a	g	0.0035	0.4218
18	50527213	rs7237308	0.03470	t	c	-0.0038	0.4285
18	49447923	rs2953269	0.01093	t	c	-0.0034	0.4294
18	50100024	rs9946542	0.04909	a	g	0.0072	0.4332
18	50527261	rs7236076	0.03467	a	g	-0.0037	0.4339
18	49746483	rs16954498	0.03860	a	g	-0.0045	0.4344
18	49443797	rs2928940	0.01303	t	c	0.0034	0.4363
18	50497106	rs9955796	0.01739	a	c	0.0035	0.4622
18	50230968	rs59858381	0.03195	a	g	0.0047	0.4653
18	50116758	rs1563224	0.04900	t	c	-0.0065	0.4775
18	50207167	rs16920	0.01761	a	g	0.0038	0.4807
18	50496319	rs4940229	0.01193	a	g	0.0034	0.4817
18	50117398	rs1563225	0.04898	a	c	0.0064	0.4858
18	50495420	rs6508180	0.01193	t	c	-0.0033	0.4928
18	50185457	rs17749090	0.04370	t	c	0.0051	0.5069
18	49321773	rs10468869	0.02309	a	g	0.0034	0.5138
18	50176425	rs113619550	0.01939	t	c	-0.0051	0.5219
18	49015568	rs79844901	0.01957	c	g	-0.0054	0.5481
18	50593270	rs74384873	0.01587	a	g	-0.0178	0.571
18	50465151	rs78914372	0.01713	t	c	-0.0048	0.5728
18	50469542	rs16955886	0.01158	a	g	0.0047	0.5756
18	50469902	rs16955887	0.01167	t	c	-0.0047	0.5776
18	49306420	rs66516158	0.02599	t	c	-0.0032	0.5814
18	49233982	rs1699921	0.03375	a	g	-0.0037	0.583
18	50465225	rs77078075	0.01713	t	c	-0.0046	0.5839
18	49227118	rs149873166	0.03382	c	g	-0.0037	0.586
18	49237285	rs1699926	0.03375	c	g	-0.0037	0.5863
18	49227194	rs9748910	0.03125	t	g	-0.0037	0.588
18	49236518	rs1613312	0.03374	t	c	0.0036	0.5907
18	50136240	rs79696745	0.03110	t	g	-0.0053	0.5976
18	50584216	rs59494746	0.00919	t	c	-0.0159	0.6128
18	49256242	rs2615548	0.03373	a	c	-0.0033	0.621
18	49260268	rs323098	0.03373	c	g	0.0032	0.6322
18	50136443	rs113534738	0.03105	c	g	0.0047	0.6349

18	50472004	rs1943134	0.01710	t	c	-0.004	0.6394
18	49017661	rs76586136	0.03756	a	g	0.0041	0.6433
18	50471148	rs114707458	0.01710	a	g	-0.0039	0.6456
18	50464891	rs16955873	0.01978	a	c	-0.0038	0.6506
18	49325360	rs1094479	0.02596	t	c	0.0026	0.6547
18	49294002	rs7231613	0.02644	t	c	0.0037	0.6577
18	49128693	rs75045941	0.00155	t	c	0.0029	0.6589
18	49128436	rs11875823	0.00155	a	g	-0.0028	0.6614
18	50016704	rs11082928	0.04358	t	c	0.0019	0.6615
18	50475207	rs116513496	0.01714	t	c	-0.0037	0.6628
18	49333674	rs822068	0.01199	t	c	0.0024	0.6752
18	50475968	rs77897356	0.01709	a	g	-0.0035	0.6788
18	50475098	rs117889257	0.01714	a	g	0.0035	0.6794
18	50463966	rs80098946	0.01687	a	g	-0.0033	0.6937
18	49322126	rs10468871	0.02224	a	c	0.0025	0.6961
18	50597837	rs8098813	0.01930	t	g	0.0114	0.7047
18	50163961	rs113448791	0.01627	a	g	-0.0031	0.7093
18	49322167	rs822074	0.02901	t	c	0.0021	0.718
18	50494847	rs75966032	0.01316	a	t	0.0028	0.7266
18	49289204	rs8090172	0.04045	t	c	-0.0029	0.7281
18	49287823	rs72912792	0.04042	t	c	-0.0029	0.7288
18	50491200	rs117836641	0.01316	c	g	-0.0028	0.7291
18	50491145	rs115814478	0.01316	t	c	0.0028	0.7293
18	50490848	rs78640288	0.01316	t	c	0.0027	0.7328
18	49321853	rs10468870	0.02457	t	c	0.002	0.7333
18	50486990	rs115812024	0.01314	t	c	0.0027	0.7354
18	50502499	rs117050545	0.01318	t	c	0.0026	0.7426
18	50486907	rs115576991	0.01317	a	c	-0.0026	0.7451
18	50576977	rs57950523	0.00902	a	t	-0.0098	0.7466
18	50576983	rs59681356	0.00902	t	g	0.0098	0.7467
18	50231787	rs898438	0.01120	a	g	0.0014	0.7575
18	50125842	rs34234743	0.03026	a	g	0.0018	0.7605
18	49128658	rs8087777	0.02278	a	t	0.0017	0.7655
18	49456200	rs9948625	0.02274	t	c	-0.0016	0.7874

18	49130122	rs6508077	0.02209	t	c	0.0015	0.7959
18	49451782	rs75127496	0.03315	t	c	-0.0012	0.8049
18	49418404	rs1383527	0.00609	a	g	-0.0011	0.8059
18	50509525	rs73956750	0.01614	a	g	0.0016	0.8434
18	50761689	rs16956399	0.02388	a	g	-0.0014	0.8596
18	49940973	rs9956738	0.03927	a	g	-0.0031	0.8685
18	50726298	rs55651659	0.04604	a	g	-0.0011	0.8895
18	49389900	rs58394960	0.02521	a	g	-6.00E-04	0.892
18	50726573	rs8085132	0.00708	t	c	0.0011	0.8931
18	50742446	rs55915244	0.00817	t	c	-0.001	0.8965
18	49439925	rs2928933	0.04414	a	g	7.00E-04	0.8969
18	50723324	rs61480191	0.01066	a	g	0.001	0.8977
18	50711414	rs10502962	0.00981	a	g	0.001	0.9021
18	50742230	rs66706659	0.00998	a	g	-0.001	0.9038
18	50706722	rs16956299	0.03745	a	g	0.001	0.9042
18	50727720	rs35520310	0.01078	a	g	9.00E-04	0.9049
18	50320622	rs7232287	0.01647	a	t	-6.00E-04	0.9052
18	50707411	rs72921499	0.03745	a	t	9.00E-04	0.9056
18	50704839	rs17408063	0.04499	c	g	9.00E-04	0.9118
18	50703914	rs10502961	0.01355	c	g	-9.00E-04	0.9128
18	50708446	rs16956305	0.01359	a	g	9.00E-04	0.9132
18	50126784	rs8095889	0.04965	t	c	-7.00E-04	0.9136
18	49459622	rs75540970	0.01906	t	g	-6.00E-04	0.9151
18	50712747	rs7238728	0.02707	a	g	7.00E-04	0.9221
18	50708712	rs17408365	0.04528	a	t	7.00E-04	0.9257
18	49117741	rs9963327	0.04450	a	t	-5.00E-04	0.9277
18	50734990	rs12326120	0.00996	a	g	-7.00E-04	0.9324
18	50696696	rs72921480	0.01352	t	g	7.00E-04	0.9337
18	50750875	rs7243756	0.01022	a	g	-6.00E-04	0.9424
18	50757382	rs16956392	0.01303	t	c	5.00E-04	0.9492
18	50751215	rs7238231	0.01017	a	g	5.00E-04	0.9503
18	50713748	rs7243864	0.02709	t	c	4.00E-04	0.9503
18	50754083	rs13339708	0.00989	t	c	-5.00E-04	0.9525
18	50749396	rs7237110	0.01042	a	t	5.00E-04	0.9531

18	50320520	rs7233401	0.01646	t	c	-3.00E-04	0.9534
18	50464904	rs75816960	0.02899	a	g	-4.00E-04	0.9595
18	50716370	rs6508205	0.02519	t	c	-3.00E-04	0.9649
18	50745120	rs60234723	0.01051	t	c	-3.00E-04	0.9744
18	50743736	rs7243973	0.01042	t	g	-2.00E-04	0.9751
18	50709755	rs8094601	0.04009	a	g	-2.00E-04	0.9779
18	50714232	rs16956330	0.02572	t	c	-2.00E-04	0.9815
18	50710619	rs16956311	0.02680	c	g	1.00E-04	0.9829
18	50745422	rs16956378	0.01051	a	g	-2.00E-04	0.9834
18	50710072	rs8099181	0.04013	t	g	1.00E-04	0.9869
18	50714276	rs72923213	0.02572	a	g	0	0.9968
18	50222796	rs7236766	0.03457	t	g	0	0.9975
18	50694098	rs16956260	0.01306	a	g	0	0.9999

Table S3. DCC eQTL SNPs in fetal brains from the UCLA Gene and Cell Therapy core and their associations with depression							
**SNP_CHR**	**SNP_BP**	**SNP_rsID**	**DCC_eQTL_P**	**GWAS_A1**	**GWAS_A2**	**GWAS_LogOR**	**GWAS_P**
18	50507006	rs11663596	0.000229501	t	c	-0.0208	2.60E-06
18	50831134	rs6508219	0.00002	a	t	-0.0339	2.96E-06
18	50837474	rs7505903	0.000104808	t	c	0.0332	3.40E-06
18	50831643	rs6508221	0.000475507	a	g	-0.033	5.21E-06
18	50852954	rs17503301	0.0000924	a	g	-0.0316	9.40E-06
18	50400446	rs11665486	0.000529468	t	c	0.0188	1.72E-05
18	50816389	rs6508217	0.0000119	a	g	-0.031	5.01E-05
18	50824753	rs7407520	0.000444166	a	g	0.0309	5.08E-05
18	50769888	rs7232783	0.0000529	t	c	-0.0306	6.11E-05
18	50812172	rs6508216	0.000430455	a	g	-0.0302	7.70E-05
18	50758952	rs16956398	0.0000546	a	g	0.0299	8.32E-05
18	50695904	rs9949444	0.000363144	a	c	0.0164	0.0001959
18	50430412	rs2156284	0.000179209	c	g	-0.0114	0.008674
18	50485926	rs12959551	0.00032753	t	c	0.0108	0.01239
18	50561632	rs8085965	0.000486278	t	g	0.0108	0.01608
18	50565946	rs7505888	0.0000929	t	c	-0.0107	0.01661
18	50566190	rs7505807	0.00019691	c	g	0.0107	0.01704
18	50556725	rs8088299	0.0000714	t	g	-0.0106	0.01801
18	50570638	rs6508188	0.000215358	t	g	-0.0105	0.0183
18	50444667	rs12457812	0.000355773	t	c	-0.0101	0.02128
18	50571241	rs9949949	0.000215225	a	g	-0.0103	0.02171
18	50413594	rs1431740	0.000235686	c	g	-0.01	0.02334
18	50576312	rs7506999	0.000215807	t	g	-0.01	0.02511
18	50576221	rs7506784	0.000213832	a	t	-0.01	0.02537
18	50440067	rs12968712	0.000188602	a	g	-0.0096	0.02971
18	50549521	rs6508186	0.000344609	t	c	0.0093	0.0312
18	50548544	rs9950125	0.000367966	a	g	-0.0092	0.03341
18	50436661	rs1541287	0.000188602	t	c	-0.0093	0.03354
18	50427717	rs6508176	0.000215759	t	c	-0.0091	0.03958
18	50448761	rs12966311	0.0000336	t	c	0.0092	0.03978
18	50542722	rs61537869	0.000326183	a	g	-0.0083	0.05717

18	50574947	rs13381767	0.000116968	a	g	0.0084	0.06144
18	50489677	rs12957778	0.0000622	a	g	0.008	0.06975
18	50463278	rs12458638	0.000284458	a	g	0.0079	0.07977
18	50103952	rs62083388	0.000509588	a	c	0.007	0.1157
18	50119836	rs72914715	0.00042352	a	t	-0.0067	0.1289
18	50088019	rs7231796	0.000513013	a	c	0.0067	0.1321
18	50098209	rs8094588	0.000367977	t	c	-0.0066	0.1388
18	50109875	rs72913000	0.000540831	a	g	-0.0065	0.14
18	50118687	rs62083396	0.000463504	a	t	-0.0064	0.1494
18	50085350	rs10502948	0.000519005	t	g	0.0064	0.1506
18	50085403	rs28635403	0.000519358	t	c	0.0063	0.1516
18	50082150	rs8092948	0.000519005	a	c	0.0063	0.1569
18	50083383	rs1442350	0.000519005	a	g	0.0063	0.1574
18	50395036	rs12455180	0.00000643	t	c	-0.0064	0.1575
18	50092694	rs62083383	0.000405863	a	g	-0.0061	0.1712
18	50078406	rs62083380	0.000128285	c	g	0.004	0.3654
18	50062945	rs2156126	0.000120829	t	c	0.0037	0.3976
18	50071870	rs7227557	0.000120604	c	g	0.0035	0.4301
18	50051667	rs62081335	0.000126857	a	g	-0.0029	0.5149
18	50051109	rs62081333	0.000127246	a	g	-0.0028	0.5198
